# Spiking neural models for decision-making tasks with learning

**DOI:** 10.1007/s00285-026-02415-0

**Published:** 2026-06-12

**Authors:** Sophie Jaffard, Giulia Mezzadri, Patricia Reynaud-Bouret, Etienne Tanré

**Affiliations:** 1 Center for Systems Biology Dresden, Max Planck Institute of Molecular Cell Biology and Genetics, Dresden, Germany; 2 Centre Borelli, École Normale Supérieure Paris-Saclay, Paris, France; 3 Laboratoire J. A. Dieudonné, CNRS, Université Côte d’Azur, Nice, France; 4 Université Côte D’Azur, Inria, CNRS, LJAD, Nice, France

**Keywords:** Spiking Neural Network, Decision-making, Hawkes process, Local learning rule, Drift diffusion model, 60G55, 68T05, 60F15, 92-10

## Abstract

In cognition, response times and choices in decision-making tasks are commonly modeled using Drift Diffusion Models (DDMs), which describe the accumulation of evidence for a decision as a stochastic process, specifically a Brownian motion, with the drift rate reflecting the strength of the evidence. In the same vein, the Poisson counter model describes the accumulation of evidence as discrete events whose counts over time are modeled as Poisson processes. This model has a spiking neurons interpretation as these processes are used to model neuronal activities. However, these models lack a learning mechanism and are limited to tasks where participants have prior knowledge of the categories. To bridge the gap between cognitive and biological models, we propose a biologically plausible Spiking Neural Network (SNN) model for decision-making that incorporates a learning mechanism and whose neurons activities are modeled by a multivariate Hawkes process. First, we show a coupling result between the DDM and the Poisson counter model, establishing that these two models provide similar categorizations and reaction times and that the DDM can be approximated by spiking Poisson neurons. To go further, we show that a particular DDM with correlated noise can be derived from a Hawkes network of spiking neurons governed by a local learning rule. In addition, we designed an online categorization task to evaluate the model predictions. This work provides a significant step toward integrating biologically relevant neural mechanisms into cognitive models, fostering a deeper understanding of the relationship between neural activity and behavior.

## Introduction

One of the main challenges of the 21st century is to bridge the gap between brain and behavior, and computational models are a fundamental tool researchers use to address this challenge. In this line, developing biologically realistic models to understand the decision-making processes of humans and animals is a key focus in neuroscience and cognition.

A prominent model in cognition is the Drift Diffusion Model (DDM), introduced by Ratcliff (1978, 1981, 1988) who demonstrated how the DDM could accurately predict outcomes such as accuracy, reaction times, and error latencies across various decision-making tasks. The model achieves this by integrating evidence over time, with a drift rate reflecting evidence strength and decision boundaries representing the thresholds for making a decision. Initially designed for two-choice experiments, it extends to multiple choices by modeling evidence accumulation as a multidimensional drift-diffusion process, with each component competing against the others (Roxin 2019). The reasons for the growing attention to the DDM are multiple. It has been shown to provide accurate descriptions of accuracy and reaction time data across a wide range of psychological tasks, including perceptual tasks (Ratcliff and Rouder 1998; Ditterich 2006; Brunton et al. 2013) and value-based choices (Ratcliff and McKoon 2008; Philiastides and Ratcliff 2013; Hutcherson et al. 2015).

It is also supported by neurobiological evidence: in the 1990s and 2000s, neurobiologists like Shadlen and Newsome provided crucial neurobiological evidence supporting the DDM by showing that neural activity in brain areas such as the lateral intraparietal area (LIP) reflects the accumulation of sensory evidence, consistent with DDM predictions (Shadlen and Newsome 1996; Gold and Shadlen 2001; Ratcliff et al. 2007; Shadlen and Kiani 2013). However, DDMs do not directly include the modeling of neuronal activity.

Biologically relevant decision-making models (Brody et al. 2003; Machens et al. 2005; Deco and Martí 2007) rely on perceptual information to guide the choice between behaviors. A straightforward approach involves two interacting groups of neurons, each defined by its average firing rate. Decision-making in this context is modeled as a transition from a spontaneous state (where both groups have similar firing rates) to a decision state (characterized by a high or low activity ratio between the groups), and this process is often represented as a stochastic dynamical system. Such models effectively approximate reaction times and can be used to compute biologically meaningful information. For example, Carrillo et al. (2011) offer a mathematical analysis of these systems in nonlinear scenarios and computes the probability of reaching specific decisions and the average time required to do so. Other approaches than evidence accumulation can be implemented in such models: for instance, Insabato et al. (2010) propose a decision confidence mechanism. However, none of these models are linked to the DDM nor show such accurate descriptions of real accuracy or reaction times.

Simpler than these models and more similar to the Drift Diffusion Model, the Poisson counter model (LaBerge 1994; Ratcliff and Smith 2004) represents evidence accumulation as discrete events, with their counts over time modeled as homogeneous Poisson processes. This approach aligns with how neuroscientists often use Poisson processes to model neuronal activity based on firing rates, offering a spiking neuron interpretation: evidence accumulation can be seen as the spike counts of neurons coding for potential responses. Smith and Van Zandt (2000) extend the counter model by incorporating inhomogeneous Poisson processes. However, since the Poisson process is not well-suited for modeling the interactions between neurons, this approach is limited to representing individual neurons coding for categories and cannot capture the dynamics of more complex networks of spiking neurons.

A common tool to model spike trains of interacting neurons is the multivariate Hawkes process (Hawkes 1971), which is a self-exciting and mutually exciting point process. Its field of application is not limited to neuroscience: it is well-adapted to model earthquake data (Türkyilmaz et al. 2013), financial transactions (Bacry et al. 2015), health data (Bao et al. 2017), social networks (Zhou et al. 2013), or more generally, any sequences of events such that the occurrence of an event influences the probability of further events to occur.

Poisson and Hawkes processes seem to be promising microscopic processes to explain the power of the DDM prediction. Indeed diffusion approximations of Poisson and Hawkes processes have been investigated in several contexts. For instance, Bacry et al. (2013) provide a functional central limit theorem for multivariate Hawkes processes for finance applications, and Ethier and Kurtz (1986) as well as Bretagnolle and Massart (1989) provide strong approximations of the Poisson process by a Brownian process. Strong approximations by diffusive limits have also been derived to study mean field limits of networks of interacting neurons (Erny et al. 2023), and Besançon et al. (2024) provide coupling results between Poisson and Hawkes processes in one dimension. Additionally, Chevallier et al. (2021) establish strong error bounds and numerical schemes for the diffusion approximation of multi-class Hawkes processes.

However, neither the DDM, the Poisson counter model, nor the other biologically relevant decision-making models discussed here incorporate a learning mechanism. These models are limited to tasks where participants already possess prior knowledge of the possible responses. In the DDM, for instance, this prior knowledge is represented by the value of the drift rate, while in stochastic dynamical systems, it is captured by the (fixed) strength of synaptic connections. For example, Deco and Martí (2007) assume that synaptic connections between neurons are the result of a Hebbian learning mechanism that has taken place before the model they propose.

On the other hand, learning mechanisms are at the heart of artificial neural networks (ANNs), the first of which is the perceptron (Rosenblatt 1957), inspired by the structure and function of biological neurons and their networks. This biological inspiration has continued to influence the development of increasingly advanced machine learning models, such as convolutional neural networks (Le Cun et al. 1989), which draw their inspiration from biological processes observed in the visual cortex by Hubel and Wiesel (1962). Conversely, machine learning algorithms play a central role in implementing spiking neural networks (SNNs) (Tavanaei et al. 2019). These networks, more complex than ANNs, model neuronal activity through sequences of spikes, mimicking the electrical pulses of biological neurons. Particularly relevant for studying the brain’s neural code, SNNs also offer insights into the design of energy-efficient algorithms (Stone 2018). To be realistic, SNNs are typically trained using local learning rules such as Hebbian learning (Hebb 2005) or spike-timing-dependent plasticity (Caporale and Dan 2008). However, spiking neural networks (SNNs) are typically not designed to model decision-making tasks. They do not inherently account for reaction times and are primarily optimized for achieving strong empirical performance by mimicking the functioning of the brain rather than making decisions that closely resemble human behavior.

The focus of the present work is to bridge the gap between decision-making models used in cognition and brain-inspired models of spiking neurons, and to establish that prior knowledge assumed in decision-making models may be the result of a biologically relevant learning mechanism. First, we introduce both a drift diffusion model and a Poisson counter model, outlining conditions under which each produces accurate decisions (Theorems 2.3 and 2.5). As an initial step toward deriving the DDM from spiking neuron-based models, we establish a coupling between the two models, establishing that they provide similar categorizations and reaction times (Theorem 2.6). This approximation holds in the asymptotic case where the number of neurons is large, which is consistent with brain recordings indicating that concepts are often encoded by coordinated groups of neurons, commonly referred to as neuronal assemblies (Singer et al. 1997; Gerstein et al. 1989).

To go further, we introduce a biologically inspired Spiking Neural Network (SNN) model for decision-making. This model integrates a learning mechanism and is provably close to the drift diffusion model. Neuronal activity within the network is represented by a multivariate Hawkes process, which accounts for interactions between neurons. The output neurons encode possible decisions, with their spike counts representing evidence accumulation for respective responses, which makes our model a Hawkes counter model. Synaptic connections are adjusted via a local learning rule provided by the expert aggregation framework (Cesa-Bianchi and Lugosi 2006). In Jaffard et al. (2024), we introduced a simpler version of the network. The focus of this work was to prove theoretically that a biologically plausible neural network could learn using local mechanisms only. Building on this foundation, Jaffard et al. (2026) extended the network by incorporating hidden layers specifically designed to detect neuronal synchronizations. This extension not only advanced the theoretical results from Jaffard et al. (2024) but also demonstrated that our algorithm automatically produces neuronal assemblies in the sense that the network can encode several concepts and that the same neuron in the intermediate layers might be activated by more than one concept. Additionally, we provided an in-depth analysis of the types of concepts the network can encode and numerical results on handwritten digits dataset. However, this initial version was not suited to model decision-making tasks as it was unable to model reaction times.

The model presented in the current work is a refined version of this network, which introduces two key differences from these earlier versions. First, neuronal activities are modeled in continuous time rather than discrete time, which is more realistic and closer to the drift diffusion model and Poisson counter model. Second, we have incorporated dynamic decision durations, allowing the model to account for reaction times alongside decision-making processes, unlike the fixed decision durations used previously. These improvements enable the model to be compared with other models such as the DDM or the Poisson counter model.

We analyze the network’s asymptotic behavior (Proposition 3.3) and provide conditions under which the model delivers accurate decisions (Theorem 3.5). Additionally, we establish a coupling between our model and the DDM (Theorem 3.6) thanks to strong approximations similar to Ethier and Kurtz (1986), demonstrating that a DDM with correlated noise can be approximated by a Hawkes network of spiking neurons capable of learning via local rules.

To evaluate our model, we designed an online categorization task in which participants were asked to classify stimuli – rockets in this case – defined by four characteristics, with one randomly selected feature serving as the basis for learning. Such feature-based tasks have long been central to cognitive science (Nosofsky 1986; Love et al. 2004), providing valuable insights into how individuals learn to group stimuli based on shared characteristics. This well-established paradigm made it a natural starting point for testing our model. By analyzing participants’ reaction times and choices, we demonstrate the utility of the Hawkes counter model in studying learning and decision-making processes. We describe the experiment we designed and detail its implementation within our model. We prove that the network can learn to perform this task successfully (Proposition 4.1). Furthermore, we estimate model parameters for individual participants, enabling a detailed analysis of their learning behaviors.

Section 2 introduces the decision-making task we aim to model, along with the drift diffusion model and the Poisson counter model, including their mathematical analyses and coupling. Section 3 outlines the Hawkes counter model for decision-making tasks with learning and its corresponding mathematical analysis and coupling with the DDM. Section 4 details our experiment and its analysis based on our model. All notations defined in the main text are listed in Appendix A, and any proofs not included in the main text are provided in Appendix C.

## Drift diffusion and poisson counter models

### Framework

**Fig. 1:**
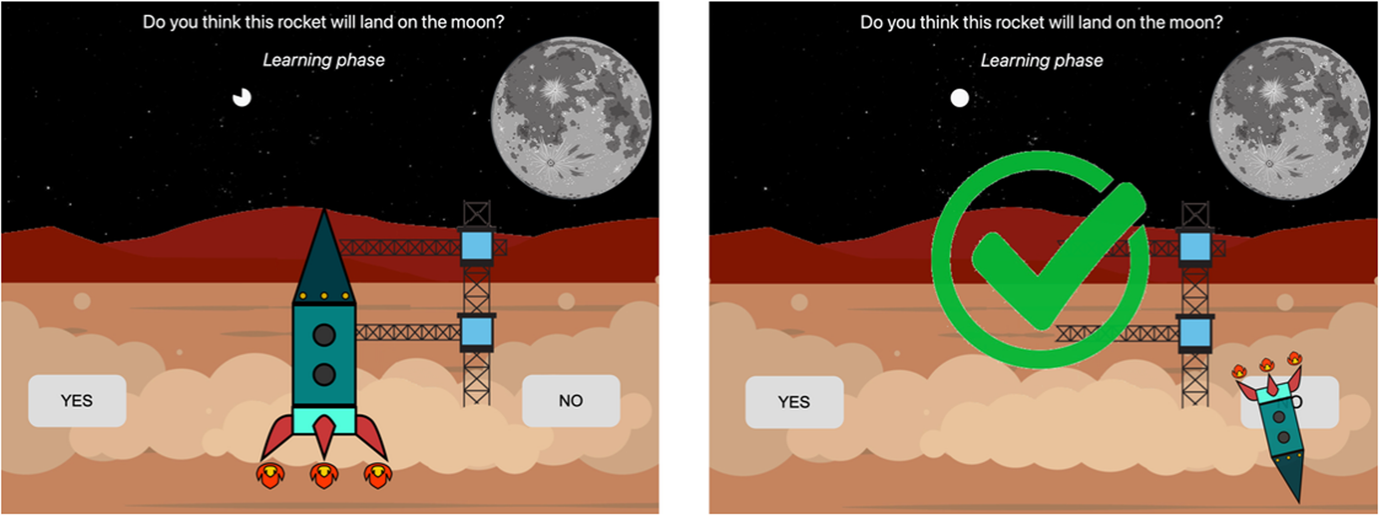
Illustration of our online experiment available at https://3ia-demos.inria.fr/mel/en/. The participant has to learn to classify rockets into two categories: those that can land on the moon and those that cannot. They are successively presented with rockets for which they have to propose a category (left picture). After each categorization, feedback is provided on their answers (right picture)

**Fig. 2:**
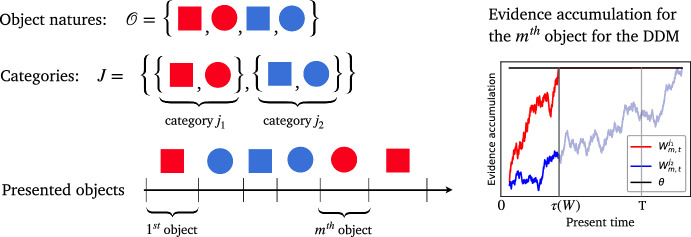
Illustrative example of objects natures, categories, presented objects and evidence accumulation for the $m^{th}$ presented object for the DDM. Here, there are 4 object natures, 2 categories and 6 presented objects. The chosen response $\hat{\jmath }$ is the category coded by the first process to reach threshold θ, *i.e.,* $\hat{\jmath }= j_1$ and the corresponding reaction time is τ(W)

We aim to model first the following decision-making task: a participant sequentially categorizes objects, each having a nature from a set $\mathcal {O}$, into one of several categories belonging to a set *J*. Each category j∈J is a subset of $\mathcal {O}$, and together the categories form a partition of $\mathcal {O}$. Participants may encounter the same object nature multiple times; for example, the first object presented might be a blue circle, as might the tenth object. See Figure 1 for an illustration of the categorization task studied in Section 4, and Figure 2 for another example.

In the present section, we present two models: the drift diffusion model and the Poisson counter model. They provide predictions for both categorizations and reaction times. These models assume that participants already possess prior knowledge about object natures and categories, and do not describe scenarios where participants must learn the categories of the presented objects.

For both models, we give explicit conditions under which they predict correct categorizations with high probability. Then, we prove a coupling result between the models indicating that these two models provide very similar reaction times and categorizations.

For $a,b\in \mathbb {R}$, we denote $a\vee b :=\max (a,b)$ and $a\wedge b :=\min (a,b)$. Also when quantities have several indices, the dropping of one index corresponds to the sequence on this index. For instance, $\gamma = (\gamma ^j_o)_{j\in J, o\in \mathcal {O}}$ whereas $\gamma _o = (\gamma ^j_o)_{j\in J}$ and $\gamma ^j = (\gamma ^j_o)_{o\in \mathcal {O}}$. If two sets of indices are available, typically *I* and *J*, to avoid confusion, we use the set instead of the index. For instance, $\gamma ^I=(\gamma ^i_o)_{i\in I, o\in \mathcal {O}}$. Also $\Vert \cdot \Vert _{\infty }$ is the infinite norm.

### Drift Diffusion model

#### The model

During the presentation of the $m^{th}$ object, the accumulation of evidence of the categories j∈J is modeled by a |J|-dimensional drifted Brownian motion $W_m = (W^j_{m})_{j\in J}$ with mean vector defined by $\forall j\in J, \forall t\ge 0$, $\mathbb {E}[W^j_{m,t}] = \mu ^j_o t$ and diagonal covariance matrix defined by $\forall j_1,j_2 \in J$, $\forall t,s\ge 0$, $\mathop {\text {Cov}}(W^{j_1}_{m,t}, W^{j_2}_{m,s}) = 1\!\!1_{\{j_1 = j_2\}} \mu ^{j_1}_o (t\wedge s)$ where *o* is the nature of the $m^{th}$ object and $\mu ^j_o\ge 0$ is the drift. Therefore, the process coding for evidence accumulation in favor of category *j* can be rewritten as

1 $$\begin{aligned} \forall t\ge 0, \quad W^j_{m,t} :=\mu ^j_o t + \sqrt{\mu ^j_o} B^j_{m,t} \end{aligned}$$

where the $(B^j_{m,t})_{t\ge 0}$ are independent standard Brownian motions. The object is categorized in the category coded by the first process $(W^j_{m,t})_{t\ge 0}$ among the processes of $W_m$ to reach a certain threshold θ>0 before a limit duration *T*. We denote

2 $$\begin{aligned} \tau (W^j):=\inf \{u\ge 0; W^j_{m,u} \ge \theta \}. \end{aligned}$$

##### Remark 2.1

The law of $\tau (W^j)$ is the inverse Gaussian Distribution with parameters $\frac{\theta }{\mu ^j_o}$ and $\frac{\theta ^2}{\mu ^j_o}$ (Lovric (2025), p.687).

The winning process is then

3 $$\begin{aligned} \hat{\jmath }= \arg \min _{j\in J} \tau (W^j). \end{aligned}$$

Note that $\hat{\jmath }$ and $\tau (W^j)$ depend on *m* but we drop the index for simplicity. However, if $\tau (W^{\hat{\jmath }})\ge T$, it means that accumulation evidence is not sufficient to reach a decision and the model does not categorize the object. The corresponding reaction time of the whole system is then $\tau (W) :=\tau (W^{\hat{\jmath }})\wedge T$. Note that the processes are indexed by time t∈[0,T] which is not a ‘time’, but a ‘present time’ since it is set back to 0 at each presentation of a new object. See Figure 2 for an example of evidence accumulation modeled by the DDM.

Note that in general, in drift diffusion models, the scaling is not necessarily the square root of the drift. We study this specific case because it allows the approximations by spiking neural models that we present further on. Note also that the drift and scaling do not evolve with the number of presented objects *m*: they represent prior knowledge about the categories and object natures and are fixed. The higher the drift $\mu ^j_o$, the higher the average accumulation of evidence in favor of category *j*.

#### Theoretical guarantees

Before detailing the specific conditions under which the model predicts the correct category, it is worth recalling that the fundamental relationships between decision time, accuracy, and evidence accumulation parameters are already well-established in the literature. For instance, Wagenmakers et al. (2007) demonstrated through the EZ-diffusion model that there are explicit, closed-form equations directly linking observable accuracy and response times to the underlying drift rate and decision threshold. Furthermore, the joint distribution of choice probabilities and decision times under time constraints has been rigorously analyzed; Fudenberg et al. (2018) mapped the exact conditions and optimal sequential sampling policies required to guarantee accurate choices within specific time windows. While the results establishing the conditions for correct prediction in drift-diffusion processes are known, the following theorem builds upon this theoretical tradition to establish the specific margin conditions required for the present framework.

Let us study under which conditions the model predicts the correct category. We assume that the participant has prior knowledge about the categories, which takes the form of the following assumption. Let ${\boldsymbol{\Delta }}_{\mu }>0$.

##### Assumption 2.2

*(Margin(*${\boldsymbol{\Delta }}_{\mu }$*))* For every category $j^*\in J$, for every $j\ne j^*$, for every object nature $o\in j^*$ we have

$$ \mu ^{j^*}_o > \mu ^{j}_o + {\boldsymbol{\Delta }}_{\mu }. $$

##### Theorem 2.3

Let $\alpha \in (0,1)$ and let *m* be a fixed positive integer. Let ${\boldsymbol{\Delta }}_{\mu }>0$ and suppose Assumption 2.2 (Margin(${\boldsymbol{\Delta }}_{\mu }$)) holds. Then there exist positive constants $A_{\Vert \mu \Vert _\infty , {\boldsymbol{\Delta }}_{\mu }}, B_{\Vert \mu \Vert _\infty , {\boldsymbol{\Delta }}_{\mu }}$ and $C_{\Vert \mu \Vert _\infty , {\boldsymbol{\Delta }}_{\mu }}$ depending only on the index parameters such that if $T \ge A_{\Vert \mu \Vert _\infty , {\boldsymbol{\Delta }}_{\mu }} \log \left( |J |\alpha ^{-1}\right)$ and if the threshold θ satisfies

4 $$\begin{aligned} B_{\Vert \mu \Vert _\infty , {\boldsymbol{\Delta }}_{\mu }} \log \left( |J |\alpha ^{-1}\right)< \theta < {\boldsymbol{\Delta }}_{\mu }T - C_{\Vert \mu \Vert _\infty , {\boldsymbol{\Delta }}_{\mu }} \sqrt{T\log (T) + \log \left( |J |\alpha ^{-1}\right) } \end{aligned}$$

then with probability larger than 1-α we have that for all nature $o\in \mathcal {O}$, if we denote by $j^*\in J$ its category (i.e. $o\in j^*$), the choice $\hat{\jmath }$ (3) and the reaction time $\tau (W^{\hat{\jmath }})$ (2) that take place at the presentation of the $m^{th}$ object of nature *o*, satisfy

$$\begin{aligned} \hat{\jmath }= j^* \quad \text{ and }\quad \tau (W^{\hat{\jmath }}) < T. \end{aligned}$$

See Appendix C.2 for a version of this result with exact constants.

In other words, this theorem states that for a fixed object *m*, if the limit duration *T* is large enough then as soon as the threshold θ verifies (4), object *m* is correctly classified with probability more than 1-α. The lower bound comes from the condition that the process coding for category $j^*$ should be the first to reach θ, whereas the upper bound in *O*(*T*) comes from the condition that θ should be reached before limit duration *T*. Note that the exact expressions of the constants provided in Appendix C.2 indicate that as expected, the larger ${\boldsymbol{\Delta }}_{\mu }$, *i.e., *the larger the gap between the drift of the process coding for the true category and the other drifts, the lower the bound on *T* and the larger the size of the thresholds interval enabling a correct categorization with high probability.

### Poisson counter model

#### The model

For each presented object, the evidence in favor of the categories takes now the form of discrete events whose counts over time are modeled by a |J|-dimensional homogeneous Poisson process $\Pi _m = (\Pi ^j_m)_{j\in J}$ with intensity vector $\gamma _o :=(\gamma ^j_o)_{j\in J}$ depending only on the nature *o* of the presented object. Similarly as for the DDM, the object is categorized in the category coded by the first process $(\Pi ^j_{m,t})_{t\ge 0}$ among the processes of $\Pi _m$ to reach threshold θ>0 before limit duration *T*. We denote

5 $$\begin{aligned} \tau (\Pi ^j) :=\inf \{u\ge 0; \Pi ^j_{m,u}\ge \theta \} \end{aligned}$$

the hitting time of process $\Pi ^j_m$. The winning process is then

6 $$\begin{aligned} \hat{\jmath }:=\arg \min _{j\in J} \tau (\Pi ^j). \end{aligned}$$

However, if $\tau (\Pi ^{\hat{\jmath }})\ge T$, it means that evidence accumulation is not sufficient to reach a decision and the model does not categorize the object. Note that as before, the hitting time $\tau (\Pi ^{\hat{\jmath }})$ and choice $\hat{\jmath }$ depend on *m* but we drop the index for simplicity. The corresponding reaction time is then $\tau (\Pi ) :=\tau (\Pi ^{\hat{\jmath }})\wedge T$.

Similar to the DDM, here the intensities do not evolve with the number of presented objects *m*: they represent prior knowledge about the categories and object natures and are fixed. The higher the intensity $\gamma ^j_o$, the higher the average accumulation of evidence in favor of category *j*.

The Poisson process is commonly used to model the activities of spiking neurons coding information through their firing rate, and which do not interact as a network. Therefore, the Poisson counter model can have the following interpretation: each category *j* is coded by a spiking neuron *j* whose activity when presented with the $m^{th}$ object is $(\Pi ^j_{m,t})_{t\ge 0}$. The spike count of neuron *j* represents the accumulation of evidence in favor of category *j*.

#### Theoretical guarantees

Similar to the continuous diffusion framework, the fundamental conditions governing accuracy and response times in discrete Poisson counter models are already well-documented. Analytical solutions for choice probabilities and first-passage times in discrete race architectures have been extensively characterized, notably by Smith and Van Zandt (2000). Furthermore, the mathematical conditions under which one Poisson process reliably reaches a decision threshold before competing processes-as well as the associated bounds on error rates-rely on established combinatorial formulas and large deviation bounds. While these general mechanics of discrete evidence accumulation are known, the following theorem formalizes the specific intensity margin conditions required for correct prediction within the context of our proposed framework.

We can conduct a similar analysis as for the DDM by assuming a similar assumption representing prior knowledge about the categories. Let ${{\boldsymbol{\Delta }}_\gamma }>0$.

##### Assumption 2.4

*(Margin(*${{\boldsymbol{\Delta }}_\gamma }$*))* For every category $j^*\in J$, for every $j\ne j^*$, for every object nature $o\in j^*$ we have

$$ \gamma ^{j^*}_o > \gamma ^{j}_o + {{\boldsymbol{\Delta }}_\gamma }. $$

##### Theorem 2.5

Let $\alpha \in (0,1)$ and let *m* be a fixed positive integer. Let ${{\boldsymbol{\Delta }}_\gamma }>0$ and suppose Assumption 2.4 (Margin(${{\boldsymbol{\Delta }}_\gamma }$)) holds. Then there exist positive constants $A_{\Vert \gamma \Vert _{\infty }, {{\boldsymbol{\Delta }}_\gamma }, \gamma _{\text {min}}}$, $B_{\Vert \gamma \Vert _{\infty }, {{\boldsymbol{\Delta }}_\gamma }, \gamma _{\text {min}}}$ and $C_{\Vert \gamma \Vert _{\infty }}$ depending only on the index parameters such that if $T\ge A_{\Vert \gamma \Vert _{\infty }, {{\boldsymbol{\Delta }}_\gamma }, \gamma _{\text {min}}} \log (|J |\alpha ^{-1})$ and if the threshold θ satisfies

7 $$\begin{aligned} B_{\Vert \gamma \Vert _{\infty }, {{\boldsymbol{\Delta }}_\gamma }, \gamma _{\text {min}}}\log (|J |\alpha ^{-1})< \theta < {{\boldsymbol{\Delta }}_\gamma }T - C_{\Vert \gamma \Vert _{\infty }} \sqrt{T \log (|J |\alpha ^{-1})} \end{aligned}$$

where $\gamma _{\text {min}} :=\min _{o\in \mathcal {O},j\in J}\{\gamma ^i_o; \gamma ^j_o >0\}$, then with probability larger than 1-α we have that for all nature $o\in \mathcal {O}$, if we denote by $j^*\in J$ its category (i.e. $o\in j^*$), the choice $\hat{\jmath }$ (6) and the reaction time $\tau (\Pi ^{\hat{\jmath }})$ (5) that take place at the presentation of the $m^{th}$ object of nature *o*, satisfy

$$\begin{aligned} \hat{\jmath }= j^* \quad \text{ and } \quad \tau (\Pi ^{\hat{\jmath }})<T. \end{aligned}$$

See Appendix C.3 for a version of this result with exact constants.

Similarly as Theorem 2.3 about the DDM, this theorem provides conditions on the threshold θ under which the model predicts the correct category with high probability. Regarding the limit duration *T*, the upper bound has the same order of magnitude in *O*(*T*). Here, the lower bound depends on the quantity $\gamma _{\text {min}}$, which was not the case in Theorem 2.3. Finally, it should be noted that similarly as in Theorem 2.3, the exact constant values provided in Appendix C.3 indicate that the larger the constant ${{\boldsymbol{\Delta }}_\gamma }$, the larger the size of the possible thresholds interval.

### Coupling between drift diffusion model and poisson counter model

In this section, we explain the high similarity between the behaviors of the Poisson model and the Drift Diffusion model by proving that one model can be strongly approximated by the other and that both models provides similar reaction times, up to negligible terms. Indeed, under a certain framework, we can build a coupling between the processes given by the two models. Here, we assume that we dispose of *n* independent copies of the Poisson counter model: during the presentation of object *m*, each category *j* is coded by *n* independent Poisson processes, all having intensity $\gamma ^j_o$ where *o* is the nature of object *m*. The evidence in favor of category *j* is then the sum of the count of the *n* processes coding for category *j* and is denoted by $\Pi ^{j,n}_m = (\Pi ^{j,n}_{m,t})_{t\ge 0}$. By independence of the neurons coding for category *j*, the process $\Pi ^{j,n}_{m}$ is then a homogeneous Poisson process with intensity $n\gamma ^j_o$. The presented object is classified in the category coded by the first process $\Pi ^{j,n}_{m}$ among the family of processes $\Pi ^n_m:=(\Pi ^{j,n}_m)_{j\in J}$ to reach the threshold nθ (or not classified at all if none of the processes reaches nθ). We chose here the threshold to be proportional to *n* to allow the coupling to take place. Up to this modification, the decision-making process is identical to (5) and (6).

The consideration of *n* copies of the Poisson counter model is motivated by both mathematical and biological considerations. From a mathematical point of view, the diffusion approximation requires an increasing number of jumps of the underlying Poisson process. From a biological perspective, empirical evidence from brain recordings indicates that concepts are often encoded by coordinated groups of neurons, commonly referred to as neuronal assemblies (Singer et al. 1997; Gerstein et al. 1989). Accordingly, it is reasonable to model a given feature as being represented by multiple neurons and it seems natural that the transition between the neural scale (Poisson counter model) and behavioral scale (drift diffusion model) occurs through an increasing number of neurons. The assumption that these copies are independent is introduced solely for mathematical tractability.

The following theorem is established by means of a coupling between this model and the drift diffusion model with drift $\mu ^j_o = n\gamma ^j_o$.

#### Theorem 2.6

Let $m\in \mathbb {N}^*$, $o\in \mathcal {O}$ the nature of the $m^{th}$ object and $n\in \mathbb {N}^*$. Let $\Pi ^n_m = (\Pi ^{j,n}_m)_{j\in J}$ a family of mutually independent Poisson processes with intensities $(n\gamma ^j_o)_{j\in J}$. Suppose the vector $\gamma _o$ verifies $\gamma ^j_o >0$ for every j∈J. Then there exist absolute constants a,b,c>0 and a |J|-dimensional drifted Brownian motion $W^n_m = (W^{j,n}_m)_{j\in J}$ defined by (1) such that for every x>0,

8 $$\begin{aligned} \mathbb {P}\left( \sup _{j\in J, t\ge 0} \frac{|\Pi ^{j,n}_{m,t}-W^{j,n}_{m,t}|}{\log (n\gamma ^j_o t \vee 1+1)} \ge a+x\right) \le b|J | e^{-c x}. \end{aligned}$$

For j∈J, let us define the hitting times $\tau _n(\Pi ^j):=\inf \left\{ u\ge 0; \Pi ^{j,n}_{m,u} \ge n\theta \right\}$ and $\tau _n(W^j):=\inf \left\{ u\ge 0; W^{j,n}_{m,u} \ge n\theta \right\}$. Let $\alpha \in (0,1)$ and θ>0. Consequently, there exist absolute positive constants *d* and *e* and positive constants $A_{\theta , \gamma _{\text {min}}}$, $B_{\gamma _{\text {min}}}$, $C_{\gamma _{\text {min}}}$ and $D_{\theta ,\gamma _{\text {min}},\Vert \gamma \Vert _\infty }$ depending only on the index parameters such that if $n\ge A_{\theta , \gamma _{\text {min}}} \log \left( |J |\alpha ^{-1}\right)$, then with probability more than 1-α, all the following inequalities hold jointly:

1. The processes $\Pi ^n_m$ and $W^n_m$ verify
  $$\begin{aligned} \sup _{j\in J,t\ge 0} \frac{|\Pi ^{j,n}_{m,t}-W^{j,n}_{m,t}|}{\log (n\gamma ^j_o t \vee 1+1)} \le d +e\log (|J |\alpha ^{-1}). \end{aligned}$$
2. The hitting times $(\tau _n(\Pi ^j))_{j\in J}$ verify
  $$\begin{aligned} \sup _{j\in J} \left\lvert \tau _n(\Pi ^j)- \frac{\theta }{\gamma ^j_o} \right\rvert \le B_{\gamma _{\text {min}}} \sqrt{\frac{\log (|J |\alpha ^{-1})}{n}}. \end{aligned}$$
3. The hitting times $(\tau _n(W^j))_{j\in J}$ verify
  $$\begin{aligned} \sup _{j\in J}\left\lvert \tau _n(W^j)- \frac{\theta }{\gamma ^j_o} \right\rvert \le C_{\gamma _{\text {min}}} \sqrt{\frac{\log (|J |\alpha ^{-1})}{n}}. \end{aligned}$$
4. The difference between the hitting times $(\tau _n(\Pi ^j))_{j\in J}$ and $(\tau _n(W^j))_{j\in J}$ verifies
  $$\begin{aligned} \sup _{j\in J}|\tau _n(\Pi ^j)- \tau _n(W^j) | \le D_{\theta ,\gamma _{\text {min}},\Vert \gamma \Vert _\infty } \log \left( |J |\alpha ^{-1}\right) \frac{\log (n)}{n} . \end{aligned}$$

See Appendix C.4 for more precise version of this result.

The proof of the theorem, provided in Appendix C.4, relies on a coupling result between Poisson and Brownian processes established by Ethier and Kurtz (1986), concentration inequalities, and a novel inequality for Brownian processes that we derived.

With this theorem, we establish a coupling between the Poisson counter model and the drift diffusion model by bounding the supremum of their trajectories on all $\mathbb {R}_+$. Since $\mathbb {E}[\Pi ^{j,n}_{m,t}] = \mathbb {E}[W^{j,n}_{m,t}] = n \gamma ^j_o t$ are growing linearly with *n*, the bound in O(log(n)) of (8) is very strong. Note that the theorem would still hold for dependent processes $\Pi ^n_m = (\Pi ^{j,n}_m)_{j\in J}$ but the coupled processes $W^n_m=(W^{j,n}_m)_{j\in J}$ would have inherited from this dependence. Note also that the assumption $\gamma ^j_o >0$ for every j∈J is easy to satisfy: since the processes with intensity $\gamma ^j_o = 0$ stay equal to zero and never reach θ, we can consider the set of processes with positive intensities instead of *J*.

Besides, in this framework where the threshold nθ is linear in *n*, we establish for every *j* the convergence of the hitting times of both Poisson process $(\Pi ^{j,n}_{m,t})_{t\ge 0}$ and Brownian process $(W^{j,n}_{m,t})_{t\ge 0}$ to $\frac{\theta }{\gamma ^j_o}$, which is the hitting time of the deterministic processes $(\mathbb {E}[\Pi ^{j,n}_{m,t}])_{t\ge 0}$ and $(\mathbb {E}[W^{j,n}_{m,t}])_{t\ge 0}$, with rate in $n^{-1/2}$. Furthermore, we prove that the difference between these two hitting times decreases faster than their convergence rate. This ensures that for large *n*, both models select the same category $\hat{\jmath }$ (the one with larger $\gamma ^j_o$), and we can apply the same bound to the difference between the reaction times of the two models: we have

$$\begin{aligned} \left\lvert \tau (\Pi ) - \tau (W) \right\rvert = \left\lvert \tau _n(\Pi ^{\hat{\jmath }}) \wedge T - \tau _n(W^{\hat{\jmath }}) \wedge T \right\rvert = O\left( \frac{\log (n)}{n}\right) \end{aligned}$$

with high probability. This result explains in particular how reaction times that are modeled by Poisson counter processes, and which are more biologically relevant, might exhibit a behavior like the DDM and have therefore similar adequation to real data.

## Hawkes counter model

### Framework

In this section, we want to model a different decision-making task: the participant does not have prior knowledge about the categories anymore. Similarly as before, the participant has to categorize objects having natures belonging to a set $\mathcal {O}$ into one of several categories belonging to a set *J*. The participant sees the objects sequentially and is given the true answer after each categorization, which allows for learning the categories. Therefore, unlike in the previous task, after each presentation of an object, the participant knows better the categories: the average strength of the evidence accumulation in favor of the categories will change when presented with a new object. The total number of presented objects which allow the participant to learn the categories is denoted by *M*, and the sequential presentation of these *M* objects is called a learning phase. We suppose that at the end of the learning phase, the participant has learned to recognize the object categories, and the average strength of the evidence accumulation in favor of the categories will not change anymore.

### The model

We model this new experiment by a spiking neural network inspired by a previous work in discrete time and without reaction times (Jaffard et al. 2024). During the presentation of the $m^{th}$ object, the accumulation of evidence of the categories j∈J is modeled by a |J|-dimensional Hawkes process $N_m = (N^j_m)_{j\in J}$ with conditional intensity $\lambda _{m,t} = (\lambda ^j_{m,t})_{j\in J}$ defined below. This multivariate process represents the output nodes activity of a spiking neural network.

The $m^{th}$ object is presented for a duration of at least $T_{\text {min}}$ and until either the spike count of one of the processes of $N_m=(N^j_m)_{j\in J}$ reaches the threshold θ, or the maximum allowed duration $T > T_{\text {min}}$ is reached. This minimal duration $T_{\text {min}}$, which was not present in the previous sections, is here to ensure that the network learns the task and that its answers remain accurate once learning is finished. For j∈J, We denote

9 $$\begin{aligned} \tau (N^j) :=\inf \{u\ge 0; N^j_{m,u}\ge \theta \}. \end{aligned}$$

The winning process is then

10 $$\begin{aligned} \hat{\jmath }=\arg \min _{j\in J} \tau (N^j), \end{aligned}$$

and if $\tau (N^{\hat{\jmath }}) <T$ then object *m* is categorized in category $\hat{\jmath }$. However, if $\tau (N^{\hat{\jmath }}) \ge T$ it means that accumulation evidence is not sufficient to reach a decision and the model does not categorize the object. The corresponding reaction time of the whole system is $\tau (N) :=(T_{\text {min}}\vee \tau (N^{\hat{\jmath }}))\wedge T$. Similarly as for the drift diffusion and Poisson counter models, the processes are indexed by time t∈[0,T] which is set back to 0 at each new object.

**Fig. 3:**
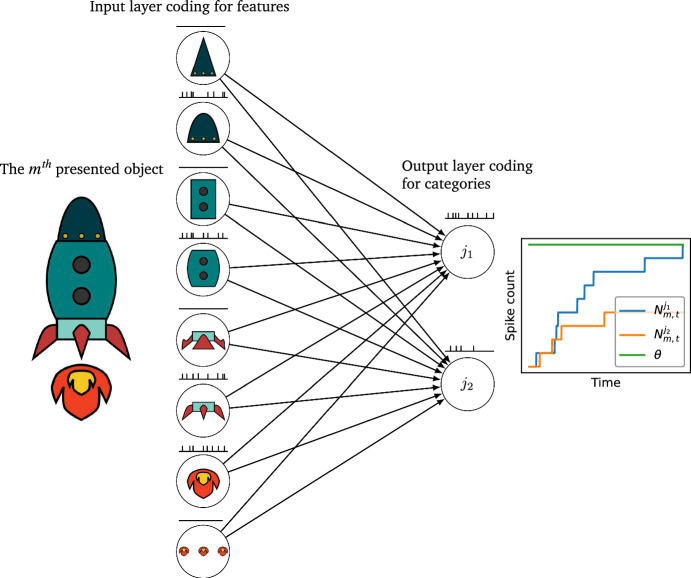
Illustrative example of the network used to model the experiment described in section 4. The task is to classify rockets into two categories, $j_1$ and $j_2$. The input neurons code for different shapes of head, body, fins and flames. The presented object excites the input neurons coding for its features, whereas other input neurons stay silent. The output neurons start spiking with a conditional intensity depending on their connection with the input neurons. The spike train of each neuron is represented on top of it. The first spike count to reach the threshold θ is the one of neuron $j_1$: therefore, the rocket is categorized in category $j_1$

An illustration of the network is given on Figure 3 for the case of the experiment described in Section 4. The input layer codes for features describing the object natures. The set of input neurons is denoted by *I*, and an element i∈I denotes both an input neuron and the feature it is encoding. For instance, in the experiment of Section 4, the objects are rockets and the features are the shape of the head, body and fins and the number of flames. The output layer codes for the categories in which the objects are classified: the activity of the output neurons represents the evidence accumulation in favor of each category. Therefore, the set of output neurons is denoted by *J*, and an element j∈J denotes both an output neuron and the category it is coding for.

**Input neurons activity.** During the presentation of the $m^{th}$ object, the input neuron i∈I starts spiking as a homogeneous Poisson process $(\Pi ^i_{m,t})_{t\ge 0}$ with intensity $\gamma ^i_o \ge 0$ depending only on the nature *o* of the presented object and representing how pronounced feature *i* is in object nature *o*. For instance, in the modeling of the experiment described in Section 4, we use a binary code: either the rocket has feature *i* and the input neuron *i* starts spiking with a strictly positive fixed intensity, either the object does not have the feature and the corresponding input neuron *i* stays silent (*i.e., *its intensity is equal to 0). We assume that the processes $((\Pi ^i_{m,t})_{t\ge 0})_{i\in I, 1\le m \le M}$ are mutually independent.

**Output neurons activity.** During the presentation of the $m^{th}$ object, evidence accumulation in favor of category *j* is modeled by the activity of the output neuron j∈J, which starts spiking as a Hawkes process $(N^j_{m,t})_{t\ge 0}$, which, in contrast to the Poisson process, allows to take into account interactions with presynaptic neurons. Its conditional intensity at time *t* of the presentation of the object is given by:

$$\begin{aligned} \lambda ^j_{m,t} :=\sum _{i\in I} \int _0^t w^{i\rightarrow j}_m g(t-s) d\Pi ^i_{m,s} \end{aligned}$$

where $g\in L^1(\mathbb {R}_+)$ is a non negative function and $w^{i\rightarrow j}_m \ge 0$ is the synaptic weight between input neuron *i* and output neuron *j*: it represents the strength of their connection during the presentation of the $m^{th}$ object. We denote by $\Vert g \Vert _1:=\int _0^\infty g(s)ds$ the norm of *g*. The weights $w^j_m :=(w^{i\rightarrow j}_m)_{i\in I}$ are in the simplex: for every i∈I, $w^{i\rightarrow j}_m \ge 0$ and $\sum _{i\in I} w^{i\rightarrow j}_m = 1$, and they may be seen as a probability distribution over the set of presynaptic neurons of *j*. We denote by $w_m:=(w^j_m)_{j\in J}$ the total weight family. Note that here, we are in the special case of a multivariate Hawkes process without self-interactions (but with mutual interactions).

**Learning rule.** The network learns by updating its synaptic weights after each presented object by using an expert aggregation algorithm (Cesa-Bianchi and Lugosi 2006) thanks to this local paradigm: each presynaptic neuron *i* can be seen as an expert and the strength of the connection $w^{i\rightarrow j}_m$ between *i* and the postsynaptic neuron *j* varies based on gains derived from these connections. After the presentation of the $m^{th}$ object, the postsynaptic neuron *j* attributes the following gain to the presynaptic neuron *i*:

$$\begin{aligned} g^{i\rightarrow j}_m = \left\{ \begin{array}{lll} \widehat{\gamma ^i_m} \times \frac{M}{M^j} & \text {if } o \in j \\ \\ - \widehat{\gamma ^i_m} \!\times \! \frac{M}{M^{j'}} \!\times \! \frac{1}{|J |-1} & \text {if } o \in j'\ne j \end{array} \right. \end{aligned}$$

where *o* is the nature of the presented object, $M^j$ is the total number of presented objects belonging to category *j* during the learning phase and

11 $$\begin{aligned} \widehat{\gamma ^i_m} :=\Pi ^i_{m,T_{\text {min}}} / T_{\text {min}} \end{aligned}$$

is the empirical firing rate of input neuron *i* during duration $$T_{\text {min}}$$. This gain can be interpreted as follows: if the presented object belongs to category *j*, neuron *j* should spike frequently to be the first to reach the threshold θ. Therefore, it assigns positive gains to its presynaptic neurons, with these gain being proportional to their firing rates, thereby strengthening its connections with the input neurons that encode the most relevant features describing category *j*. Conversely, if the presented object does not belong to category *j*, neuron *j* should spike less to avoid reaching the threshold θ before the neuron coding for the object’s true category. In this case, it assigns negative gains (*i.e., *losses) to its presynaptic neurons in order to reduce the strength of its connections with the most active neurons when presented with objects it should not encode. Note that neuron *j* does not need to know what the other neurons $j^{\prime }$ are doing to attribute the gains. Also note that the gain depends on the ratio $$M/M_j$$, that is roughly speaking the proportion of objects in each category, and the larger $$T_{\text {min}}$$, the better the estimation of the input neurons firing rates.

We denote by $G^{i\rightarrow j}_m :=\sum _{m'=1}^m g^{i\rightarrow j}_{m'}$ the cumulated gain of input neuron *i* w.r.t. output neuron *j* until the $m^{th}$ object. Then after the presentation of the object, the weights of neuron *j* are updated using the expert aggregation EWA (Exponentially Weighted Average) (Cesa-Bianchi and Lugosi 2006):

12 $$\begin{aligned} w^{i\rightarrow j}_{m+1} :=\frac{\exp (\eta G^{i\rightarrow j}_m)}{\sum _{l\in I} \exp (\eta G^{l\rightarrow j}_m)} \end{aligned}$$

where the parameter η>0 is called the learning rate and determines how fast the network learns. In what follows, we choose

13 $$\begin{aligned} \eta = \frac{\eta _0}{\sqrt{M}} \text{ where } \eta _0>0. \end{aligned}$$

Note that this learning rule is local: the gains used by neuron *j* and therefore the update of the synaptic weights depend only on the activity of its presynaptic neurons.

We denote by $\mathcal {F}_m$ the σ-algebra generated by every event which happened until the end of the presentation of the $m^{th}$ object and $\mathcal {F}_0$ the trivial σ-algebra. Note that the weights $w^{i\rightarrow j}_{m+1}$ are $\mathcal {F}_m$-measurable.

**Additions over the previous model.** This new version differs from the model that we proposed in Jaffard et al. (2024) by two main features. In this previous work, the neurons activities were defined in discrete time, and every object was presented during a fixed duration: the presented object was classified in the category of the output neuron with the highest spike count at the end of the presentation. Here, the neurons activities are defined in continuous time and the objects are presented for dynamic durations. To model behavior, we introduced a threshold and a limit duration, which allowed to model participant reaction times and the possibility that the participant will not decide between the different categories if not enough evidence has been accumulated.

**Comparison with the Poisson counter model and interpretation.** Similarly to the Poisson counter model, the evidence accumulation in favor of the different categories are interpreted as spike counts of neurons coding for these categories. However, unlike the Poisson counter model, the intensities of the output neurons are not given arbitrarily as prior knowledge about the categories, but are learned with a local rule as the network sees new objects. Our model can have the following interpretation: the input layer is a Poisson counter model which can recognize simple features, and an output layer is added and trained to recognize more complex concepts. This is biologically relevant since in the brain, elementary concepts can be depicted by the responses of individual neurons, whereas more intricate ones are represented by groups of interconnected neurons that work together (Singer et al. 1997).

### Mathematical analysis

In this section, we establish theoretical guarantees for our model. We begin by analyzing the asymptotic behavior of the network weights, which provides insights into the network behavior after the learning phase. Next, we outline the conditions necessary for the network to accurately categorize new objects following the learning process. This successful performance stems not from prior knowledge of the categories but from the learning phase itself. Finally, we demonstrate that the drift diffusion model can be derived from our model, establishing that it can be approximated by complex networks of interacting neurons having a learning mechanism.

#### Asymptotic behavior

Let us study the network’s asymptotic behavior. We introduce a notation: given a set *E* and a quantity $x_e \in \mathbb {R}$ indexed by e∈E, we denote its mean by $\langle x_e \rangle _{e\in E} :=\frac{1}{|E |}\sum _{e\in E} x_e$.

##### Definition 3.1

*(Feature discrepancy)* Let i∈I and j∈J. The feature discrepancy of input neuron *i* w.r.t. output neuron *j* is

$$\begin{aligned} d^{i\rightarrow j} :=\langle \gamma ^i_o \rangle _{o\in j} - \langle \langle \gamma ^i_o \rangle _{o\in j'} \rangle _{j'\in J\setminus \{j\}}. \end{aligned}$$

The feature discrepancy of input neuron *i* w.r.t. output neuron *j* measures how sensible neuron *i* is to category *j* by comparing its average firing rate when presented with objects of category *j* to its average firing rate when presented with objects of other categories. We can now define the set of input neurons which are the most sensible to category *j* by $I^j :=\arg \max _{i\in I} d^{i\rightarrow j}$, as well as the gap in discrepancy when $I^j \subsetneq I$

$$\begin{aligned} \delta ^j :=\max _{i\in I} d^{i\rightarrow j} - \max _{i\in I\setminus I^j} d^{i\rightarrow j} \end{aligned}$$

which measures how sensible the best input neurons are with respect to the others.

##### Assumption 3.2

During the learning phase, each nature *o* has been presented the same amount of times to the network, *i.e.,* $\frac{M}{|\mathcal {O} |}$ times.

##### Proposition 3.3

Let $\alpha \in (0,1)$ and $\gamma _{\text {min}} :=\min _{o\in \mathcal {O}, i\in I}\{\gamma ^i_o; \gamma ^i_o>0\}$. Suppose Assumption 3.2 holds. For j∈J, let $w^j_\infty = (w^{i\rightarrow j}_\infty )_{i\in I}$ the weight family such that $w^{i\rightarrow j}_\infty :=\frac{1}{|I^j |}1\!\!1_{\{i\in I^j\}}$. Then there exist positive constants $A_{|\mathcal {O} |, \gamma _{\text {min}}}$ and $B_{|\mathcal {O} |, \eta _0, \Vert \gamma \Vert _\infty }$ depending only on the index parameters such that if

$$\begin{aligned} M T_{\text {min}} \ge A_{|\mathcal {O} |, \gamma _{\text {min}}} \log (|I ||J |\alpha ^{-1}) \end{aligned}$$

then with probability 1-α, at the end of the learning phase, the synaptic weights verify for all j∈J

$$\begin{aligned}&\left\Vert w^j_{M+1} - w^j_\infty \right\Vert _2 \le B_{|\mathcal {O} |, \eta _0, \Vert \gamma \Vert _\infty } \\&\quad \sqrt{\frac{|I |}{T_{\text {min}}} \log (|I ||J |\alpha ^{-1})}+ 1\!\!1_{\{I^j \subsetneq I\}} |I |^{3/2} \exp (- \eta _0\delta ^j \sqrt{M}), \end{aligned}$$

where $\eta _0$ is defined by (13).

See Appendix 1 for a version of this result with exact constants.

In Proposition 3.3, we prove that the network weights converge to a limit family $w_\infty :=(w^{i\rightarrow j}_\infty )_{i\in I, j\in J}$ and we provide rates of convergence. The error is twofold. The first part, in $O(\frac{1}{\sqrt{T_{\text {min}}}})$ where $T_{\text {min}}$ is the minimum duration for which an object is presented, comes from the randomness of the system and is derived using concentration inequalities to control how good is the estimation of $\gamma ^i_o$ by $\widehat{\gamma ^i_m}$ (11). The second part, in $\exp (O(-\delta ^j \sqrt{M}))$ where *M* is the number of objects presented during the learning phase, comes from the use of the expert aggregation algorithm EWA: the larger the gap in discrepancy, the faster the convergence. This limit family has the following interpretation: at the limit, output neuron *j* is connected only and with equal strength to input neurons which are the most sensible to category *j*.

Note that this asymptotic analysis is feasible because the weight-update rule has a simple form. To our knowledge, no comparable results exist for learning rules such as Spike-timing-dependent plasticity (STDP), for which the update formulas are more complex.

This result can be compared to Theorem 3.4 of Jaffard et al. (2024) which describes the asymptotic behavior of the network in discrete time with fixed object presentation durations. The present error has the same order of magnitude, where the number of time steps of each presented objects is replaced by the minimum duration of each object presentation $T_{\text {min}}$.

#### Theoretical guarantees

At the limit (*M* and $T_{min}$ tend to infinity), the network’s weights are the deterministic family $w_\infty$ given by Proposition 3.3. For $o\in \mathcal {O}$, let

$$\begin{aligned} \bar{\lambda }^j_{\infty , o} :=\sum _{i\in I} w^{i\rightarrow j}_\infty \gamma ^i_o \end{aligned}$$

which is the asymptotic average firing rate of neuron *j* when objects with nature *o* are presented. Therefore, the average number of spikes of neuron *j* on [0, *t*] with weights $w^j_\infty$ when presented with an object of nature *o* is

$$\begin{aligned} \bar{\lambda }^j_{\infty , o} \mathbb {G}(t) \end{aligned}$$

where $\mathbb {G} : t \mapsto \int _0^t \int _0^s g(s-u)du ds$. Hence, on average, the first neuron whose spike count reaches threshold θ is the one with highest $\bar{\lambda }^j_{\infty , o}$. This leads to the following new notion of margin. Let ${{\boldsymbol{\Delta }}_\lambda }>0$.

##### Assumption 3.4

*(Margin(*${{\boldsymbol{\Delta }}_\lambda }$*))* For every category $j^*\in J$, for every $j\ne j^*$, for every object nature $o\in j^*$ we have

$$\begin{aligned} \bar{\lambda }^{j^*}_{\infty , o} > \bar{\lambda }^j_{\infty , o} + {{\boldsymbol{\Delta }}_\lambda }. \end{aligned}$$

It means that at least in the limit the same gap as the ones for DDM and Poisson counter model holds. Since we explicitly know the limit family $w_\infty$, this assumption can be interpreted as a condition on the categories j∈J and the features represented by the input neurons i∈I. This condition holds when the categories can be described as combinations of relevant features. In Section 4.4, we prove that this assumption is satisfied in the task performed by the participants of the experiment. For a detailed characterization of the categories learnable by the discrete time network, we refer the reader to Section 4.2.4 of Jaffard et al. (2026), where we describe the categories learnable by an extended version of our network that includes hidden layers, but no reaction time.

This assumption can be related to Assumption 2.2 (Margin(${\boldsymbol{\Delta }}_{\mu }$)) on the drift of the DDM and Assumption 2.4 (Margin(${{\boldsymbol{\Delta }}_\gamma }$)) on the intensities of the Poisson counter model. Indeed, similarly as in the theoretical studies of these two models, we need an assumption on the average evidence accumulation in favor of the several categories. However, unlike for the DDM and the Poisson counter model, this assumption does not reflect prior knowledge on the categories, but the ability of our network to code for the categories. It is only after the network has learned that it reaches these firing rates.

Under these assumptions, we can formulate the following result.

##### Theorem 3.5

Let $\alpha \in (0,1)$ and $\eta _0>0$, and $\mathbb {G} : t \mapsto \int _0^t \int _0^s g(s-u)du ds$. Let ${{\boldsymbol{\Delta }}_\lambda }>0$ and suppose Assumptions 3.2 and 3.4 (Margin (${{\boldsymbol{\Delta }}_\lambda }$)) hold. There exist positive constants $A_{\eta _0, \delta _{\text {min}}, \Vert \gamma \Vert _\infty , {{\boldsymbol{\Delta }}_\lambda }}, B_{\eta _0, \Vert \gamma \Vert _\infty , {{\boldsymbol{\Delta }}_\lambda }, |\mathcal {O} |}, C_{\Vert \gamma \Vert _\infty ,{{\boldsymbol{\Delta }}_\lambda }, \Vert g \Vert _1},D_{\Vert \gamma \Vert _\infty , {{\boldsymbol{\Delta }}_\lambda }, \Vert g \Vert _1}, E_{{{\boldsymbol{\Delta }}_\lambda }}$ and $F_{\Vert \gamma \Vert _\infty , \Vert g \Vert _1}$ depending only on the index parameters such that if the parameters *M*, $T_{\text {min}}$, *T*, and θ verify

14 $$\begin{aligned} M\ge A_{\eta _0, \delta _{\text {min}}, \Vert \gamma \Vert _\infty , {{\boldsymbol{\Delta }}_\lambda }} \log (|I |)^2 \end{aligned}$$

where $$\delta _{\text {min}} :=\min _{j\in J} \delta ^j$$,

15 $$\begin{aligned} T_{\text {min}} \ge B_{\eta _0, \Vert \gamma \Vert _\infty , {{\boldsymbol{\Delta }}_\lambda }, |\mathcal {O} |} |I |^2 \log \left( |I ||J |\alpha ^{-1}\right) \end{aligned}$$

16 $$\begin{aligned} \mathbb {G}(T) > C_{\Vert \gamma \Vert _\infty ,{{\boldsymbol{\Delta }}_\lambda }, \Vert g \Vert _1} \log (|J |\alpha ^{-1}) \end{aligned}$$

17 $$\begin{aligned} \theta \ge D_{\Vert \gamma \Vert _\infty , {{\boldsymbol{\Delta }}_\lambda }, \Vert g \Vert _1} \left( \log (|J |\alpha ^{-1}) \vee \mathbb {G}(T_{\text {min}}) \right) \end{aligned}$$

and

18 $$\begin{aligned} \theta < E_{{{\boldsymbol{\Delta }}_\lambda }} \mathbb {G}(T) - F_{\Vert \gamma \Vert _\infty , \Vert g \Vert _1} \left( \sqrt{ \mathbb {G}(T)\log \left( |J |\alpha ^{-1}\right) } + \log \left( |J |\alpha ^{-1}\right) \right) , \end{aligned}$$

then with probability larger than 1-α, we have that for all nature $o\in \mathcal {O}$ of the object presented after the learning phase, that is the M+1 object, if we denote by $j^*$ its category, the choice $\hat{\jmath }$ (10) and the reaction time $\tau (N^{\hat{\jmath }})$ (9) satisfy

$$\begin{aligned} \hat{\jmath }= j^* \quad \text{ and } \quad \tau (N^{\hat{\jmath }}) < T. \end{aligned}$$

See Appendix C.6 for a more precise version of this result.

This theorem provides conditions on the minimal duration $T_{\text {min}}$, maximal duration *T*, number of presented objects during the learning phase *M* and threshold θ required for the model to correctly categorize objects after learning. Let us start by commenting on these conditions.

1. **Sufficient learning phase duration:** The network effectively learns the categories if *M* verifies (14), ensuring a long enough learning phase.
2. **Sufficient minimal observation time for learning objects:** The network has sufficient time to infer the input neurons firing rate to update its synaptic weights if $T_{\text {min}}$ verifies (15).
3. **Sufficient maximal observation time:** The network has sufficient time to detect differences in spike counts between the true category process $N^{j^*}_{M+1}$ and others if *T* verifies (16).
4. **Sufficient threshold:** The spike count difference between the true category process $N^{j^*}_{M+1}$ and others is significant at decision time if θ verifies (17).
5. **Sufficiently low threshold:** The network has a sufficiently low threshold to be able to take a decision before limit duration *T* if θ verifies (18).

Note that we always have $\mathbb {G}(t)\le \Vert g \Vert _1 t$ and if the function *g* has compact support of negligible length with respect to *T*, as it is assumed in the following section, then $\mathbb {G}(T)\approx \Vert g \Vert _1 T$. In this case, the upper bound on θ (18) has the same order of magnitude regarding the limit duration *T* as those in Theorem 2.3 for the DDM and Theorem 2.5 for the Poisson counter model, *i.e., *in *O*(*T*). It is reasonable to make such an assumption because a participant’s reaction time is typically around a few seconds and in practice we chose T=5s in the experiment described in Section 4, whereas the effect of a presynaptic neuron’s spike on a postsynaptic neuron, represented by the support of the function *g*, lasts only a few milliseconds.

The same way as for the constant ${\boldsymbol{\Delta }}_{\mu }$ quantifying the larger drift for the DDM and the constant ${{\boldsymbol{\Delta }}_\gamma }$ quantifying the larger gap in intensity for the Poisson counter model, here, the value of the constants given in Appendix C.6 indicate that the larger ${{\boldsymbol{\Delta }}_\lambda }$, *i.e., *the larger the gap between the limit firing rate of the output neuron coding for the true category and the other output neurons, the larger the size of the thresholds interval enabling a correct categorization with high probability. Similarly as in Theorem 2.3, we need the maximal duration *T* to be sufficiently large (16) and unlike in Theorems 2.3 and 2.5 where there was no learning mechanism, additional assumptions are needed here regarding $T_{\text {min}}$ and *M* to ensure a correct learning. Note that all these assumptions become weaker as ${{\boldsymbol{\Delta }}_\lambda }$ increases.

The proof, detailed in Appendix C.6, relies on several key components. First, concentration inequalities are employed to prove that the processes $(N^j_{M+1,t})_{t\ge 0}$ are closely approximated by their compensators. Proposition 3.3 is then used to show that these compensators are close to the average spike counts with limit weights. Finally, Assumption 3.4 is applied to derive conditions under which the network correctly categorizes the new object.

### Coupling between the Drift Diffusion Model and the Hawkes Counter Model

Similarly as for the Poisson counter model (Section 2.4), we can build a coupling between the processes given by the drift diffusion model and the Hawkes counter model under a certain framework. Here, we work conditionally to what happened until object *m*, whether it is during learning or post learning: the synaptic weights $w^{i\rightarrow j}_m$ are therefore assumed to be deterministic. For the same reasons as in Section 2.4, we suppose that we dispose of *n* independent copies of the network, and each independent network is governed by the same weights $(w^{i\rightarrow j}_m)_{i\in I,j\in J}$. During the presentation of object *m*, for each feature *i*, there are *n* input neurons spiking as a Poisson processes with intensity $\gamma ^i_o$ where *o* is the nature of object *m*: we denote by $(\Pi ^{i,n}_{m,t})_{t\ge 0}$ the sum of their count, which is a Poisson process with intensity $n\gamma ^i_o$ by independence. In the same way, there are *n* output neurons coding for the same category j∈J. The evidence in favor of category *j* is then the sum of their counts and is denoted $(N^{j,n}_{m,t})_{t\ge 0}$, which is a Hawkes process with intensity

19 $$\begin{aligned} \lambda ^{j,n}_{m,t} = \sum _{i\in I} \int _0^t w^{i\rightarrow j}_m g(t-s) d\Pi ^{i,n}_{m,s}. \end{aligned}$$

Note that two other frameworks lead to the same processes: only one network of size |I|×|J| with input neurons intensities $(n\gamma ^i_o)_{i\in I,o\in \mathcal {O}}$, but also one network with n×|I| input neurons and only |J| output neurons.

During the presentation of the $m^{th}$ object, the average evidence accumulation in favor of category *j* at present time *t* of object *m* is then

$$\begin{aligned} \mathbb {E}[N^{j,n}_{m,t}] = n\bar{\lambda }^j_m \mathbb {G}(t) \end{aligned}$$

where $\bar{\lambda }^j_m :=\sum _{i\in I} w^{i\rightarrow j}_m \gamma ^i_o$ with *o* the nature of the presented object.

#### Theorem 3.6

Let $\alpha \in (0,1)$, $m\in \mathbb {N}^*$, $n\in \mathbb {N}^*$ and $o\in \mathcal {O}$ the nature of the $m^{th}$ object. Let $N^n_m = (N^{j,n}_m)_{j\in J}$ be a |J|-dimensional Hawkes process with intensity function defined by (19) and let $\bar{\lambda }_{\text {min}}:=\min _{j\in J} \bar{\lambda }^j_m$. We assume that the function *g* has compact support included in $[0,n^{-1/2}]$, that its norm $\Vert g \Vert _1$ does not depend on *n*, and that $\bar{\lambda }_{\text {min}}>0$. Then there exist positive constants $A_{\Vert \gamma \Vert _\infty ,\Vert g \Vert _1, \bar{\lambda }_{\text {min}},\theta }, B_{\Vert g \Vert _1,\Vert \gamma \Vert _\infty }, C_{\Vert \gamma \Vert _\infty ,\Vert g \Vert _1, \bar{\lambda }_{\text {min}},\theta }, D_{\Vert \gamma \Vert _\infty ,\Vert g \Vert _1, \bar{\lambda }_{\text {min}},\theta }$ and $E_{\Vert \gamma \Vert _\infty ,\Vert g \Vert _1, \bar{\lambda }_{\text {min}},\theta }$ depending only on the index parameters such that if $n\ge A_{\Vert \gamma \Vert _\infty ,\Vert g \Vert _1, \bar{\lambda }_{\text {min}},\theta } \log \left( (|I |+|J |)\alpha ^{-1}\right) ^{3/2}$, then there exists a |J|-dimensional drifted Brownian motion with correlated noise $W_m^n = (W^{j,n}_m)_{j\in J}$ with mean vector defined by $\forall j\in J, \forall t\ge 0, \quad \mathbb {E}[W^{j,n}_{m,t}] = n\bar{\lambda }^j_m\mathbb {G}(t)$, and covariance matrix defined by $\forall j_1,j_2\in J, \forall t,s\ge 0$,

$$\begin{aligned} \mathop {\text {Cov}}(W^{j_1,n}_{m,t}, W^{j_2,n}_{m,s}) = n (t\wedge s) \left( \sum _{i\in I}w^{i\rightarrow j_1}_m w^{i\rightarrow j_2}_m \gamma ^i_o + 1\!\!1_{j_1 = j_2} \Vert g \Vert _1 \bar{\lambda }^{j_1}_m \right) \end{aligned}$$

such that with probability more than 1-α, all the following inequalities hold jointly.

1. The processes $$\Pi ^n_m$$$$N^n_m$$ and $$W^n_m$$ verify
  20 $$\begin{aligned} \sup _{j\in J, t> 0}\frac{ \left\lvert N^{j,n}_{m,t} - W^{j,n}_{m,t} \right\rvert }{n^{1/4}(t\vee 1)^{1/4}\log (nt + 1)^{3/4}} \le B_{\Vert g \Vert _1,\Vert \gamma \Vert _\infty } \log \left( \frac{|I |+|J |}{\alpha }\right) ^{3/4}. \end{aligned}$$
2. The hitting times $(\tau _n(N^j))_{j\in J}$ defined for j∈J by $\tau _n(N^j):=\inf \{u\ge 0; N^{j,n}_{m,u} \ge n\theta \}$ verify
  $$\begin{aligned} \sup _{j\in J}\left\lvert \tau _n(N^j)- \frac{\theta }{\bar{\lambda }^j_m \Vert g \Vert _1} \right\rvert \le C_{\Vert \gamma \Vert _\infty ,\Vert g \Vert _1, \bar{\lambda }_{\text {min}},\theta } \log \left( \frac{|I |+|J |}{\alpha }\right) ^{3/4} n^{-1/2}. \end{aligned}$$
3. The hitting times $(\tau _n(W^j))_{j\in J}$ defined for j∈J by $\tau _n(W^j):=\inf \{u\ge 0; W^{j,n}_{m,u} \ge n\theta \}$ verify
  $$\begin{aligned} \sup _{j\in J}\left\lvert \tau _n(W^j)- \frac{\theta }{\bar{\lambda }^j_m \Vert g \Vert _1} \right\rvert \le D_{\Vert \gamma \Vert _\infty ,\Vert g \Vert _1, \bar{\lambda }_{\text {min}},\theta } \log \left( |J |\alpha ^{-1}\right) n^{-1/2}. \end{aligned}$$
4. The difference between the hitting times $(\tau _n(N^j))_{j\in J}$ and $(\tau _n(W^j))_{j\in J}$ verifies
  21 $$\begin{aligned} \sup _{j\in J} |\tau _n(N^j)- \tau _n(W^j) |&\le E_{\Vert \gamma \Vert _\infty ,\Vert g \Vert _1, \bar{\lambda }_{\text {min}},\theta } \log \left( \frac{|I |+|J |}{\alpha }\right) ^{3/4} \left( \frac{\log (n)}{n}\right) ^{3/4} . \end{aligned}$$

This theorem, established by means of a coupling between the Hawkes counter model and the drift diffusion model, enables to bound the supremum of their trajectories over all $\mathbb {R}_+$.

The proof, provided in Appendix C.7, builds on the same coupling result between Poisson and Brownian processes established by Ethier and Kurtz (1986), which we previously used to prove Theorem 2.6 and new inequalities that we derived for Brownian and Hawkes processes.

To achieve this, we had to assume that $\bar{\lambda }_{\text {min}}>0$, *i.e., *that $\bar{\lambda }^j_m>0$ for every j∈J. This is easy to satisfy: indeed, if $\bar{\lambda }^j_m =0$, it means that neurons coding for category *j* are not linked to any input neuron currently active, therefore we are in the non interesting case where the process $N^{j,n}_{m}$ stays equal to zero and never reach nθ. We can then consider the set of processes such that $\bar{\lambda }^j_m>0$ instead of *J* with no loss of generality. We also had to assume that *g* has compact support decreasing at a rate of $n^{-1/2}$. This implies that the number of spikes influencing a group of *n* neurons encoding the same category *j* does not scale linearly with *n*, as it would if *g* had a fixed support, but rather grows at a rate of $n^{1/2}$. This assumption further ensures that $\mathbb {G}(t) \approx \Vert g \Vert _1 t$, meaning the mean vector of the drifted Brownian motion given by the theorem closely resembles that of classical diffusion processes. Unlike in the model described in Section 2.2 and in Theorem 2.6, where we established a strong approximation result between the DDM and the Poisson counter model, the drift $\mu ^j_m :=n\bar{\lambda }^j_m$ of process $W^{j,n}_m$ does not depend only on the nature *o* of the presented object, but also on the presentation number *m* through its dependency in the weights $w^j_m$. The covariance matrix of the process $W^n_m$ is also not diagonal: the processes of $(W^{j,n}_m)_{j\in J}$ are correlated, reflecting the natural dependencies within neural networks. They can be expressed as solutions of the |*J*|-dimensional stochastic differential equation, which is defined for each process *j* as follows:

$$\begin{aligned} dW^{j,n}_{m,t} = n\bar{\lambda }^j_m d\mathbb {G}(t) + \sqrt{n\bar{\lambda }^j_m\Vert g \Vert _1} dB^j_t + \sum _{i\in I} w^{i\rightarrow j}_m \sqrt{n\gamma ^i_o} dB^i_t . \end{aligned}$$

Each $B^j_t$ represents an independent noise source, while the collection $(B^i_t)_{i \in I}$ introduces correlated noise terms across all processes. This correlation arises from the shared influence of the same input neurons on each output neuron in our model. The obtained bound on the trajectories (20) is in $O(n^{1/4} \log (n)^{3/4})$, which is less tight than the O(log(n)) bound established in Theorem 2.6 for the trajectories of the Poisson counter model and the drift diffusion model. This is due to the greater complexity of the Hawkes counter model.

Besides, similarly as in Theorem 2.6 for Poisson and Brownian processes, we establish for every *j* the convergence of the hitting times of both Hawkes process $(N^{j,n}_{m,t})_{t\ge 0}$ and Brownian process $(W^{j,n}_{m,t})_{t\ge 0}$ to $\frac{\theta }{\bar{\lambda }^j_m\Vert g \Vert _1}$, which is the hitting time of the deterministic processes $(\mathbb {E}[N^{j,n}_{m,t}])_{t\ge 0}$ and $(\mathbb {E}[W^{j,n}_{m,t}])_{t\ge 0}$. However, unlike in Theorem 2.6 where the limit hitting time depends on prior knowledge $\gamma ^j_o$, here the limit hitting time depends on $w^j_m$, *i.e., *on the learning stage of the model. Furthermore, we prove that the difference between these two hitting times decreases faster than $n^{-1/2}$, which is an upper bound on their respective convergence rate to their common limit. The resulting bound (21) is less tight than the similar bound of Theorem 2.6 due to the fact that the bound on the trajectories (20) is also less tight in this case.

Therefore, this theorem ensures that both models select the same category $\hat{\jmath }$, and we can apply the same bound to the difference between the reaction times of the two models: we have

$$\begin{aligned} \left\lvert \tau (N) - \tau (W) \right\rvert = \left\lvert (T_{\text {min}}\vee \tau _n(N^{\hat{\jmath }})) \wedge T- (T_{\text {min}}\vee \tau _n(W^{\hat{\jmath }}))\wedge T \right\rvert = O\left( \left( \frac{\log (n)}{n}\right) ^{3/4}\right) \end{aligned}$$

establishing that both models provide similar reaction times and categorizations.

Consequently, this theorem confirms that the dynamics of evidence accumulation for category decisions are similar in both models. Extending beyond the results established in Section 2.4, this finding demonstrates that DDMs can emerge from complex models of interacting spiking neurons with a learning mechanism. This provides new insights into the biological interpretation of DDMs and supports the idea that the prior knowledge assumed in these models can arise through a learning process.

Note that this result is more general than the setting considered in the preceding sections. First, it provides a bound on the difference between the trajectories of the two models over all of $\mathbb {R}_+$, and thus does not rely on either the maximal duration *T* or the minimal duration $T_{\text {min}}$ introduced in the Hawkes counter model. Second, it applies to any deterministic choice of weights $(w^{i\rightarrow j}_m)_{i\in I, j\in J}$; in particular, the weights may arise from a different learning rule, such as STDP.

## The experiment

To validate our model, we designed a categorization task in the form of an online video game, accessible at https://3ia-demos.inria.fr/mel/en/. The goal of the game was for participants to learn which rockets displayed on the screen were capable of reaching the moon and which were not. We opted for a video game format rather than a classical categorization task to make the activity more engaging, particularly for younger participants. The game was coded to allow flexibility in the difficulty of the categorization task. For this experiment, participants were presented with the easiest level (details provided in the Stimuli and Categories Section 4.2).

### Participants

A total of 99 participants, including 45 middle school students and 54 undergraduate students, were recruited for the study. Middle school students (23 male, 22 female) ranged in age from 11 to 15 years (M = 12.13, SD = 1.15), while undergraduate students were all aged between 18 and 25. All participants reported normal or corrected-to-normal vision. The study was approved by the ethics committee of Université Côte d’Azur and was conducted in accordance with relevant guidelines and regulations (ethics approval number 2022-098). All participants provided informed consent prior to participation. Participants were recruited via one of their teachers. Middle school students performed the task at school whereas undergraduate students performed it at home.

### Stimuli and categories

**Fig. 4:**
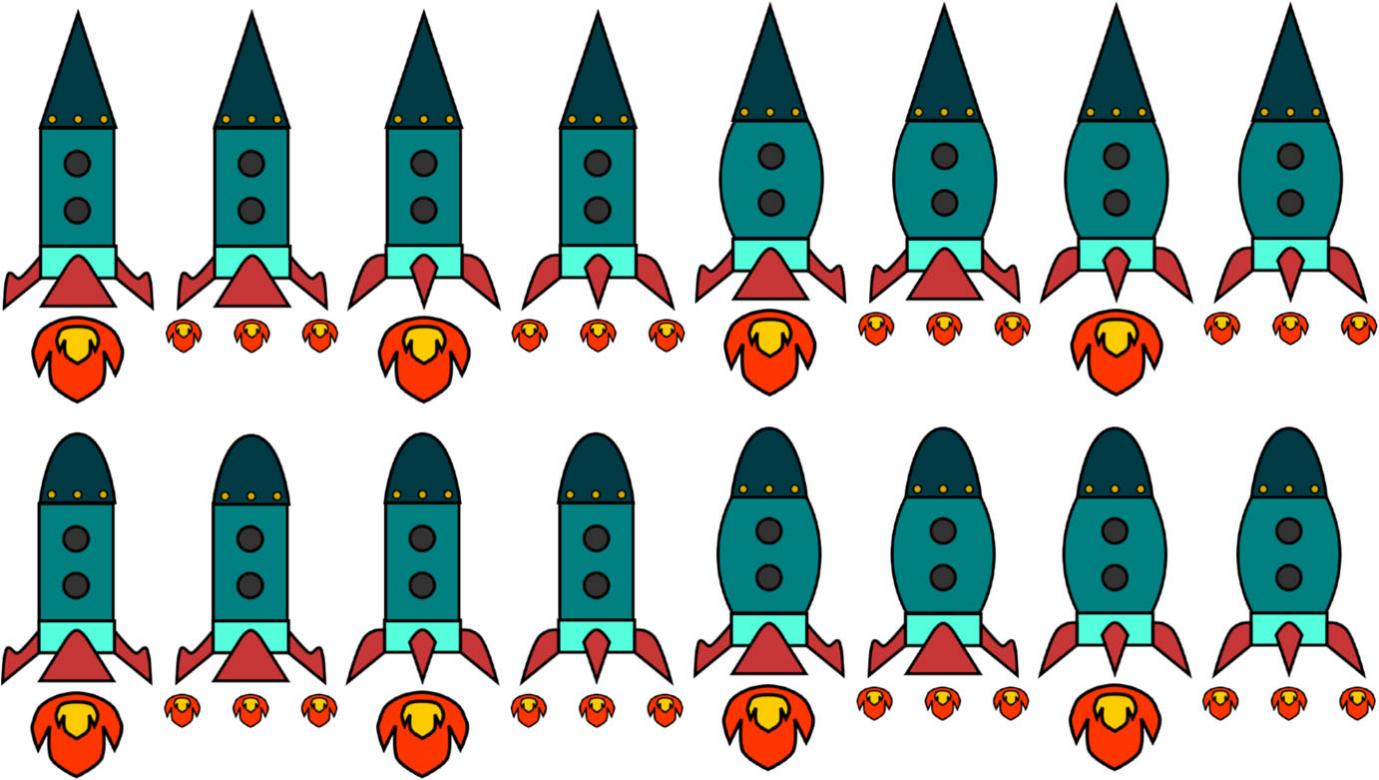
The 16 objects

The set of stimuli consisted of 16 unique rockets, each defined by four characteristics, with two possible features for each: the shape of the head (sharp or round), the shape of the body (straight or round), the shape of the fins (straight or curved), and the number of flames (one or three). These rockets are illustrated in Figure 4. As mentioned above, participants were administered the easiest level of the categorization task. At this difficulty level, the categories were formed based on a single characteristic selected randomly at the beginning of the task. Rockets were divided into two groups: those with one feature of the chosen characteristic and those with the alternative feature. For example, if the selected characteristic was the shape of the head, rockets with a sharp head were categorized as capable of landing on the moon, while rockets with a round head were categorized as unable to land on the moon. Alternatively, rockets with a round head could also have been assigned to the “capable of landing on the moon" category, as the assignment was made randomly. From these two categories, five rockets from each group were randomly selected, resulting in 10 unique rockets presented during the learning phase. The remaining six rockets were used in the transfer phase.

### Procedure

Participants performed the task on a computer. Middle school students were supervised by their teachers, and undergraduate students performed the task at home. Prior to the experiment, participants completed a series of demographic questions and received detailed instructions on how to perform the task. The experiment consisted of two phases: a learning phase and a transfer phase. In the learning phase, participants were asked to determine which rockets were capable of landing on the moon and received immediate feedback on the correctness of their responses. Stimuli were presented one at a time at the center of the screen for 5 seconds. To help participants track time, a clock icon was displayed. After viewing each stimulus, participants made their decision by selecting one of two buttons labeled ‘Yes’ or ‘No’ to indicate whether the rocket could land on the moon. Upon selecting a button, feedback on the correctness of the response was presented for 2 seconds, including a visual cue in the form of a green checkmark for correct responses or a red cross for incorrect ones. Additionally, an animation briefly depicted whether the rocket was able to land on the moon. If participants did not provide a response within 5 seconds, a ‘time is up’ icon appeared on the screen. To reinforce learning, a progression bar was displayed at the top left of the screen, with a green checkmark appearing each time a correct response was given. Participants were required to correctly classify 15 consecutive rockets to pass the learning phase. The 10 unique rockets were presented in a random order and repeated until the participant reached the learning criterion, with a new random order at each repetition. After the learning phase, participants proceeded to the transfer phase, where they were instructed to classify new rockets that had not been presented in the learning phase. Stimuli were presented in the same manner as in the learning phase. In this phase, six new rockets were presented three times in a random order, resulting in a total of 18 trials. No feedback was provided during the transfer phase. Participants could choose to withdraw from the experiment at any time by selecting a cross icon located at the top left of the screen.

### Modeling

We modeled this experiment with the model as described in Figure 3. Object natures are separated between a set of learning natures $\mathcal {O}_l$ containing the 10 learning rockets and a set of transfer natures $\mathcal {O}_{tr}$ containing the 6 transfer rockets. The set of feature *I* contains the 8 possible features of the rockets (two shapes for the head, two shapes for the body, two shapes for the fins and two possible type of flames). The two categories denoted $j_1$ and $j_2$ are characterized by a certain characteristic declined in two features $i_1$ and $i_2\in I$: every object nature *o* has either feature $i_1$ or feature $i_2$, and category $j_1$ contains every nature *o* with $i_1$ whereas category $j_2$ contains every nature *o* with $i_2$.

When an input neuron *i* is presented with a rocket having nature $o\in \mathcal {O}_l\cup \mathcal {O}_{tr}$, it starts spiking with intensity $\gamma ^i_o :=1\!\!1_{\{\text {object o{ hasfeature}i}\}}\gamma$ with γ>0. In other words, input neuron *i* spikes with firing rate γ if the object has feature *i* and stays silent otherwise.

#### Proposition 4.1

For the set of object natures $\mathcal {O}_l$ and input neuron intensities $\gamma ^I$ defined above, the sets defined in Proposition 3.3 are $I^{j_1} = \{i_1\}$ and $I^{j_2} = \{i_2\}$, and the limit family $w_{\infty }$ verifies Assumption 3.4: it enables to categorize well the objects and we can choose any constant in (0,γ) for ${{\boldsymbol{\Delta }}_\lambda }$.

### Analysis

**Fig. 5:**
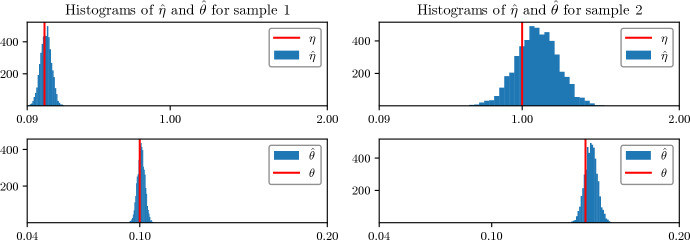
Samples of the posteriors given by the SBI method for two sets of simulated data. The prior used for the parameters tuple $(\eta ,\theta )$ is a uniform distribution on [0.09,2]×[0.04,0.2]. In red, the true values of η and θ used to simulate data. In blue, the histogram of the values $\hat{\eta }$ and $\hat{\theta }$ given by the posteriors given by the SBI method

**Fig. 6:**
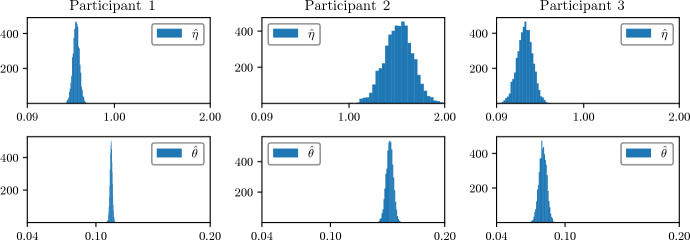
Samples of the posteriors given by the SBI method for three sets of real data. The same priors as the ones of Figure 5 are used for the SBI method

To analyze the learning behavior of participants, we infer two parameters of our model for each participant: the learning rate η which is used to update the synaptic weights as described in (12) and indicates the participant’s learning speed, and the threshold θ, which reflects their response speed. For this estimation, we employ the Simulation-Based Inference method (Papamakarios et al. 2016) and we use data from both the learning phase and the transfer phase. This approach is tailored for models whose likelihoods are intractable but which can nonetheless be simulated. The process involves providing the model as a probabilistic program that is easy to simulate, along with a prior distribution over the parameters to be estimated. A Bayesian neural network is then trained via maximum likelihood on samples generated by the program, enabling the learning of a posterior distribution from which samples can be drawn. To simulate data for the transfer phase, we freeze the weights at their final update, $w^j_{M+1}$, as participants have already completed learning to categorize the objects. Because the lengths of participant data and simulated data did not always match, we extended the transfer phase data by appending the mean answer time of the transfer phase. This ensured that the data had the required length for applying the SBI method. We denote by $\hat{\eta }$ and $\hat{\theta }$ the median value of samples of the posterior given by the SBI method for each participant. The method is illustrated using both simulated and real data in Figures 5 and 6, respectively. On simulated data, the method accurately recovers the true parameters, although with a positive bias noticeable on the histograms of sample 2.

### Single participant analysis

**Fig. 7:**
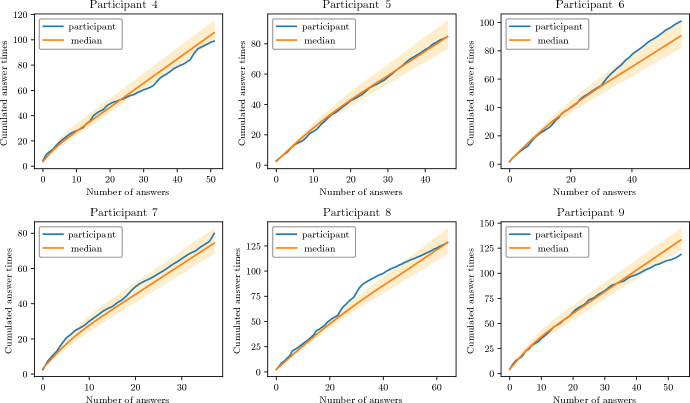
For each participant, the blue curve represents the cumulated answer times in function of the number of presented objects. The orange curves represent the median of the cumulated answer times of 100 simulations of the model with confidence interval of level 10%, with parameters $\hat{\eta }$ and $\hat{\theta }$ being the median value of samples of the posterior given by the SBI method. The same priors as the ones of Figure 5 are used for the SBI method

In Figure 7, the cumulative answer times as a function of the number of answers are compared to simulated cumulative answer times for six participants, using parameters estimated by the SBI method. Here, we used data from both the learning and transfer phase. The results indicate that the method predicts answer times accurately.

**Fig. 8:**
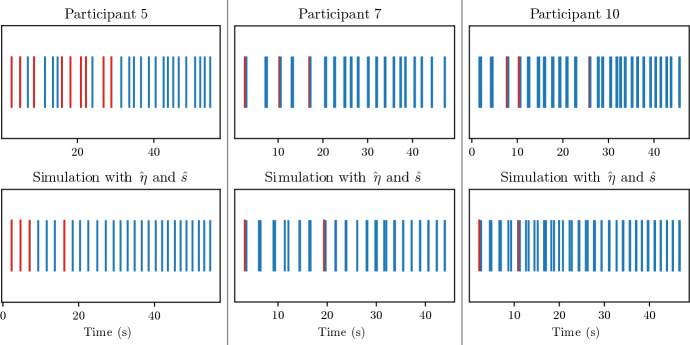
On the top, answer times of three participants during their learning phase. Blue ticks represent correct answers while red ticks represent wrong answers. On the bottom, same plots for simulated data with parameters $\hat{\eta }$ and $\hat{\theta }$ being the median value of samples of the posterior given by the SBI method for each participant. The same priors as the ones of Figure 5 are used for the SBI method

To see more precisely if one data simulation given by our network is close to real data, we represented the answer times of three participants (on top) and of three sets of simulated data (on the bottom) during the learning phase, using parameters inferred by the SBI method on Figure 8. Each blue tick represents a correct answer, and each red tick represents a wrong answer. The model again predicts answer times similar to those of the participants, albeit with greater regularity. However, it sometimes seems to make fewer errors than the participants, as in the case of participant 5. This can be attributed to the model’s reliance on synaptic weight updates: once sufficient weight has been assigned to relevant feature neurons, the probability to make a mistake diminishes significantly. In contrast, humans may continue to make errors even after grasping the underlying rule because of other cognitive phenomena such as distraction. This difference also explains why the model sometimes underestimate the total number of answers during the learning phase. Each error resets the count of consecutive correct answers to zero, requiring the participant to categorize at least 15 new objects to complete the learning phase. Consequently, each mistake significantly elongates the learning phase.

### Group analysis

**Fig. 9:**
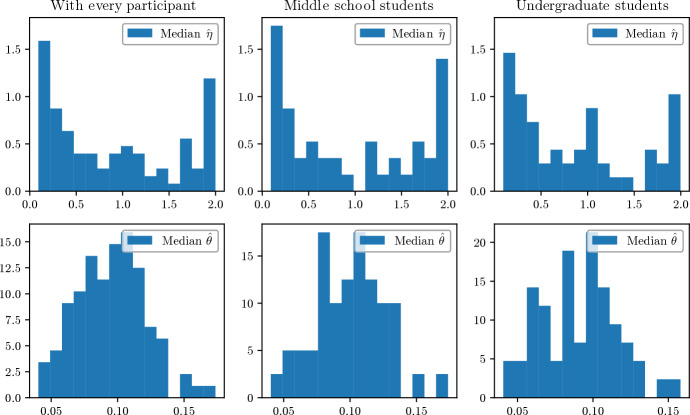
Histogram of median values of the parameters $\hat{\eta }$ and $\hat{\theta }$ given by the SBI method with on the left, every participant, on the middle, middle school students and on the right, undergraduate students. The parameters used for the SBI method are the same as in Figure 5

Figure 9 presents histograms of the parameters inferred by the SBI method, grouped by participants’ current educational level (middle school or university) and for all participants combined. The learning rate η appears to take either low or high values, with middle school students exhibiting more extreme values compared to university students. In contrast, the histograms for the parameter θ show little variation across the different groups.

**Fig. 10:**
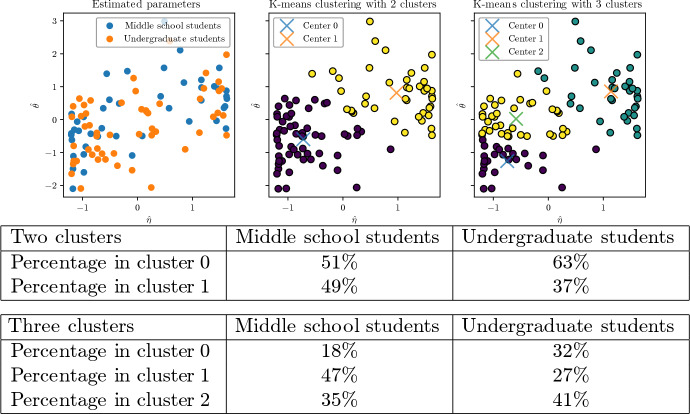
On the left, $\hat{s}$ as a function $\hat{\eta }$ for every participant, where $\hat{\theta }$ and $\hat{\eta }$ are the median values given by the SBI method. On the right, 3-means clustering of the same data. On the bottom, students distribution by cluster. The parameters used for the SBI method are the same as in Figure 5

To conduct a more detailed analysis, we applied *k*-means clustering to the two-dimensional data consisting of the parameters $\hat{\eta }$ and $\hat{\theta }$ estimated by the SBI method for each participant. The optimal number of clusters was determined using the silhouette method (details are provided in Appendix B), which suggested two reasonable options: 2 or 3 clusters. Figure 10 illustrates the results of the *k*-means algorithm applied to the estimated parameters $\hat{\eta }$ and $\hat{s}$ for both 2 and 3 clusters.

**Clustering with 2 clusters.** Cluster 0 is characterized by a low learning rate and threshold. It corresponds to participants who learn at an average or slower pace and require less evidence to make a categorization decision. This cluster includes 51% of middle school students and 63% of undergraduate students. Cluster 1, on the other hand, has a high learning rate and a slightly above-average threshold. It represents fast learners who require a higher accumulation of evidence before categorizing and comprises 49% of middle school students and 37% of undergraduate students.

A chi-square test was conducted to assess whether cluster membership is independent of the participants’ current educational level. The test yielded a p-value of 0.33, which is insufficient to reject the null hypothesis, indicating no significant evidence of dependence.

**Clustering with 3 clusters.** Clusters 0 and 1 retain approximately the same centers as in the 2-cluster analysis. Cluster 0 includes 18% of middle school students and 32% of undergraduate students, while cluster 1 comprises 47% of middle school students and 27% of undergraduate students. Cluster 2, primarily derived from points in the former cluster 0 along with a few from cluster 1, has a center characterized by a low learning rate and an average threshold. This cluster represents participants who learn at an average or slower pace and require a moderate accumulation of evidence before categorizing, encompassing 35% of middle school students and 41% of undergraduate students.

A chi-square test was conducted to evaluate whether cluster membership is independent of current educational level. The test resulted in a p-value of 0.11, which, while not statistically significant, provides some indication that cluster membership might depend on the current level of study. Specifically, slow-learning participants requiring less evidence to categorize appear more frequently among undergraduate students, while fast-learning participants requiring higher evidence accumulation tend to be more prevalent among middle school students.

**Fig. 11:**
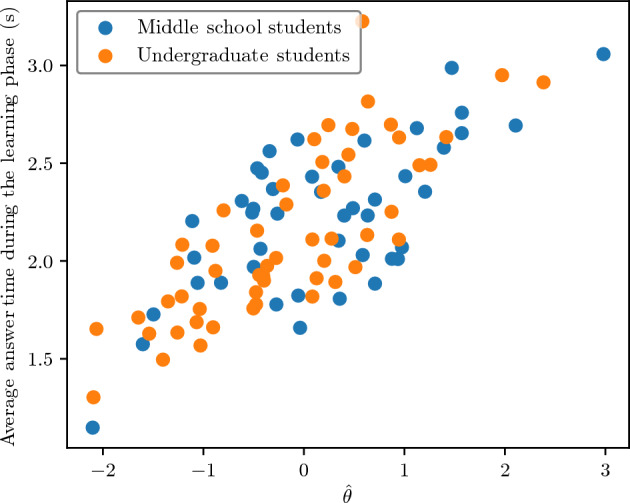
Average answer time of each participant during the learning phase (including the 15 objects to categorize perfectly) as a function of $\hat{\theta }$. The parameters used for the SBI method are the same as in Figure 5

Finally, we examined the relationship between the threshold estimated by the SBI method and each participant’s average answer time during the learning phase, as shown in Figure 11. The results indicate that the average answer time appears to increase linearly with $\hat{\theta }$, which is consistent with the model predictions.

## Conclusion

This paper outlines key elements that establish a connection between drift diffusion models (DDMs), commonly used in cognitive science to simulate decision-making tasks, and biologically plausible networks of spiking neurons. First, we conducted a mathematical analysis of both the drift diffusion model and the Poisson counter model, specifying explicit conditions under which these models yield accurate decisions. This analysis demonstrated the similarity in their behavior. Subsequently, we established a coupling result between the processes of the two models and derived a bound on their hitting times, confirming that both models produce comparable reaction times and decisions. These findings represent an initial step towards demonstrating that DDMs can be derived from spiking neuron-based models.

To go further and in order to establish that DDMs can be derived from biologically realistic networks of spiking neurons which can learn to make correct decisions, we proposed a novel spiking neural network model tailored for decision-making task which is provably close to the drift diffusion model. Unlike the drift diffusion and Poisson counter models, the model does not assume any prior knowledge of the categories; its accuracy emerges from a learning phase. Additionally, the model is more biologically relevant than the Poisson counter model, as it incorporates interacting spiking neurons that learn to represent complex concepts, rather than individual neurons directly encoding decisions. We conducted an asymptotic analysis of the network’s behavior and identified conditions under which it achieves accurate decisions. Moreover, we established a coupling result between our model and the DDM, providing strong evidence for the biological plausibility of DDMs and validating their widespread use in cognitive science. In the literature, coupling between Poisson and Brownian processes are well-known results. However, coupling between Hawkes and Brownian processes are less known. Recent results have been obtained in one dimension (Besançon et al. 2024) or for mean field limit of systems of interacting neurons (Erny et al. 2023). However, to the best of our knowledge, this is the first time such a result has been established for a network with a “perceptron”-like structure.

Additionally, we developed a cognitive experiment to evaluate our model’s predictions, enabling us to derive meaningful insights into the participant’s cognitive behavior and demonstrating the practical applicability of our model.

This study represents an important advancement in incorporating biologically relevant neural mechanisms into cognitive models, allowing us to better understand how neural dynamics lead to certain behaviors. There are several promising avenues for further improving the model. We showed that the model tends to produce more regular reaction times and make fewer errors compared to humans, highlighting the potential value of exploring ways to make it more realistic. Additionally, applying our model to more complex experiments could provide valuable insights into intricate behaviors. This seems feasible, as we showed in Jaffard et al. (2026) that introducing hidden layers enables the modeling of more complex concepts. Finally, an ambitious direction for future research would be to extend this work to other spiking neural networks, thereby demonstrating that DDMs can be approximated by alternative network types commonly used in neuroscience.

**Supplementary information**

The code and data used to produce the numerical results can be found at the following link https://github.com/SophieJaffard/MEL.

## Figures and Tables

**Fig. 12:**
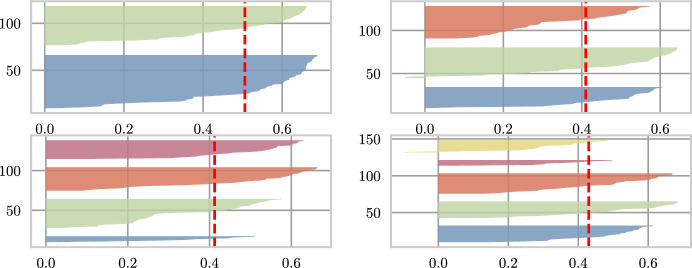
Silhouette analysis of the data with 2, 3, 4 and 5 clusters

## Appendix A Table of notations

For parameters $p_1,\cdots , p_k$, we denote by $\Box _{p_1,\cdots ,p_k}$ any positive constant depending only on parameters $p_1,\cdots , p_k$. The value of $\Box _{p_1,\cdots ,p_k}$ can change from line to line and it is not necessarily equal on both sides of an inequality.

For $a,b\in \mathbb {R}$, we denote $a\vee b :=\max (a,b)$ and $a\wedge b :=\min (a,b)$. Also when quantities have several indices, the dropping of one index corresponds to the sequence on this index. For instance, $\gamma = (\gamma ^j_o)_{j\in J, o\in \mathcal {O}}$ whereas $\gamma _o = (\gamma ^j_o)_{j\in J}$ and $\gamma ^j = (\gamma ^j_o)_{o\in \mathcal {O}}$. If two sets of indices are available, typically *I* and *J*, to avoid confusion, we use the set instead of the index. For instance, $\gamma ^I=(\gamma ^i_o)_{i\in I, o\in \mathcal {O}}$. Also $\Vert \Vert _{\infty }$ is the infinite norm.

For a set *E* and a quantity $x_e \in \mathbb {R}$ indexed by e∈E, we denote by $\langle x_e \rangle _{e\in E} :=\frac{1}{|E |}\sum _{e\in E} x_e$ its empirical mean. Table  1

**Table 1:** Table of notations

Notation	Description (page of introduction)
$\mathcal {O}$	set of object natures (page 5)
*o*	nature of an object (page 7)
*J*	set of categories and output neurons (page 14)
*j*	index of an output neuron or a category (page 14)
*T*	maximal duration of an object presentation (page 7)
*m*	index of an object (page 7)
θ	threshold (page 7)
$W_m$	|J|-dimensional drifted Brownian motion $W_m = (W^j_{m})_{j\in J}$ (page 7)
$W^j_{m,t}$	evidence accumulation in favor of category *j* at time *t* of object *m* for the DDM (page 7)
$\mu ^j_o$	drift of the process $W^j_{m}$ when the nature of object *m* is *o* (page 7)
$\tau (Z^j)$	hitting time of process $Z^j_{m}$ for $Z\in \{W,\Pi ,N\}$ (pages 7, 9 and 13)
$\hat{\jmath }$	index of the first process to reach threshold θ (pages 7, 9 and 13)
τ(Z)	reaction time of the model for $Z\in \{W,\Pi ,N\}$ (pages 7, 9 and 13)
$\Pi _m$	|J|-dimensional Poisson process $\Pi _m = (\Pi ^j_{m})_{j\in J}$ (page 9)
$\Pi ^j_{m,t}$	spike count of neuron *j* on [0, *t*] for object *m* in the Poisson counter model, *i.e., *(page 9)
$\gamma ^j_o$	intensity of output neuron *j* when the presented object has nature *o* for the Poisson counter model (page 9)
*n*	number of neurons coding for each category in the framework of the coupling results (page 10)
*M*	number of objects presented to the network during the learning phase (page 13)
*I*	set of features and input neurons (page 14)
*i*	index of an input neuron or a feature (page 14)
$\gamma ^i_o$	intensity of input neuron *i* when presented with an object having nature *o* for our model (page 14)
$I^j$	set of input which are the most sensible to category *j* (page 17)
$\widehat{\gamma }^i_o$	empirical firing rate of input neuron *i* on $[0,T_{\text {min}}]$(page 15)
$T_{\text {min}}$	minimum duration of the presentation of an object (page 13)
$N_m$	|J|-dimensional Hawkes process $N_m = (N^j_{m})_{j\in J}$ (page 13)
$N^j_{m,t}$	evidence accumulation in favor of category *j* at time *t* of object *m* *i.e., *spike count of neuron *j* until time *t* (page 14)
$\lambda ^j_{m,t}$	conditional intensity of output neuron *j* at time *t* of object *m* for our model (page 14)
$w^{i\rightarrow j}_m$	synaptic weight between input neuron *i* and output neuron *j* (page 16)
*g*	self-exciting function of the Hawkes process (page 15)
$h_t(u)$	$h_t(u) = \int _u^t g(s-u) ds$ (page 57)
$\mathbb {G}(t)$	$\mathbb {G}(t) = \int _0^t \int _0^s g(s-u)du ds$ (page 19)
$\Lambda ^j_{m,t}$	compensator of the process $(N^j_{m,s})_{s\ge 0}$ at time *t* (page 57)
$g^{i\rightarrow j}_m$	gain of input neuron *i* w.r.t. output neuron *j* after the presentation of the $m^{th}$ object (page 15)
$G^{i\rightarrow j}_m$	cumulated gain of input neuron *i* w.r.t. output neuron *j* after the presentation of the $m^{th}$ object $\sum _{m'=1}^m g^{i\rightarrow j}_{m'}$ (page 16)
$\eta =\eta _0/\sqrt{M}$	learning rate of the learning rule given by EWA ((13) page 16)
$\mathcal {F}_m$	σ-algebra generated by every event which happened until the end of the presentation of the $m^{th}$ object (page 16)
$d^{i\rightarrow j}$	feature discrepancy of input neuron *i* w.r.t. output neuron *j* (page 17)
$I^j$	$I^j :=\arg \max _{i\in I} d^{i\rightarrow j}$ (page 17)
$\delta ^j$	$\max _{i\in I} d^{i\rightarrow j} - \max _{i\in I\setminus I^j} d^{i\rightarrow j}$(page 17)
$w^{i\rightarrow j}_\infty$	limit synaptic weight between input neuron *i* and output neuron *j* (page 17)
${\boldsymbol{\Delta }}_{\mu }$	discrepancy between the drift of the true category process and the other processes for the DDM (page 8)
${{\boldsymbol{\Delta }}_\gamma }$	discrepancy between the firing rate of the true category process and the other processes for the Poisson counter model (page 10)
${{\boldsymbol{\Delta }}_\lambda }$	discrepancy between the firing rate of the true category process and the other processes for our model (page 19)

## Appendix B details on the clustering analysis

The number of clusters used to perform the k-means algorithm in Section 4.7 was determined using the silhouette method implemented in scikit-learn. The silhouette plot, available in Figure 12, illustrates how well each point aligns with its assigned cluster compared to neighboring clusters. Silhouette coefficients close to 1 indicate that a sample is well-separated from neighboring clusters, while a coefficient of 0 suggests that the sample lies near the boundary between two clusters. The dotted red line represents the average silhouette score across all samples for a given number of clusters. In order to choose a meaningful number of clusters, two criteria should be respected: each clusters should have points above the average silhouette score, and the several clusters should have comparable sizes. Therefore, k=2 and k=3 are sensible choices.

## Appendix C proofs

### C.1 Preliminary lemmas

#### Lemma C.1

Let $(W_t)_{t\ge 0}$ be a Brownian motion with drift μ and scaling σ. Let T>0,β>0. Then

$$\begin{aligned} \mathbb {P}\left( \sup _{0\le t \le T} |W_t - \mu t | < \sqrt{2T\sigma ^2\log \left( \frac{2}{\beta }\right) } \right) \ge 1 - \beta . \end{aligned}$$

#### Proof

The process $\frac{W_t - \mu t}{\sigma }$ is a standard Brownian motion. Let z>0. According to Doob’s inequality (see for instance Proposition 1.5 p.53 of Revuz and Yor (1999)), we have with probability more than $1-2e^{-\frac{z^2}{2T}}$ that

$$\begin{aligned} \sup _{0\le t \le T} \frac{|W_t - \mu t |}{\sigma } < z. \end{aligned}$$

Let us choose *z* such that $2e^{-\frac{z^2}{2T}} = \beta$, *i.e.,* $z = \sqrt{2T \log \left( \frac{2}{\beta } \right) }$. Then with probability more than 1-β we have

$$\begin{aligned} \sup _{0\le t \le T} |W_t - \mu t | < \sqrt{2T\sigma ^2\log \left( \frac{2}{\beta }\right) }. \end{aligned}$$

□

The following Lemma is an adaptation of Corollary 5.2 p. 357 of Ethier and Kurtz (1986).

#### Lemma C.2

There exists absolute constants a,b,d>0 and a measurable function $G: S(\mathbb {R}_+)\times [0,1]\rightarrow S(\mathbb {R}_+)$, with $S(\mathbb {R}_+)$ the Skorohod space such that if $(\Pi _t)_{t\ge 0}$ is an homogeneous counting Poisson process of rate 1 and *V* is an independent uniform random variable on [0, 1], then

- $(W_t)_{t\ge 0}= G((\Pi _t)_{t\ge 0},V)$ is a Brownian motion starting at 0, with drift 1 and variance 1 (i.e. $(B_t)_{t\ge 0}$ such that $B_t=W_t-t$ is a standard centered Brownian motion), and
- for all x>0
  $$\begin{aligned} \mathbb {P}\left( \sup _{t\ge 0} \frac{|\Pi _t-W_t|}{\log (t \vee 1+1)} \ge a+x\right) \le b e^{-d x}. \end{aligned}$$

#### Proof

We start with the Komlós-Major-Tusnády inequality (Komlós et al. 1975, 1976) (see Theorem 5.1 p.356 of Ethier and Kurtz (1986) for the same formulation as here) applied to the i.i.d. sequence $(\xi _k)_{k\in \mathbb {N}}$ of Poisson r.v. with parameter 1. KMT says that there exists such a sequence as well as a Brownian motion $W_t$ such that $\mathbb {E}(W_1)=1$ and $\mathop {\text {Var}}(W_1)=1$ and positive constants a, b and d such that for all x>0 and all $n \in \mathbb {N}, n\ge 1$,

$$\begin{aligned} \mathbb {P}(\max _{1\le k \le n} |S_k-W_k| > a \log (n) +x) \le b e^{-dx}, \end{aligned}$$

with $$S_k=\sum _{i=1}^k \xi _i$$.

Remark that we can also write for all t≥0, $W_t=t+B_t$ where $(B_t)_{t\ge 0}$ is a standard Brownian (meaning a centered Brownian such that $\mathop {\text {Cov}}(B_s,B_t)=s\wedge t$).

Conditionally to all the $\xi _k$’s, one can construct a Poisson process $(\Pi _t)_{t\ge 0}$ such that for any *k*, $\Pi _k=S_k$. Indeed, for each *k*, conditionally to each $\xi _k$, and independently of anything else, we can pick $\xi _k$ i.i.d. uniform variables on [0, 1] and order them. They constitute the times of the jumps of a Poisson process $(\tilde{\Pi }^k_t)_{t\in [0,1]}$, such that $\tilde{\Pi }^k_1=\xi _k$.

Then, we define $\Pi _t=S_{\lfloor t\rfloor }+\tilde{\Pi }^{\lfloor t\rfloor }_{t-\lfloor t\rfloor }$ for any t≥0. The process $(\Pi _t)_{t\ge 0}$ is therefore a Poisson process of intensity 1 such that for all $k\in \mathbb {N}$, $S_k=\Pi _k$.

Let us fix T≥0 and $n=\lfloor T\rfloor$. Then for all $0\le t\le T$

$$\begin{aligned} |\Pi _t-W_t|&\le |\Pi _{\lfloor t \rfloor }-W_{\lfloor t \rfloor }|+ |\Pi _t-\Pi _{\lfloor t \rfloor }| + |W_t-W_{\lfloor t \rfloor }|\\&\le |\Pi _{\lfloor t \rfloor }-W_{\lfloor t \rfloor }| + \xi _{\lfloor t \rfloor +1}+ \zeta _{\lfloor t \rfloor }, \end{aligned}$$

with $$\zeta _k= \sup _{s\in [0,1)}|W_{k+s}-W_k|.$$

So

$$\begin{aligned} \sup _{t\in [0,T)} |\Pi _t-W_t| \le \max _{k=1,\cdots ,n} |S_k-W_k| + \max _{k=1,\cdots ,n+1} \xi _k +\max _{k=0,\cdots ,n} \zeta _k. \end{aligned}$$

But the $\xi _k$’s are iid variables with finite exponential moment of every order. So by union bound and Markov inequality, we have for every y≥0

$$\begin{aligned} \mathbb {P}(\max _{k=1,\cdots ,n+1} \xi _k \ge y ) = \mathbb {P}(\max _{k=1,\cdots ,n+1} e^{d\xi _k} \ge e^{dy}) \le (n+1) \mathbb {E}(e^{d\xi _1}) e^{-dy}. \end{aligned}$$

The same holds for the $\zeta _k$’s since they also have exponential moment of every order.

Therefore taking y=log(n+1)/d+x/3, and using KMT with *x*/3, we obtain that with probability larger than $1- (b+\mathbb {E}(e^{d\xi _1})+\mathbb {E}(e^{d\zeta _1})) e^{-dx/3}$,

$$\begin{aligned} \sup _{t\in [0,T)} |\Pi _t-W_t| \le (a+2/d) \log (n+1) + x. \end{aligned}$$

In other words, there exist constants $a^{\prime },b^{\prime },d^{\prime }$ such that for all T>0

$$\begin{aligned} \mathbb {P}( \sup _{t\in [0,T)} |\Pi _t-W_t| \ge a' \log (T+1) +x) \le b' e^{-d'x}. \end{aligned}$$

Now we want to get rid of *T*. We can decompose the event as follows

$$\begin{aligned}&\left\{ \sup _{t\ge 0} \frac{|\Pi _t-W_t|}{\log (t \vee 1+1)} \ge y\right\} \\&= \left\{ \sup _{t\in [0,2)} \frac{|\Pi _t-W_t|}{\log (t \vee 1+1)} \ge y\right\} \cup \bigcup _{n\ge 1} \left\{ \sup _{t\in [2^n, 2^{n+1})} \frac{|\Pi _t-W_t|}{\log (t \vee 1+1)} \ge y\right\} . \end{aligned}$$

But the probability of the first event is bounded, thanks to the previous computations, by

$$\begin{aligned} \mathbb {P}(\sup _{t\in [0,2)} |\Pi _t-W_t| \ge y \log (2)) \le b' e^{-d'(y \log (2)- a'\log (3))}, \end{aligned}$$

For the other events

$$\begin{aligned}&\mathbb {P}(\sup _{t\in [2^n,2^{n+1})} |\Pi _t-W_t| \ge y \log (2^n)) \\&\le \mathbb {P}(\sup _{t\in [0,2^{n+1})} |\Pi _t-W_t| \ge y \log (2^n)) \le b' e^{-d'(y\log (2^n)-a'\log (2^{n+1}+1))}. \end{aligned}$$

But $\log (2^{n+1}+1)=\log (2^n)+\log (2+2^{-n})\le \log (2^n)+\log (3)$ for all n≥0, so

$$\begin{aligned} \mathbb {P}\left( \sup _{t\ge 0} \frac{|\Pi _t-W_t|}{\log (t \vee 1+1)} \ge y\right) \le b'&e^{-d'(y-a') \log (2) + d'a' \log (3)} \\&+ \sum _{n\ge 1} b' e^{-d'(y-a') \log (2^n)+ d'a'\log (3)} \end{aligned}$$

so the upper bounds reads

$$\begin{aligned} b' e^{d'a'\log (3)} \left( e^{-d'(y-a') \log (2)} + \sum _{n\ge 1} 2^{-n d'(y-a')}\right) . \end{aligned}$$

If $y=a^{\prime }+u$, with u>0, we get

$$\begin{aligned} b' e^{d'a'\log (3)} \left( 2^{-d'u} + 2^{-d'u}(1-2^{-d'u})^{-1}\right) . \end{aligned}$$

By taking $u=1/d^{\prime }+x$ with x>0, we get that $$(1-2^{-d'u})^{-1}\le 1/2$$, so that there exists a",b",d">0 such that

C1 $$\begin{aligned} \mathbb {P}\left( \sup _{t\ge 0} \frac{|\Pi _t-W_t|}{\log (t \vee 1+1)} \ge a"+x\right) \le b" e^{-d"x}. \end{aligned}$$

Now to go further, let us quote Lemma 3.12 of the Arxiv version Prodhomme (2020) of Prodhomme (2023).

#### Lemma C.3

Let $E_1$ and $E_2$ be two complete separable metric spaces and let μ be a probability on $(E_1\times E_2, \mathcal {B}(E_1)\otimes \mathcal {B}(E_2))$. Let $\mu _1$ be the first marginal of μ. Then there exists a measurable function $G: E_1 \times [0,1]\rightarrow E_2$ such that if $(X_1,V)\sim \mu _1\otimes \mathcal {U}([0,1])$, then $(X_1,G(X_1,V))\sim \mu .$

Both $(\Pi _t)_{t\ge 0}$ and $(W_t)_{t\ge 0}$ live in the Skorohod space $\mathcal {D}_{\mathbb {R}}([0,+\infty ))$ which is separable and complete (polish). So, one can apply the lemma of Prodhomme: for any Poisson process $(\Pi _t)_{t\ge 0}$ with rate 1, by adding an extra uniform variable *V*, independent of anything else, one construct a drifted Brownian $(W_t)_{t\ge 0}=G((\Pi _t)_{t\ge 0},V)$, such that $(\Pi _t)_{t\ge 0}$ and $(W_t)_{t\ge 0}$ satisfy (C1). □

#### Corollary C.4

Let α>0 and $(\Pi _t)_{t\ge 0}$ a homogeneous Poisson process with intensity μ>0. Then there exists a Brownian motion $(W_t)_{t\ge 0}$ with drift μ and scaling $\sqrt{\mu }$ and absolute positive constants C,ζ,K such that

$$\begin{aligned} \mathbb {P}\left( \sup _{t\ge 0} \frac{|\Pi _t-W_t|}{\log (\mu t \vee 1+1)} \ge C +\zeta \log (K\alpha ^{-1})\right) \le \alpha . \end{aligned}$$

#### Proof

Let α>0. Let $\Pi _t$ a homogeneous Poisson process with intensity μ>0. Then $\tilde{\Pi }_t :=\Pi _{\mu ^{-1} t}$ is a standard Poisson process so acccording to Lemma C.2, there exists a standard Brownian motion $B_t$ and absolute constants a,b,d>0 such that for all x>0

$$\begin{aligned} \mathbb {P}\left( \sup _{t\ge 0} \frac{|\tilde{\Pi }_t-t - B_t|}{\log (t \vee 1+1)} \ge a+x\right) \le b e^{-d x}. \end{aligned}$$

Let $$W_t :=B_{\mu t} + \mu t$$. Then

$$\begin{aligned} \mathbb {P}\left( \sup _{t\ge 0} \frac{|\Pi _t-W_t|}{\log (\mu t \vee 1+1)} \ge a+x\right) \le b e^{-d x}. \end{aligned}$$

By choosing *x* such that $b e^{-d x} = \alpha$, *i.e.,* $x=\frac{1}{d}\log (b/\alpha )$, C=a, K=b and $\zeta = d^{-1}$ we have the result. □

The following Lemma and its proof can be found in Ost and Reynaud-Bouret (2023) (Lemma 7.1).

#### Lemma C.5

Let *X* be a Poisson random variable with parameter γ>0. For any x≥0,

C2 $$\begin{aligned} \mathbb {P}(X \ge \gamma (1+x)) \le \exp \left( -\frac{\gamma x^2}{2(1+\frac{x}{3})}\right) \end{aligned}$$

and for any $0\le x \le \gamma$,

C3 $$\begin{aligned} \mathbb {P}(X \le \gamma - x) \le \exp \left( -\frac{x^2}{2\gamma }\right) . \end{aligned}$$

#### Corollary C.6

Let α>0. Let *X* be a Poisson random variable with parameter γ>0. Then

1. If $\gamma \ge 2\log (\alpha ^{-1})$, we have
  C4 $$\begin{aligned} \mathbb {P}\left( X \ge \gamma - \sqrt{2\gamma \log (\alpha ^{-1})}\right) \ge 1-\alpha . \end{aligned}$$
2. If $\gamma \ge \dfrac{8}{3}\log (\alpha ^{-1})$, we have
  C5 $$\begin{aligned} \mathbb {P}\left( X \le \gamma + \sqrt{\frac{8}{3}\gamma \log (\alpha ^{-1})}\right) \ge 1- \alpha . \end{aligned}$$
3. If $\gamma \ge \dfrac{8}{3}\log (2\alpha ^{-1})$, we have
  C6 $$\begin{aligned} \mathbb {P}\left( |X-\gamma | \le \sqrt{\frac{8}{3}\gamma \log (2\alpha ^{-1})}\right) \ge 1 - \alpha . \end{aligned}$$

#### Proof

1 Assume $\gamma \ge 2\log (\alpha ^{-1})$, then $x:=\sqrt{2\gamma \log (\alpha ^{-1})}$ satisfies $0\le x \le \gamma$. We apply (C3) and deduce

$$\begin{aligned} \mathbb {P}\left( X \le \gamma - \sqrt{2\gamma \log (\alpha ^{-1})}\right) \le \alpha , \end{aligned}$$

and deduce (C4).

Proof of 2: Thanks to the assumption, $u:=\sqrt{\dfrac{8}{3}\gamma \log (\alpha ^{-1})} \le \gamma$. The inequality (C2) can be rewritten as

$$\begin{aligned} \mathbb {P}(X \ge \gamma + u) \le \exp \left( -\frac{u^2}{2\gamma +\frac{2u}{3}}\right) \le \exp \left( -\frac{3u^2}{8\gamma }\right) . \end{aligned}$$

Proof of 3: We apply (C4) and (C5) with α/2 and conclude the proof. □

#### Lemma C.7

Let $\Pi _t$ be a homogeneous Poisson process with parameter μ>0. Then for every x≥0 we have

$$\begin{aligned} \mathbb {P}\left( \sup _{0\le t \le T}( \Pi _t - \mu t) ) \ge \mu T x\right) \le \exp \left( - \frac{\mu T x^2}{2(1 + x /3)}. \right) \end{aligned}$$

and for every $x\in [0, \mu T]$ we have

$$\begin{aligned} \mathbb {P}\left( \sup _{0\le t \le T}( \mu t - \Pi _t ) \ge x\right) \le \exp \left( - \frac{x^2}{2\mu T}. \right) \end{aligned}$$

#### Proof

The process $(\Pi _t - \mu t)_{t\ge 0}$ is a right-continuous martingale. Let C>0,ξ>0. Then $(\exp (\xi (\Pi _t - \mu t))_{t\ge 0}$ is a right-continuous submartingale. Therefore, according to the first submartingale inequality (Theorem 3.8 page 13 of Karatzas and Shreve (1998)), we have

$$\begin{aligned} \mathbb {P}\left( \sup _{0\le t \le T} \exp (\xi (\Pi _t - \mu t) ) \ge C\right) \le \frac{\mathbb {E}[e^{\xi (\Pi _T - \mu T) }]}{C}. \end{aligned}$$

We choose $C = \exp (\xi \mu T x)$ and mimic the proof of Lemma C.5 to obtain:

$$\begin{aligned} \mathbb {P}\left( \sup _{0\le t \le T}( \Pi _t - \mu t) ) \ge \mu T x\right) \le \exp \left( - \frac{\mu T x^2}{2(1 + x /3)} \right) . \end{aligned}$$

Similarly, the process $(\mu t - \Pi _t)_{t\ge 0}$ is a right-continuous martingale so for ξ>0, the process $(\exp (\xi (\mu t - \Pi _t))_{t\ge 0}$ is a right-continuous submartingale. Therefore

$$\begin{aligned} \mathbb {P}\left( \sup _{0\le t \le T} \exp (\xi (\mu t - \Pi _t) ) \ge C\right) \le \frac{\mathbb {E}[e^{\xi (\mu T - \Pi _T) }]}{C}. \end{aligned}$$

Again, we can mimic the proof of Lemma C.5 and we get the same bound for $x\in [0, \mu T]$:

$$\begin{aligned} \mathbb {P}\left( \sup _{0\le t \le T}( \mu t - \Pi _t ) \ge x\right) \le \exp \left( - \frac{x^2}{2\mu T} \right) . \end{aligned}$$

□

#### Corollary C.8

Let β>0 and $T\ge \frac{8}{3\mu }\log (2/\beta )$. Let $\Pi _t$ be a homogeneous Poisson process with parameter μ>0. Then

$$\begin{aligned} \mathbb {P}\left( \sup _{0\le t \le T}|\Pi _t - \mu t | ) \ge \sqrt{\frac{8}{3}\mu T \log \Big (\frac{2}{\beta }\Big )}\right) \le \beta . \end{aligned}$$

#### Proof

Similarly as in the proof of (C6), by combining the two inequalities given by Lemma C.7, we have that for every $0\le x \le \mu T$

$$\begin{aligned} \mathbb {P}\left( \sup _{0\le t \le T}|\Pi _t - \mu t | ) \ge x\right) \le 2\exp \left( - \frac{3 x^2}{8\mu T} \right) . \end{aligned}$$

Let us choose *x* such that $2\exp \left( - \frac{3 x^2}{8\mu T}\right) = \beta$, *i.e.,* $x = \sqrt{\frac{8}{3}\mu T \log \Big (\frac{2}{\beta }\Big )}$. The condition $x\le \mu T$ becomes $T\ge \frac{8}{3\mu }\log (2/\beta )$. □

#### Lemma C.9

There exist constants $c,c^{\prime },c">0$ such that if $$(B_t)_{t\ge 0}$$ a standard centered Brownian motion then for all x>0

$$\begin{aligned} \mathbb {P}\left( \sup _{t, u \ge 0} \frac{|B_t-B_u|}{\sqrt{(|t-u|+2)[\log (t+1)+\log (u+1)+2x]}} \ge c\right) \le c' e^{-c'' x}. \end{aligned}$$

This lemma resembles Lemma 6 of Chevallier et al. (2021), which is probably tighter, but is valid only for t,u≤T whereas our version is on all $\mathbb {R}_+$.

#### Proof

Remark that

$$\begin{aligned}&\sup _{t,u \ge 0} \frac{|B_t-B_u|}{\sqrt{(|t-u|+2)[\log (t+1)+\log (u+1)+2x]}} \\&=\sup _{t\ge u \ge 0} \frac{|B_t-B_u|}{\sqrt{(|t-u|+2)[\log (t+1)+\log (u+1)+2x]}}, \end{aligned}$$

because the quantity on which we are taking the supremum is invariant by exchange of *t* and *u*.

We start from the article Robbins and Siegmund (1970), where Example 3 page 88 says that for all y>0,

C7 $$\begin{aligned} \mathbb {P}\left( \sup _{t\ge 0} \frac{|B_t|}{\sqrt{(t+1)[\log (t+1)+2y]}}\ge 1\right) \le e^{-y}. \end{aligned}$$

Let us now look at the event

$$\begin{aligned}&\left\{ \sup _{t \ge u \ge 0} \frac{|B_t-B_u|}{\sqrt{(|t-u|+2)[\log (t+1)+\log (u+1)+2x]}} \ge a \right\} = \\&\quad \bigcup _{n\in \mathbb {N}} \left\{ \sup _{t \ge u \ge 0, u\in [n,n+1)} \frac{|B_t-B_u|}{\sqrt{(|t-u|+2)[\log (t+1)+\log (u+1)+2x]}} \ge a \right\} . \end{aligned}$$

But for fixed $n\in \mathbb {N}$, for every $t\ge u, u\in [n,n+1)$

$$\begin{aligned}&\frac{|B_t-B_u|}{\sqrt{(|t-u|+2)[\log (t+1)+\log (u+1)+2x]}} \\ &\quad \le \frac{|B_t-B_n|}{\sqrt{(|t-u|+2)[\log (t+1)+\log (u+1)+2x]}}\\&\quad +\frac{|B_u-B_n|}{\sqrt{(|t-u|+2)[\log (t+1)+\log (u+1)+2x]})} \\&\quad \le \frac{|B_t-B_n|}{\sqrt{(t-n+1)[\log (t+1)+\log (n+1)+2x]}}\\&\quad +\frac{|B_u-B_n|}{\sqrt{(|u-n|+1)[\log (n+1)+\log (u+1)+2x]})}. \end{aligned}$$

This leads to

$$\begin{aligned}&\left\{ \sup _{t \ge u \ge 0, u\in [n,n+1)} \frac{|B_t-B_u|}{\sqrt{(|t-u|+2)[\log (t+1)+\log (u+1)+2x]}} \ge a \right\} \subset \\&\quad \left\{ \sup _{t \ge n} \frac{|B_t-B_n|}{\sqrt{(t-n+1)[\log (t+1)+\log (n+1)+2x]}} \ge a/2 \right\} \\&\quad \bigcup \left\{ \sup _{u \ge n} \frac{|B_u-B_n|}{\sqrt{(|u-n|+1)[\log (n+1)+\log (u+1)+2x]})} \ge a/2 \right\} . \end{aligned}$$

But in each case, with a≥2 we can apply (C7) to respectively the Brownian $(B_t-B_n)_{t\ge n}$ and the Brownian $(B_u-B_n)_{t\ge n}$ with $y=\frac{a}{2}(\log (n+1)/2+x)$. Hence by union bound, we get that

$$\begin{aligned} \mathbb {P}\left( \sup _{t \ge u \ge 0} \frac{|B_t-B_u|}{\sqrt{(|t-u|+2)[\log (t+1)+\log (u+1)+2x]}} \ge a \right)&\le \sum _{n=0}^{\infty } 2 e^{- \frac{a}{2}(\log (n+2)/2+x)} \\&\hspace{-1cm}\le 2 \sum _{n=0}^{\infty } (n+1)^{-a/4} e^{- ax/2} \end{aligned}$$

By taking a=8 for instance, the previous sum is finite and we get exactly the result. □

### C.2 Proof of Theorem 2.3

Let us prove this more precise version of Theorem 2.3.

#### Theorem C.10

Let $\alpha \in (0,1)$ and let *m* be a fixed positive integer. Let ${\boldsymbol{\Delta }}_{\mu }>0$ and suppose Assumption 2.2 (Margin(${\boldsymbol{\Delta }}_{\mu }$)) holds. Then if $T \ge \Box \max \left( \frac{\Vert \mu \Vert _{\infty }}{{\boldsymbol{{\Delta }}}^2_\mu } \log \left( \frac{\Vert \mu \Vert _{\infty }^2}{{\boldsymbol{{\Delta }}}^2_\mu }\right) ,\right. \left. \Vert \mu \Vert _\infty ^{-1} \log \left( \frac{|J |}{\alpha }\right) \right)$ and if the threshold θ satisfies

$$\begin{aligned} \theta > \square \frac{\Vert \mu \Vert _\infty }{{\boldsymbol{{\Delta }}}^2_\mu }\max \left( \log \left( \frac{4\Vert \mu \Vert _{\infty }^2}{{\boldsymbol{{\Delta }}}^2_\mu }\right) , \log (|J|\alpha ^{-1})\right) \end{aligned}$$

and

$$\begin{aligned} \theta < {\boldsymbol{\Delta }}_{\mu }T - \sqrt{(\Vert \mu \Vert _{\infty } T+1)(\log (\Vert \mu \Vert _{\infty }T+1)+2\log (|J |\alpha ^{-1}))} \end{aligned}$$

then with probability larger than 1-α we have that for all nature $o\in \mathcal {O}$, if we denote by $j^*\in J$ its category (i.e. $o\in j^*$), the choice $\hat{\jmath }$ (3) and the reaction time $\tau (W^{\hat{\jmath }})$ (2) that take place at the presentation of the $m^{th}$ object of nature *o*, satisfy

C8 $$\begin{aligned} \hat{\jmath }= j^* \quad \text{ and }\quad \tau (W^{\hat{\jmath }}) < T. \end{aligned}$$

We start from inequality (C7) which says that for all y>0, if $(B_t)_{t\ge 0}$ is a standard Brownian motion then

$$\begin{aligned} \mathbb {P}\left( \sup _{t\ge 0} \frac{|B_t|}{\sqrt{(t+1)[\log (t+1)+2y]}}\ge 1\right) \le e^{-y}. \end{aligned}$$

Let α>0, $m\in \mathbb {N}^*$, $o\in \mathcal {O}$ the nature of object *m* and $j^*$ its category. Let $\tilde{J} :=\{j\in J \text { such that } \mu ^j_o >0\}$. Indeed, if $\mu _o^j=0$, then for all *t* we have $W^j_{m,t} = 0$ and the process $W^j_m$ will never reach θ. According to Assumption 2.2, we have necessarily $j^*\in \tilde{J}$. Up to consider $\tilde{J}$ instead of J, let us assume $J = \tilde{J}$.

For every j∈J, the process $W^j_{m,t}$ can be written as (1). By choosing $y = \log (|J |\alpha ^{-1})$, according to (C7), by union bound, we have with probability more than 1-α that for every j∈J,

C9 $$\begin{aligned} \sup _{t\ge 0} \frac{|B^j_t|}{\sqrt{(t+1)[\log (t+1)+2y]}}\le 1. \end{aligned}$$

In particular, for every t≥0 we have

$$\begin{aligned} W^{j^*}_{m,t}&\ge \mu ^{j^*}_{o} t - \sqrt{(\mu ^{j^*}_{o}t+1)(\log (t+1)+2y)} {=}{:}I(\mu ^{j^*}_{o},t) \end{aligned}$$

and for $j\ne j^*$, we get

C10 $$\begin{aligned} W^{j}_{m,t} \le \mu ^j_{o} t + \sqrt{(\mu ^j_{o} t+1)(\log (\mu ^j_{o} t+1)+2y)} {=}{:}S(\mu ^j_{o},t). \end{aligned}$$

A sufficient condition for $W^{j^*}_m$ to reach θ before limit duration *T* and to be the first to do so is the existence of $t_0\in [0,T]$ such that $I(\mu ^{j^*}_{o},t_0) = \theta$ and for every $j\ne j^*$, $S(\mu ^{j}_{o},t_0) < I(\mu ^{j^*}_{o},t_0)$. It would mean that at time $t_0$, $W^{j^*}_m$ has already reached θ and none of the other processes have done so.

Let $t_0\ge 0$. The condition $S(\mu ^{j}_{o},t_0) < I(\mu ^{j^*}_{o},t_0)$ is equivalent to

C11 $$\begin{aligned}&(\mu ^{j^*}_{o} - \mu ^j_{o})t_0 > \sqrt{(\mu ^j_{o} t_0+1)(\log (\mu ^j_{o} t_0+1)+2y)} \nonumber \\&\quad + \sqrt{(\mu ^{j^*}_{o}t_0+1)(\log (\mu ^{j^*}_{o}t_0+1)+2y)} \end{aligned}$$

which is implied by

C12 $$\begin{aligned} {\boldsymbol{\Delta }}_{\mu }t_0 > 2 \sqrt{(\Vert \mu \Vert _{\infty } t_0+1)(\log (\Vert \mu \Vert _{\infty } t_0+1)+2y)}. \end{aligned}$$

which is equivalent to

C13 $$\begin{aligned} {\boldsymbol{{\Delta }}}^2_\mu t_0^2 > 4 (\Vert \mu \Vert _{\infty } t_0+1)(\log (\Vert \mu \Vert _{\infty } t_0+1)+2y). \end{aligned}$$

Suppose $t_0>\frac{1}{\Vert \mu \Vert _{\infty }}$. Then a sufficient condition is

C14 $$\begin{aligned} {\boldsymbol{{\Delta }}}^2_\mu t_0^2 > 4 (2\Vert \mu \Vert _{\infty } t_0)(\log (2\Vert \mu \Vert _{\infty } t_0)+2y). \end{aligned}$$

Let us denote $v:=2\Vert \mu \Vert _{\infty } t_0$. Then (C14) can be rewritten as

C15 $$\begin{aligned} \frac{{\boldsymbol{{\Delta }}}^2_\mu }{4\Vert \mu \Vert _{\infty }^2} v > 4 \log (v) + 8 y. \end{aligned}$$

Let $x :=\frac{{\boldsymbol{{\Delta }}}^2_\mu }{4\Vert \mu \Vert _{\infty }^2} v$. Then a sufficient condition is

C16 $$\begin{aligned} x&\ge 4 \log (x) + 4 \log \left( \frac{4\Vert \mu \Vert _{\infty }^2}{{\boldsymbol{{\Delta }}}^2_\mu } \right) + 8 y. \end{aligned}$$

We can find a constant $x_0$, independent of everything, such that for every $x\ge x_0$, $\frac{x}{2}\ge 4 \log (x)$. Then if $x>x_0$ and $x/2> 4 \log \left( \frac{4\Vert \mu \Vert _{\infty }^2}{{\boldsymbol{{\Delta }}}^2_\mu } \right) + 8 y$, then (C16) holds.

To summarize, since $y=\log (|J|\alpha ^{-1})$, if

C17 $$\begin{aligned} t_0\ge \square \frac{1}{{\boldsymbol{{\Delta }}}^2_\mu }\max ( \log (\frac{4\Vert \mu \Vert _{\infty }^2}{{\boldsymbol{{\Delta }}}^2_\mu }), \log (|J|\alpha ^{-1})){=}{:}t_-, \end{aligned}$$

we have that $I(\mu ^{j^*}_{o},t_0)> S(\mu ^j_{o},t_0)$ for all $j\ne j^*$.

By the assumptions of the theorem, we have that

C18 $$\begin{aligned} \theta \ge \Vert \mu \Vert _\infty t_- \ge I(\mu ^{j^*}_{o},t_-)\end{aligned}$$

and

C19 $$\begin{aligned} \theta \le {\boldsymbol{\Delta }}_{\mu }T - \sqrt{(\Vert \mu \Vert _\infty T +1)(\log (\Vert \mu \Vert _\infty T +1)+2\log (|J|\alpha ^{-1}))} \le I(\mu ^{j^*}_{o},T). \end{aligned}$$

So we have that $I(\mu ^{j^*}_{o},t_-)\le I(\mu ^{j^*}_{o},T)$ and that $\theta \in [I(\mu ^{j^*}_{o},t_-),I(\mu ^{j^*}_{o},T)]$. By continuity of $I(\mu ^{j^*}_{o},.)$, there exists $t_0$ in $[t_-,T]$ such that $I(\mu ^{j^*}_{o},t_0)=\theta$ and since $t_0\ge t_-$, we also have that $S(\mu ^j_{o},t_0)<I(\mu ^{j^*}_{o},t_0)$ for all $j\ne j^*$, which concludes the proof.

### C.3 Proof of Theorem 2.5

Let us prove the following result from which Theorem 2.5 derives.

#### Theorem C.11

Let $\alpha \in (0,1)$ and let *m* be a fixed positive integer. Let ${{\boldsymbol{\Delta }}_\gamma }>0$ and suppose Assumption 2.4 (Margin(${{\boldsymbol{\Delta }}_\gamma }$)) holds. Then if $T\ge \frac{8}{3}\log \left( \frac{2|J |}{\alpha }\right) \max \left( \gamma _{\text {min}}^{-1}, 4\Vert \gamma \Vert _\infty {{\boldsymbol{\Delta }}_\gamma }^{-2}\right)$ and if the threshold θ satisfies

C20 $$\begin{aligned} \frac{8}{3}\log \left( \frac{2|J |}{\alpha }\right) \max \left( \frac{\Vert \gamma \Vert _{\infty }}{\gamma _{\text {min}}}, 4\left( \frac{\Vert \gamma \Vert _{\infty }}{{{\boldsymbol{\Delta }}_\gamma }}\right) ^2\right) \le \theta \le {{\boldsymbol{\Delta }}_\gamma }T - \sqrt{\frac{8}{3} \Vert \gamma \Vert _{\infty } T \log \left( \frac{2|J |}{\alpha }\right) } \end{aligned}$$

where $\gamma _{\text {min}} :=\min _{o\in \mathcal {O},j\in J}\{\gamma ^i_o; \gamma ^j_o >0\}$, then with probability larger than 1-α we have that for all nature $o\in \mathcal {O}$, if we denote by $j^*\in J$ its category (i.e. $o\in j^*$), the choice $\hat{\jmath }$ (6) and the reaction time $\tau (\Pi ^{\hat{\jmath }})$ (5) that take place at the presentation of the $m^{th}$ object of nature *o*, satisfy

C21 $$\begin{aligned} \hat{\jmath }= j^* \quad \text{ and } \quad \tau (\Pi ^{\hat{\jmath }})<T. \end{aligned}$$

Let α>0, $m\in \mathbb {N}^*$, $o\in \mathcal {O}$ the nature of object *m*, $j^*$ its category and ${{\boldsymbol{\Delta }}_\gamma }$ the constant given by Assumption 2.4. Let $\tilde{J} :=\{j\in J \text { such that } \gamma ^j_o >0\}$. According to Assumption 2.4, we have necessarily $j^*\in \tilde{J}$. Up to consider $\tilde{J}$ instead of J, let us assume $J = \tilde{J}$. For t≥0 and x≥0, let

C22 $$\begin{aligned} S(t) :=(\gamma ^{j^*}_o - {{\boldsymbol{\Delta }}_\gamma }) t + \sqrt{\frac{8}{3} \gamma ^{j^*}_o t\log \left( \frac{2|J |}{\alpha }\right) } \end{aligned}$$

and

C23 $$\begin{aligned} I(t) :=\gamma ^{j^*}_o t - \sqrt{\frac{8}{3} \gamma ^{j^*}_o t \log \left( \frac{2|J |}{\alpha }\right) }. \end{aligned}$$

Let $t_- :=\frac{8}{3}\log (\frac{2|J |}{\alpha })\max \left( \gamma _{\text {min}}^{-1}, 4\gamma ^{j^*}_o {{\boldsymbol{\Delta }}_\gamma }^{-2}\right)$ and $t_0 \in \left[ t_-,T \right]$ to choose later. Let Ω be the event

C24 $$\begin{aligned} \Omega :=\left\{ \forall j\in J, |\Pi ^j_{m,t_0} - \gamma ^j_o t_0 | < \sqrt{\frac{8}{3} t_0 \gamma ^j_o \log \left( \frac{2|J |}{\alpha }\right) } \right\} . \end{aligned}$$

Since the processes $(\Pi ^j_{m,t})_{t\ge 0}$ are Poisson processes with intensity $\gamma ^j_o>0$ and $t_0 \ge \frac{8}{3\gamma ^j_o}\log (\frac{2|J |}{\alpha })$ by lower bounding $\gamma ^j_o$ by $\gamma _{\text {min}}$, by union bound on the j∈J, according to inequality (C6) from Corollary C.6, the event Ω has probability more than 1-α. Let us work on Ω. We have

C25 $$\begin{aligned} \Pi ^{j^*}_{m,t_0} \ge \gamma ^{j^*}_o t_0 - \sqrt{\frac{8}{3} \gamma ^{j^*}_o t_0 \log \left( \frac{2|J |}{\alpha }\right) } = I(t_0) \end{aligned}$$

and for every $j\ne j^*$, by bounding $\gamma ^j_o$ by $\gamma ^{j^*}_o - {{\boldsymbol{\Delta }}_\gamma }$ and $\gamma ^{j^*}_o$ we have

C26 $$\begin{aligned}&\Pi ^j_{m,t_0} \le \gamma ^j_o t_0 + \sqrt{\frac{8}{3} \gamma ^j_o t_0 \log \left( \frac{2|J |}{\alpha }\right) } < (\gamma ^{j^*}_o - {{\boldsymbol{\Delta }}_\gamma }) t_0 \nonumber \\&\quad + \sqrt{\frac{8}{3} \gamma ^{j^*}_o t_0\log \left( \frac{2|J |}{\alpha }\right) } = S(t_0). \end{aligned}$$

thanks to Assumption 2.4. Let us study under which condition we have $S(t_0) < I(t_0)$. This is equivalent to the condition

C27 $$\begin{aligned} t_0 \ge \frac{32}{3{\boldsymbol{{\Delta }}}^2_\gamma } \gamma ^{j^*}_o \log \left( \frac{2|J |}{\alpha }\right) \end{aligned}$$

which we assumed. Suppose $\theta \in [I(t_-), I(T)]$. By continuity of t↦I(t), there exists $t_0\in [t_-,T]$ such that $I(t_0) =\theta$. Let us choose such $t_0$. Therefore, at time $t_0$, we have $\Pi ^{j^*}_{m,t_0}\ge \theta$ and for every $j\ne j^*$, $\Pi ^j_{m,t_0} < \theta$. This means that at time $t_0$, the process $(\Pi ^{j^*}_{m,t})_{t\ge 0}$ has already reached θ and since the processes $(\Pi ^j_{m,t})_{t\ge 0}$ are increasing, every other process $(\Pi ^j_{m,t})_{t\ge 0}$ where $j\ne j^*$ has not reached θ yet. Therefore, on Ω, under the condition $\theta \in [I(t_-), I(T)]$, the process $(\Pi ^{j^*}_{m,t})_{t\ge 0}$ reaches θ before limit duration *T* and is the first to do so. Since $I(t_-) \le \gamma ^{j^*}_o t_-$ by definition of the function *I*, sufficient conditions on θ are

C28 $$\begin{aligned} \frac{8}{3}\log \left( \frac{2|J |}{\alpha }\right) \max \left( \frac{\gamma ^{j^*}_o}{\gamma _{\text {min}}}, 4\left( \frac{\gamma ^{j^*}_o}{{{\boldsymbol{\Delta }}_\gamma }}\right) ^2\right) \le \theta \end{aligned}$$

and

C29 $$\begin{aligned} \theta \le \gamma ^{j^*}_o T - \sqrt{\frac{8}{3} \gamma ^{j^*}_o T \log \left( \frac{2|J |}{\alpha }\right) }. \end{aligned}$$

In order to get conditions independent of *o* and $j^*$, we upper bound $\gamma ^{j^*}_o$ by $\Vert \gamma \Vert _{\infty }$ and lower bound it by ${{\boldsymbol{\Delta }}_\gamma }$.

### C.4 Proof of Theorem 2.6

Let us prove the following result, from which derives Theorem 2.6.

#### Theorem C.12

Let $m\in \mathbb {N}^*$, $o\in \mathcal {O}$ the nature of the $m^{th}$ object and $n\in \mathbb {N}^*$. Let $\Pi ^n_m = (\Pi ^{j,n}_m)_{j\in J}$ a family of mutually independent Poisson processes with intensities $(n\gamma ^j_o)_{j\in J}$. Suppose the vector $\gamma _o$ verifies $\gamma ^j_o >0$ for every j∈J. Then there exist absolute constants a,b,d>0 and a |J|-dimensional drifted Brownian motion $W^n_m = (W^{j,n}_m)_{j\in J}$ defined by (1) such that for every x>0,

C30 $$\begin{aligned} \mathbb {P}\left( \sup _{j\in J, t\ge 0} \frac{|\Pi ^{j,n}_{m,t}-W^{j,n}_{m,t}|}{\log (n\gamma ^j_o t \vee 1+1)} \ge a+x\right) \le b|J | e^{-d x}. \end{aligned}$$

For j∈J, let us define the hitting times $\tau _n(\Pi ^j):=\inf \left\{ u\ge 0; \Pi ^{j,n}_{m,u} \ge n\theta \right\}$ and $\tau _n(W^j):=\inf \left\{ u\ge 0; W^{j,n}_{m,u} \ge n\theta \right\}$. Consequently, there exist absolute positive constants *C*, *K* and ζ such that for $\alpha \in (0,1)$, θ>0 and $n\ge \frac{2}{\gamma _{\text {min}}}\left( \frac{\theta }{\gamma _{\text {min}}}+1\right) \log \left( \frac{10|J |}{\alpha }\right)$, with probability more than 1-α, all the following inequalities hold jointly:

1. The processes $\Pi ^n_m$ and $W^n_m$ verify
  C31 $$\begin{aligned} \sup _{j\in J,t\ge 0} \frac{|\Pi ^{j,n}_{m,t}-W^{j,n}_{m,t}|}{\log (n\gamma ^j_o t \vee 1+1)} \le C +\zeta \log (5K|J |\alpha ^{-1}). \end{aligned}$$
2. The hitting times $(\tau _n(\Pi ^j))_{j\in J}$ verify
  C32 $$\begin{aligned} \sup _{j\in J} \left\lvert \tau _n(\Pi ^j)- \frac{\theta }{\gamma ^j_o} \right\rvert \le \sqrt{ \frac{8}{3n\gamma _{\text {min}}} \left( \frac{\theta }{\gamma _{\text {min}}} + 1\right) \log \left( \frac{10|J |}{\alpha }\right) } + \frac{1}{n\gamma _{\text {min}}}. \end{aligned}$$
3. The hitting times $(\tau _n(W^j))_{j\in J}$ verify
  C33 $$\begin{aligned} \sup _{j\in J}\left\lvert \tau _n(W^j)- \frac{\theta }{\gamma ^j_o} \right\rvert \le \sqrt{ \frac{2}{n\gamma _{\text {min}}}\left( \frac{\theta }{\gamma _{\text {min}}} + 1\right) \log \left( \frac{10|J |}{\alpha }\right) }. \end{aligned}$$
4. The difference between the hitting times $(\tau _n(\Pi ^j))_{j\in J}$ and $(\tau _n(W^j))_{j\in J}$ verifies
  C34 $$\begin{aligned} \sup _{j\in J}|\tau _n(\Pi ^j)- \tau _n(W^j) | \le \Box _{\theta ,\gamma _{\text {min}},\Vert \gamma \Vert _\infty } \frac{\log (n)}{n} \log \left( \frac{|J |}{\alpha }\right) . \end{aligned}$$

Let $m\in \mathbb {N}^*$, *o* the nature of object *m* and n≥1. Then (C30) is a direct consequence of Corollary C.4 by union bound on the j∈J and applying the time change $t\mapsto n\gamma ^j_o t$.

Let $$\alpha \in (0,1)$$ and $$U :=\max _{j\in J}\{\frac{\theta }{\gamma ^j_o}\} + 1 = \frac{\theta }{\gamma _{\text {min}}} + 1$$.

∙ Control of the difference $|\Pi ^{j,n}_{m,t} - W^{j,n}_{m,t} |$.

For each j∈J, let us apply Corollary C.4 to the Poisson process $(\Pi ^{j,n}_{m,t})_{t\ge 0}$ having intensity $n\gamma ^j_o$. By choosing uniform variables $V^j$ independent of everything else, there exist Brownian motions $W^{j,n}_{m,t}$ with drift $n\gamma ^j_o$ and scaling $\sqrt{n\gamma ^j_o}$ depending only on $(\Pi ^{j,n}_{m,t})_{t\ge 0}$ and $V^j$ such that the event

C35 $$\begin{aligned} \Omega _1 :=\left\{ \forall j\in J, \quad \sup _{t\ge 0} \frac{|\Pi ^{j,n}_{m,t}-W^{j,n}_{m,t}|}{\log (n\gamma ^j_o t \vee 1+1)} \le C +\zeta \log (5K|J |\alpha ^{-1}) \right\} \end{aligned}$$

has probability more than $1-\frac{\alpha }{5}$ by union bound, where *C*, *K* and ζ are positive constants independent of the parameters of the problem. Let us work on $\Omega _1$.

∙ Study of the hitting times of $W^{j,n}_{m,t}$ and $\Pi ^{j,n}_{m,t}$.

We introduce the first hitting times $\tilde{\tau }_n(\Pi ^j):=\inf \left\{ u\in [0,U] \text { s.t. } \Pi ^{j,n}_{m,u} \ge n\theta \right\}$ and $\tilde{\tau }_n(W^j)= \inf \left\{ u\in [0,U] \text { s.t. } W^{j,n}_{m,u} \ge n\theta \right\}$, and we set $\tilde{\tau }_n(\Pi ^j), \tilde{\tau }_n(W^j)= \infty$ if the previous sets are empty. First, let us study under which conditions $\tilde{\tau }_n(\Pi ^j)< \infty$ and $\tilde{\tau }_n(W^j)< \infty$.

Let $\Omega _2$ be the event

C36 $$\begin{aligned} \Omega _2 :=\left\{ \forall j\in J, \quad \Pi ^{j,n}_{m,U} \ge n\gamma ^j_o U - \sqrt{2n\gamma ^j_o U \log (5|J |\alpha ^{-1})}\right\} . \end{aligned}$$

Then $\Pi ^{j,n}_{m,U}$ is a Poisson variable with parameter $n\gamma ^j_o U$. Let us assume that for every j∈J we have $n\gamma ^j_o U \ge 2 \log (5|J |\alpha ^{-1})$. Then according to (C4) of Corollary C.6, by union bound $\mathbb {P}(\Omega _2) \ge 1 - \frac{\alpha }{5}$. Let us work on $\Omega _1 \cap \Omega _2$. Then a sufficient condition to have $\tilde{\tau }_n(\Pi ^j)< \infty$ is to have $n\gamma ^j_o U - \sqrt{2n\gamma ^j_o U \log (5|J |\alpha ^{-1})} \ge n\theta$, which is equivalent to the condition

C37 $$\begin{aligned} \frac{\theta }{\gamma ^j_o} \le U - \sqrt{\frac{2U}{n\gamma ^j_o} \log (5|J |\alpha ^{-1})}. \end{aligned}$$

which is fulfilled since we assumed that *n* verifies

C38 $$\begin{aligned} n \ge \frac{2}{\gamma _{\text {min}}} \left( \frac{\theta }{\gamma _{\text {min}}} + 1\right) \log \left( \frac{10|J |}{\alpha }\right) . \end{aligned}$$

Let $\Omega _3$ be the event

C39 $$\begin{aligned} \Omega _3 :=\left\{ \forall j\in J, \quad \sup _{0\le t \le U} |W^{j,n}_{m,t} - n\gamma ^j_o t | < \sqrt{2Un\gamma ^j_o \log (10|J |\alpha ^{-1})} \right\} \end{aligned}$$

Then according to Lemma C.1, we have $\mathbb {P}(\Omega _3) \ge 1-\frac{\alpha }{5}$. Let us work on $\Omega _1\cap \Omega _2\cap \Omega _3$. We have then

C40 $$\begin{aligned} W^{j,n}_{m,U} \ge n\gamma ^j_o U - \sqrt{2U n\gamma ^j_o\log (10|J |\alpha ^{-1})}. \end{aligned}$$

A sufficient condition to have $\tilde{\tau }_n(W^j)< \infty$ is to have $n\gamma ^j_o U - \sqrt{2Un\gamma ^j_o\log (10|J |\alpha ^{-1})}\ge n\theta$, which is fulfilled since *n* verifies (C38). Therefore, on $\Omega _1 \cap \Omega _2 \cap \Omega _3$, we have $\tilde{\tau }_n(\Pi ^j)< \infty , \tilde{\tau }_n(\Pi ^j)<\infty$ and consequently $\tau _n(\Pi ^j)= \tilde{\tau }_n(\Pi ^j)$ and $\tau _n(W^j)= \tilde{\tau }_n(W^j)$. Therefore, for every j∈J and $Z\in \{\Pi ,W\}$, we have $\tau _n(Z^j)\le U$, and controlling the trajectory of $Z^{j,n}_m$ on [0, *U*] is sufficient to control it on $[0,\tau _n(Z^j)]$.

Using this fact, let us study the convergence of $\tau _n(\Pi ^j)$ and $\tau _n(W^j)$ to $\frac{\theta }{\gamma ^j_o}$.

1. Convergence of $\tau _n(\Pi ^j)$. According to Corollary C.8, for $U \ge \frac{8}{3n\gamma ^j_o}\log (10|J |\alpha ^{-1})$, which holds for large *n*, the event

C41 $$\begin{aligned} \Omega _4 :=\left\{ \forall j\in J, \quad \sup _{0\le t \le U} |\Pi ^{j,n}_{m,t} - n\gamma ^j_o t | \le \sqrt{\frac{8}{3}n\gamma ^j_o U \log (10|J |\alpha ^{-1})} \right\} \end{aligned}$$

has probability more than $1-\frac{\alpha }{5}$. Let us work on $\Omega _1 \cap \Omega _2\cap \Omega _3\cap \Omega _4$. Since $\Pi ^{j,n}_{m,\tau _n(\Pi ^j)}$ is an integer and the process $(\Pi ^{j,n}_{m,t})_{t\ge 0}$ cannot make two jumps at the same time, we have $\Pi ^{j,n}_{m,\tau _n(\Pi ^j)} = \lceil n\theta \rceil$. Therefore, for every j∈J we have

C42 $$\begin{aligned}&|n\theta - n\gamma ^j_o \tau _n(\Pi ^j) | \le |n\theta - \lceil n\theta \rceil | + |\Pi ^{j,n}_{m,\tau _n(\Pi ^j)} - n \gamma ^j_o \tau _n(\Pi ^j) |\nonumber \\&\quad \le 1 + \sqrt{\frac{8}{3}n\gamma ^j_o U \log (10|J |\alpha ^{-1})} \end{aligned}$$

since we work on $\Omega _4$. Hence

$$\begin{aligned} \left\lvert \tau _n(\Pi ^j)- \frac{\theta }{\gamma ^j_o} \right\rvert&\le \frac{1}{n\gamma ^j_o} + \sqrt{\frac{8 U}{3 n\gamma ^j_o}\log (10|J |\alpha ^{-1})} . \end{aligned}$$

and we get the upper bound given by the theorem by lower bounding $\gamma ^j_o$ by $\gamma _{\text {min}}$ and replacing *U* by its value $\frac{\theta }{\gamma _{\text {min}}} + 1$.

2. Convergence of $\tau _n(W^j)$. Since we work on $\Omega _3$ we have

C43 $$\begin{aligned} |W^{j,n}_{m,\tau _n(W^j)} - n \gamma ^j_o \tau _n(W^j) | \le \sqrt{2Un\gamma ^j_o \log (10|J |\alpha ^{-1})}. \end{aligned}$$

Since the trajectories of $(W^{j,n}_{m,t})_{t\ge 0}$ are continuous, by definition of $\tau _n(W^j)$ we have $W^{j,n}_{m,\tau _n(W^j)} = n\theta$ so

C44 $$\begin{aligned} \left\lvert \tau _n(W^j)- \frac{\theta }{\gamma ^j_o} \right\rvert \le \sqrt{\frac{2U\log (10|J |\alpha ^{-1})}{n\gamma ^j_o}} \end{aligned}$$

and we again get the upper bound given by the theorem by lower bounding $\gamma ^j_o$ by $\gamma _{\text {min}}$ and replacing U by its value.

3. Study of the difference $|\tau _n(\Pi ^j)- \tau _n(W^j) |$. Let us begin by bounding $\left\lvert W^{j,n}_{m,\tau _n(\Pi ^j)} - W^{j,n}_{m,\tau _n(W^j)} \right\rvert$. We have

C45 $$\begin{aligned} \left\lvert W^{j,n}_{m,\tau _n(\Pi ^j)} - W^{j,n}_{m,\tau _n(W^j)} \right\rvert&= \left\lvert W^{j,n}_{m,\tau _n(\Pi ^j)} - n\theta \right\rvert \nonumber \\&\le \left\lvert W^{j,n}_{m,\tau _n(\Pi ^j)} - \lceil n\theta \rceil \right\rvert + | \lceil n\theta \rceil - n\theta | \nonumber \\&\le \left\lvert W^{j,n}_{m,\tau _n(\Pi ^j)} - \Pi ^{j,n}_{m,\tau _n(\Pi ^j)} \right\rvert + 1 \nonumber \\&\le \log (n\Vert \gamma \Vert _\infty U + 1) (C + \zeta \log (5K|J |\alpha ^{-1}) + 1 \nonumber \\&\le \Box _{\Vert \gamma \Vert _\infty , \theta , \gamma _{\text {min}}} \log (n)\log (|J |\alpha ^{-1}) \end{aligned}$$

since we are on $\Omega _1$ (C35) and since $\tau _n(\Pi ^j)$ is bounded by *U*.

Now, let us apply Lemma C.9 to the standard Brownian motion $B^j_t :=W^{j,n}_{m,(n\gamma ^j_o)^{-1} t} - t$. By union bound, the event

C46 $$\begin{aligned} \Omega _5 :=\left\{ \forall j\in J, \sup _{t, u \ge 0} \frac{|B^j_t-B^j_u|}{\sqrt{(|t-u|+2)[\log (t+1)+\log (u+1)+\frac{2}{c''}\log (5c'|J |\alpha ^{-1})]}} \le c\right\} \end{aligned}$$

has probability more than $1-\frac{\alpha }{5}$. Let us work on $\Omega _1\cap \Omega _2\cap \Omega _3\cap \Omega _4\cap \Omega _5$. Since $\tau _n(\Pi ^j)$ and $\tau _n(W^j)$ are bounded by *U* we have

$$\begin{aligned} |B^j_{n\gamma ^j_o \tau _n(\Pi ^j)} - B^j_{n\gamma ^j_o\tau _n(W^j)} | \le \Box _{\theta ,\gamma _{\text {min}},\Vert \gamma \Vert _\infty } \sqrt{((n |\tau _n(\Pi ^j)-\tau _n(W^j)|)\vee 1)\log (n|J |\alpha ^{-1})}. \end{aligned}$$

If $((n |\tau _n(\Pi ^j)-\tau _n(W^j)|)\vee 1) = 1$ then $|\tau _n(\Pi ^j)- \tau _n(W^j) | \le \frac{1}{n}$ and we have the result. Otherwise we have by definition of $B^j$

$$\begin{aligned}&\left\lvert W^{j,n}_{m,\tau _n(\Pi ^j)} - W^{j,n}_{m,\tau _n(\Pi ^j)} - n\gamma ^j_o(\tau _n(\Pi ^j)- \tau _n(W^j)) \right\rvert \\&\quad \le \Box _{\theta ,\gamma _{\text {min}},\Vert \gamma \Vert _\infty } \sqrt{n |\tau _n(\Pi ^j)-\tau _n(W^j)|\log (n|J |\alpha ^{-1})} \end{aligned}$$

thus

$$\begin{aligned}&\left\lvert W^{j,n}_{m,\tau _n(\Pi ^j)} - W^{j,n}_{m,\tau _n(\Pi ^j)} \right\rvert \\&\quad \ge n\gamma ^j_o|\tau _n(\Pi ^j)- \tau _n(W^j) | - \Box _{\theta ,\gamma _{\text {min}},\Vert \gamma \Vert _\infty } \sqrt{n |\tau _n(\Pi ^j)-\tau _n(W^j)|\log (n|J |\alpha ^{-1})}. \end{aligned}$$

Combining this with (C45), we obtain the following inequality:

C47 $$\begin{aligned} X^2 - a(n) X - b(n) \le 0 \end{aligned}$$

with $X :=\sqrt{n|\tau _n(\Pi ^j)- \tau _n(W^j) |}$, $a(n) :=\Box _{\theta ,\gamma _{\text {min}},\Vert \gamma \Vert _\infty } \sqrt{\log (n|J |\alpha ^{-1})}$ and $b(n):=\Box _{\Vert \gamma \Vert _\infty , \theta , \gamma _{\text {min}}} \log (n)\log (|J |\alpha ^{-1})$. This polynomial has two real roots, the largest one being $x_0 = \frac{a(n)+\sqrt{a(n)^2 + 4b(n)}}{2}$, so necessarily we have $X\le x_0$, which implies that $X\le a(n) + \sqrt{b(n)}$, *i.e., *

$$\begin{aligned} \sqrt{n|\tau _n(\Pi ^j)- \tau _n(W^j) |}&\le \Box _{\theta ,\gamma _{\text {min}},\Vert \gamma \Vert _\infty } \left( \sqrt{\log (n|J |\alpha ^{-1})} + \sqrt{ \log (n)\log (|J |\alpha ^{-1})} \right) \\&\le \Box _{\theta ,\gamma _{\text {min}},\Vert \gamma \Vert _\infty } \sqrt{ \log (n)\log (|J |\alpha ^{-1})} \end{aligned}$$

therefore

C48 $$\begin{aligned} |\tau _n(\Pi ^j)-\tau _n(W^j) | \le \Box _{\theta ,\gamma _{\text {min}},\Vert \gamma \Vert _\infty } \frac{\log (n)}{n} \log (|J |\alpha ^{-1}). \end{aligned}$$

To conclude, we note that $\mathbb {P}(\Omega _1\cap \Omega _2\cap \Omega _3\cap \Omega _4\cap \Omega _5)\ge 1-\alpha$.

### C.5 Proof of Proposition 3.3

Let us prove the following proposition from which Proposition 3.3 derives and which is more precise.

#### Proposition C.13

Let $\alpha \in (0,1)$ and $\gamma _{\text {min}} :=\min _{o\in \mathcal {O}, i\in I}\{\gamma ^i_o; \gamma ^i_o>0\}$. Suppose Assumption 3.2 holds and

C49 $$\begin{aligned} M T_{\text {min}} \ge \frac{8|\mathcal {O} |}{3\gamma _{\text {min}}} \log \left( \frac{2|I ||J |}{\alpha }\right) . \end{aligned}$$

For j∈J, let $w^j_\infty = (w^{i\rightarrow j}_\infty )_{i\in I}$ the weight family such that $w^{i\rightarrow j}_\infty :=\frac{1}{|I^j |}1\!\!1_{\{i\in I^j\}}$. Then with probability 1-α, at the end of the learning phase, the synaptic weights verify for all j∈J

$$\begin{aligned} \left\Vert w^j_{M+1} - w^j_\infty \right\Vert _2 \le 2 |\mathcal {O} |\eta _0&\sqrt{\frac{8 |I |}{3 T_{\text {min}}} \Vert \gamma \Vert _{\infty } \log (2|I ||J |\alpha ^{-1})} \\&+ 1\!\!1_{\{I^j \subsetneq I\}} \frac{\sqrt{|I |}}{|I^j |}\max \left( 1, \frac{|I | - |I^j |}{|I^j |} \right) \exp (- \eta _0\delta ^j \sqrt{M}), \end{aligned}$$

where $\eta _0$ is defined by (13).

To ensure clarity, we sometimes denote by $o_m$ the nature of object *m*. We will need the following lemma.

#### Lemma C.14

Let α>0 and $\gamma _{\text {min}} :=\min _{i\in I, o\in \mathcal {O}} \{ \gamma ^i_o \text { such that } \gamma ^i_o >0 \}$. Suppose Assumption 3.2 holds and that the following condition is fulfilled:

C50 $$\begin{aligned} M T_{\text {min}} \ge \frac{8}{3} \log \left( \frac{2|I ||J |}{\alpha }\right) \frac{|\mathcal {O} |}{\gamma _{\text {min}}}. \end{aligned}$$

Then with probability at least 1-α, for every i∈I and j∈J we have

$$\begin{aligned} \left\lvert \sum _{m \text { s.t. } o_m \in j} \Pi ^i_{m,T_{\text {min}}} - \frac{M T_{\text {min}}}{|\mathcal {O} |} \sum _{o\in j} \gamma ^i_o \right\rvert \le \sqrt{\frac{8 M T_{\text {min}}}{3|\mathcal {O} |} \sum _{o\in j} \gamma ^i_o \log (2|I ||J |\alpha ^{-1})}. \end{aligned}$$

#### Proof

Let β>0, j∈J, i∈I. Then by independence of the processes $((\Pi ^i_{m,t})_{t\ge 0})_{1\le m \le M}$, the variable $\sum _{m \text { s.t. } o_m \in j} \Pi ^i_{m,T_{\text {min}}}$ follows a Poisson distribution with parameter $\sum _{m \text { s.t. } o_m \in j} \gamma ^i_{o_m} T_{\text {min}}$. Besides, according to Assumption 3.2, we have

$$\begin{aligned} \sum _{m \text { s.t. } o_m \in j} \gamma ^i_{o_m}&= \sum _{o\in j} \sum _{m, o_m = o} \gamma ^i_o = \frac{M}{|\mathcal {O} |} \sum _{o\in j} \gamma ^i_o. \end{aligned}$$

Let $\Omega _{i,j}$ be the event

C51 $$\begin{aligned} \Omega _{i,j} :=\left\{ \left\lvert \sum _{m \text { s.t. } o_m \in j} \Pi ^i_{m,T_{\text {min}}} - \frac{M T_{\text {min}}}{|\mathcal {O} |} \sum _{o\in j} \gamma ^i_o \right\rvert \le \sqrt{\frac{8 M T_{\text {min}}}{3|\mathcal {O} |} \sum _{o\in j} \gamma ^i_o \log (2\beta ^{-1})} \right\} . \end{aligned}$$

If $\sum _{o\in j} \gamma ^i_o = 0$ then $\sum _{m \text { s.t. } o_m \in j} \Pi ^i_{m,T_{\text {min}}} = 0$ a.s. and $\mathbb {P}(\Omega _{i,j}) = 1$. Let us assume $\sum _{o\in j} \gamma ^i_o > 0$. Then according to inequality (C6) of Corollary C.6, under the condition

C52 $$\begin{aligned} \frac{M T_{\text {min}}}{|\mathcal {O} |} \sum _{o\in j} \gamma ^i_o \ge \frac{8}{3} \log (2\beta ^{-1}) \end{aligned}$$

we have $$\mathbb {P}(\Omega _{i,j})\ge 1-\beta $$.

Let us assume (C52) holds for every *i*, *j* such that $\sum _{o\in j} \gamma ^i_o > 0$. Then

$$\begin{aligned} \mathbb {P}(\cup _{i\in I, j\in J} \Omega _{i,j}^c)&\le |I ||J | \beta . \end{aligned}$$

Let us choose $\beta = \frac{\alpha }{|I ||J |}$. Then (C52) is implied by the condition

C53 $$\begin{aligned} M T_{\text {min}} \ge \frac{8}{3} \log \left( \frac{2|I ||J |}{\alpha }\right) \frac{|\mathcal {O} |}{\gamma _{\text {min}}} \end{aligned}$$

so under this condition $\mathbb {P}\left( \cap _{i\in I, j\in J} \Omega _{i,j}\right) \ge 1-\alpha$ and we have the result. □

Now let us prove the proposition. Let α>0 and suppose (C49) holds. Let us work on the event $\Omega :=\cap _{i\in I, j\in J} \Omega _{i,j}$ where the $\Omega _{i,j}$ are defined in the proof of Lemma C.14. Then according to Lemma C.14, $\mathbb {P}(\Omega )\ge 1-\alpha$. Let i∈I, j∈J and for $m\in \{1,\cdots ,M\}$ let

C54 $$\begin{aligned} \bar{g}^{i\rightarrow j}_m = \left\{ \begin{array}{lll} \gamma ^i_{o_m} \times \frac{M}{M^j} & \text {if } o \in j \\ \\ - \gamma ^i_{o_m} \!\times \! \frac{M}{M^{j'}} \!\times \! \frac{1}{|J |-1} & \text {if } o \in j'{\ne j} \end{array} \right. \end{aligned}$$

Let $\bar{G}^{i\rightarrow j}_M :=\sum _{m=1}^M \bar{g}^{i\rightarrow j}_m$ and let $\bar{w}^{i\rightarrow j}_{M+1} :=\dfrac{\exp (\eta \bar{G}^{i\rightarrow j}_M)}{\sum _{l\in I} \exp (\eta \bar{G}^{l\rightarrow j}_M)}$.

∙ Study of the difference $|G^{i\rightarrow j}_M - \bar{G}^{i\rightarrow j}_M |$.

According to Assumption 3.2 we have

$$\begin{aligned} \bar{G}^{i\rightarrow j}_M&= \frac{M}{M^j}\sum _{m \text { s.t. }o_m\in j} \gamma ^i_{o_m} - \frac{1}{|J |- 1} \sum _{j'\ne j} \frac{M}{M^{j'}} \sum _{m \text { s.t. }o_m\in j'} \gamma ^i_{o_m} \\&= \frac{M}{M^j} \frac{M}{|\mathcal {O} |} \sum _{o\in j} \gamma ^i_o - \frac{1}{|J |- 1} \sum _{j'\ne j} \frac{M}{M^{j'}} \frac{M}{|\mathcal {O} |} \sum _{o\in j'} \gamma ^i_o \end{aligned}$$

However, according to Assumption 3.2, for any j∈J we have

C55 $$\begin{aligned} M^j = n^j \times \frac{M}{|\mathcal {O} |}\end{aligned}$$

where $n^j$ is the number of object natures belonging to category *j*. Therefore we have

$$\begin{aligned} \bar{G}^{i\rightarrow j}_M&= M \left( \frac{1}{n^j} \sum _{o\in j} \gamma ^i_o - \frac{1}{|J |-1}\sum _{j'\ne j} \frac{1}{n^{j'}}\sum _{o\in j'} \gamma ^i_o \right) \\&= M d^{i\rightarrow j} \end{aligned}$$

and since we work on $\Omega _{i,j}$ we have, where *o* is the nature of object *m*:

$$\begin{aligned} |G^{i\rightarrow j}_M - \bar{G}^{i\rightarrow j}_M |&\le 2 \max _{j'\in J} \frac{|\mathcal {O} |}{n^{j'}} \left\lvert \sum _{m\text { s.t. } o_m \in j'} (\widehat{\gamma ^i_o} - \gamma ^i_o) \right\rvert \\&\le 2 |\mathcal {O} | \max _{j'\in J} \left\lvert \sum _{m\text { s.t. } o_m \in j'} \left( \frac{\Pi ^i_{m,T_{\text {min}}}}{T_{\text {min}}} - \gamma ^i_o\right) \right\rvert \\&\le 2 \sqrt{\frac{8 M |\mathcal {O} |}{3 T_{\text {min}}} \sum _{o\in j} \gamma ^i_o \log (2|I ||J |\alpha ^{-1})} \\&\le 2 |\mathcal {O} | \sqrt{\frac{8 M}{3 T_{\text {min}}} \Vert \gamma \Vert _{\infty } \log (2|I ||J |\alpha ^{-1})} \end{aligned}$$

where we bound $\frac{1}{n^{j'}}$ by 1 to go from the first line to the second, and we bound $\sum _{o\in j} \gamma ^i_o$ by $|\mathcal {O} |\Vert \gamma \Vert _{\infty }$ to go from the third line to the fourth.

∙ Study of the difference $\Vert \bar{w}^{j}_{M+1} - w^{j}_{M+1} \Vert _2$.

According to Gao and Pavel (2018) (Proposition 4), the softmax function defined on $\mathbb {R}^d$ with temperature η is η-Lipschitz, so we have

$$\begin{aligned} \Vert w^{j}_{M+1} - \bar{w}^{j}_{M+1} \Vert _2&\le \eta \sqrt{|I |} \times 2 |\mathcal {O} | \sqrt{\frac{8 M}{3 T_{\text {min}}} \Vert \gamma \Vert _{\infty } \log (2|I ||J |\alpha ^{-1})} \\&= 2 |\mathcal {O} |\eta _0 \sqrt{\frac{8 |I |}{3 T_{\text {min}}} \Vert \gamma \Vert _{\infty } \log (2|I ||J |\alpha ^{-1})} \end{aligned}$$

by replacing η by its value $\eta _0 M^{-1/2}$.

∙ Study of the difference $\Vert \bar{w}^{j}_{M+1} - w^{j}_\infty \Vert _2$.

We apply Proposition 30 of Jaffard et al. (2026). For j∈J, if $I^j = I$ then for every i∈I

C56 $$\begin{aligned} \bar{w}^{i\rightarrow j}_{M+1} = w^{i\rightarrow j}_\infty . \end{aligned}$$

Otherwise if $I^j\ne I$, we have

C57 $$\begin{aligned} |w^{i\rightarrow j}_\infty - \bar{w}^{i\rightarrow j}_{M+1} | \le \frac{1}{|I^j |}\max \left( 1, \frac{|I | - |I^j |}{|I^j |} \right) \exp (- \eta _0\delta ^j \sqrt{M}) \end{aligned}$$

and

C58 $$\begin{aligned} \Vert \bar{w}^{j}_{M+1} - w^{j}_\infty \Vert _2 \le \frac{\sqrt{|I |}}{|I^j |}\max \left( 1, \frac{|I | - |I^j |}{|I^j |} \right) \exp (- \eta _0\delta ^j \sqrt{M}). \end{aligned}$$

We get the result by combining the error terms.

### C.6 Proof of Theorem 3.5

Let us prove the following result from which Theorem 3.5 immediately derives.

#### Theorem C.15

Let $\alpha \in (0,1)$ and $\eta _0>0$, and $\mathbb {G} : t \mapsto \int _0^t \int _0^s g(s-u)du ds$. Let ${{\boldsymbol{\Delta }}_\lambda }>0$ and suppose Assumptions 3.2 and 3.4 (Margin (${{\boldsymbol{\Delta }}_\lambda }$)) hold. Let the parameters *M*, $T_{\text {min}}$, *T*, and θ verify

$$\begin{aligned} M\ge \Box \frac{1}{\eta _0^2 \delta _{\text {min}}^2} \log \left( \frac{|I |}{|I |_{\text {min}}} \right) ^2 + \log \left( \frac{\Vert \gamma \Vert _\infty }{{{\boldsymbol{\Delta }}_\lambda }}\right) ^2 \end{aligned}$$

where $$|I |_{\text {min}} :=\min _{j\in J} |I^j |$$ and $$\delta _{\text {min}} :=\min _{j\in J} \delta ^j$$,

$$\begin{aligned} T_{\text {min}} \ge \Box \eta _0^2 |\mathcal {O} |^2 |I |^2 \frac{\Vert \gamma \Vert _\infty ^3}{ {{\boldsymbol{\Delta }}_\lambda }^2} \log \left( \frac{|I ||J |}{\alpha }\right) , \end{aligned}$$

$$\begin{aligned} \mathbb {G}(T) > \Box _{\Vert g \Vert _1} \log \left( \frac{|J |}{\alpha }\right) \left( \frac{1}{\sqrt{{{\boldsymbol{\Delta }}_\lambda }}}\vee \frac{\Vert \gamma \Vert _{\infty }}{{{\boldsymbol{\Delta }}_\lambda }}\right) , \end{aligned}$$

$$\begin{aligned} \theta \ge \left( \Box _{\Vert g \Vert _1} \log \left( \frac{|J |}{\alpha }\right) \left( \frac{\Vert \gamma \Vert _\infty }{\sqrt{{{\boldsymbol{\Delta }}_\lambda }}}\vee \frac{\Vert \gamma \Vert _{\infty }^2}{{{\boldsymbol{\Delta }}_\lambda }}\right) \right) \vee \Vert \gamma \Vert _\infty \mathbb {G}(T_{\text {min}}) \end{aligned}$$

and

$$\begin{aligned} \theta < \Box {{\boldsymbol{\Delta }}_\lambda }\mathbb {G}(T) - \Box _{\Vert \gamma \Vert _\infty ,\Vert g \Vert _1} \left( \sqrt{ \mathbb {G}(T)\log \left( \frac{|J |}{\alpha }\right) } + \log \left( \frac{|J |}{\alpha }\right) \right) . \end{aligned}$$

Then with probability larger than 1-α, we have that for all nature $o\in \mathcal {O}$ of the object presented after the learning phase, that is the M+1 object, if we denote by $j^*$ its category, the choice $\hat{\jmath }$ (10) and the reaction time $\tau (N^{\hat{\jmath }})$ (9) satisfy

C59 $$\begin{aligned} \hat{\jmath }= j^* \quad \text{ and } \quad \tau (N^{\hat{\jmath }}) < T. \end{aligned}$$

We will need several lemmas. Let $\Lambda ^{j}_{m,t} :=\int _0^t \lambda ^{j}_{m,s}ds$ be the compensator of the process $(N^{j}_{m,t})_{t\ge 0}$. We denote $\bar{\Lambda }^j_{m,t} :=\mathbb {E}[\Lambda ^j_{m,t} \mid \mathcal {F}_{m-1}]$.

#### Lemma C.16

Let x>0, $m\in \{1,\cdots ,M+1\}$, j∈J, t≥0. Then we have

C60 $$\begin{aligned} \mathbb {P}\left( \Lambda ^j_{m,t} - \bar{\Lambda }^j_{m,t} \ge \Vert g \Vert _1 \frac{x}{3} + \sqrt{2x\Vert g \Vert _1 \bar{\Lambda }^j_{m,t} } \mid \mathcal {F}_{m-1}\right) \le e^{-x} \end{aligned}$$

and

C61 $$\begin{aligned} \mathbb {P}\left( \bar{\Lambda }^j_{m,t} - \Lambda ^j_{m,t} \ge \Vert g \Vert _1 \frac{x}{3} + \sqrt{2x\Vert g \Vert _1 \bar{\Lambda }^j_{m,t} } \mid \mathcal {F}_{m-1}\right) \le e^{-x}. \end{aligned}$$

#### Proof

Let x>0. Let us use the Cramér-Chernoff method. Note that the weights $w^j_m$ are $\mathcal {F}_{m-1}-$measurable. Let $h_t(u) :=\int _u^t g(s-u) ds$, ξ>0. Then according to Fubini-Tonelli theorem, since *g* is positive and the random measures defined by the processes $(\Pi ^i_{m,t})_{t\ge 0}$ are σ-finite we have almost surely

$$\begin{aligned} \Lambda ^j_{m,t}&=\sum _{i\in I} w^{i\rightarrow j}_m \int _{s=0}^t \int _{u = 0}^s g(s-u)d\Pi ^i_{m,u}ds \\&= \sum _{i\in I} w^{i\rightarrow j}_m \int _{u=0}^t\int _{s=u}^t g(s-u) ds d\Pi ^i_{m,u} \\&= \sum _{i\in I} w^{i\rightarrow j}_m \int _{u=0}^t h_t(u) d\Pi ^i_{m,u}. \end{aligned}$$

Therefore

C62 $$\begin{aligned} \mathbb {E}[e^{\xi \Lambda ^j_{m,t}}\mid \mathcal {F}_{m-1}] = \mathbb {E}\left[ \exp \left( \sum _{i\in I} w^{i\rightarrow j}_m \xi \int _{u=0}^t h_t(u) d\Pi ^i_{m,u} \right) \mid \mathcal {F}_{m-1}\right] \end{aligned}$$

and by independence of the processes $((\Pi ^i_{m,t})_{t\ge 0})_{i\in I}$ we have

C63 $$\begin{aligned} \mathbb {E}[e^{\xi \Lambda ^j_{m,t}}\mid \mathcal {F}_{m-1}] = \prod _{i\in I}\mathbb {E}\left[ \exp \left( w^{i\rightarrow j}_m \xi \int _{u=0}^t h_t(u) d\Pi ^i_{m,u} \right) \mid \mathcal {F}_{m-1}\right] . \end{aligned}$$

Besides, according to Proposition 6 of Reynaud-Bouret (2003),

$$\begin{aligned} \mathbb {E}[e^{\xi \Lambda ^j_{m,t}} \mid \mathcal {F}_{m-1}]&= \prod _{i\in I} \exp \left( \gamma ^i_o \int _0^t(e^{\xi w^{i\rightarrow j}_m h_t(u)}- 1)du \right) \\&= \exp \left( \sum _{i\in I} \gamma ^i_o \int _0^t(e^{\xi w^{i\rightarrow j}_m h_t(u)}- 1)du \right) \\&= \exp \left( \sum _{i\in I} \gamma ^i_o \int _0^t \sum _{k=1}^\infty \frac{(\xi w^{i\rightarrow j}_m h_t(u))^k}{k!} du \right) \\&= \exp (\xi \bar{\Lambda }^j_{m,t})\exp \left( \sum _{i\in I} \gamma ^i_o \int _0^t \sum _{k=2}^\infty \frac{(\xi w^{i\rightarrow j}_m h_t(u))^k}{k!} du \right) \\&\le \exp (\xi \bar{\Lambda }^j_{m,t})\exp \left( \bar{\lambda }^j_m \mathbb {G}(t)\sum _{k=2}^\infty \frac{\xi ^k\Vert g \Vert _1^{k-1}}{k!} \right) \end{aligned}$$

where we exchanged the sum and integral because the series of functions converges uniformly, we bounded $h_t(u)$ by $\Vert g \Vert _1$ and $(w^{i\rightarrow j}_m)^k$ by $w^{i\rightarrow j}_m$ (because $w^{i\rightarrow j}_m$ is bounded by 1).

Suppose $\xi \Vert g \Vert _1 < 1$. Since $\bar{\lambda }^j_m \mathbb {G}(t) = \bar{\Lambda }^j_{m,t}$ we have

$$\begin{aligned} \mathbb {E}[e^{\xi (\Lambda ^j_{m,t} - \bar{\Lambda }^j_{m,t})}\mid \mathcal {F}_{m-1}]&\le \exp \left( \bar{\Lambda }^j_{m,t} \frac{\xi ^2\Vert g \Vert _1}{2}\sum _{k=1}^\infty \frac{(\xi \Vert g \Vert _1)^{k-2}}{3^{k-2}}\right) \\&= \exp \left( \bar{\Lambda }^j_{m,t} \frac{\xi ^2\Vert g \Vert _1}{2} \frac{1}{1 - \frac{\xi \Vert g \Vert _1}{3}}\right) . \end{aligned}$$

By applying Markov’s inequality to the variable $e^{\xi (\Lambda ^j_{m,t} - \bar{\Lambda }^j_{m,t})}$ conditionally to the σ-algebra $\mathcal {F}_{m-1}$, we get

$$\begin{aligned} \mathbb {P}\left( \Lambda ^j_{m,t} - \bar{\Lambda }^j_{m,t} \ge x \mid \mathcal {F}_{m-1}\right)&\le e^{-\xi x} \exp \left( \bar{\Lambda }^j_{m,t} \frac{\xi ^2\Vert g \Vert _1}{2} \frac{1}{1 - \frac{\xi \Vert g \Vert _1}{3}}\right) \\&= \exp \left( -\xi x + \frac{ \bar{\Lambda }^j_{m,t} \xi ^2\Vert g \Vert _1 }{2\left( 1 - \frac{\xi \Vert g \Vert _1}{3}\right) }\right) . \end{aligned}$$

Then similarly as in section 2.4 of Boucheron et al. (2013), we have

C64 $$\begin{aligned} \sup _{\xi \in (0,3\Vert g \Vert _1^{-1})} \left\{ \xi x - \frac{\bar{\Lambda }^j_{m,t} \xi ^2\Vert g \Vert _1}{2\left( 1 - \frac{\xi \Vert g \Vert _1}{3}\right) } \right\} = \frac{9}{\Vert g \Vert _1} \bar{\Lambda }^j_{m,t} f\left( \frac{x}{3 \bar{\Lambda }^j_{m,t} } \right) \end{aligned}$$

where $$f : v \mapsto 1 + v - \sqrt{1+2v}$$. Therefore

$$\begin{aligned} \mathbb {P}\left( \Lambda ^j_{m,t} - \bar{\Lambda }^j_{m,t} \ge x \mid \mathcal {F}_{m-1}\right) \le \exp \left( -\frac{9}{\Vert g \Vert _1} \bar{\Lambda }^j_{m,t} f\left( \frac{x}{3 \bar{\Lambda }^j_{m,t} } \right) \right) . \end{aligned}$$

Besides, *f* is an increasing function from $\mathbb {R}^*_+$ to $\mathbb {R}^*_+$ with inverse $f^{-1} : v \mapsto v + \sqrt{2v}$ so we have

C65 $$\begin{aligned} \mathbb {P}\left( \Lambda ^j_{m,t} - \bar{\Lambda }^j_{m,t} \ge 3 \bar{\Lambda }^j_{m,t} f^{-1}\left( \frac{\Vert g \Vert _1 x}{9\bar{\Lambda }^j_{m,t}}\right) \mid \mathcal {F}_{m-1}\right) \le e^{-x} \end{aligned}$$

and by integrating we get (C60). Now, let us prove (C61). Let us consider $-\Lambda ^j_{m,t}$ instead of $\Lambda ^j_{m,t}$. Then similarly we have

C66 $$\begin{aligned} \mathbb {E}[e^{ -\xi \Lambda ^j_{m,t}}\mid \mathcal {F}_{m-1}] \le \exp (-\xi \bar{\Lambda }^j_{m,t})\exp \left( \bar{\lambda }^j_m \mathbb {G}(t)\sum _{k=2}^\infty \frac{\xi ^k\Vert g \Vert _1^{k-1}}{k!} du \right) \end{aligned}$$

hence

C67 $$\begin{aligned} \mathbb {E}[e^{ \xi ( \bar{\Lambda }^j_{m,t} - \Lambda ^j_{m,t} )}\mid \mathcal {F}_{m-1}] \le \exp \left( \bar{\lambda }^j_m \mathbb {G}(t)\sum _{k=2}^\infty \frac{\xi ^k\Vert g \Vert _1^{k-1}}{k!} du \right) \end{aligned}$$

so the same computations apply to bound $\mathbb {E}[e^{ \xi ( \bar{\Lambda }^j_{m,t} - \Lambda ^j_{m,t} )}\mid \mathcal {F}_{m-1}]$, and by applying Markov’s inequality to the variable $e^{ \xi ( \bar{\Lambda }^j_{m,t} - \Lambda ^j_{m,t} )}$ we get the same bound as in (C60) for (C61).

Now that we proved (C60) and (C61), we can combine them to have the result. □

#### Lemma C.17

Let x>0, $m\in \{1,\cdots ,M+1\}$, j∈J, t≥0. Let $e(x,t):=\Vert g \Vert _1 \frac{x}{3} + \sqrt{2x\Vert g \Vert _1 \bar{\Lambda }^j_{m,t} }$. Then we have

C68 $$\begin{aligned} \mathbb {P}\left( N^j_{m,t} - \bar{\Lambda }^j_{m,t} \le \sqrt{2( \bar{\Lambda }^j_{m,t} + e(x,t))x} + \frac{x}{3} + e(x,t) \right) \ge 1 - 2 e^{-x} \end{aligned}$$

and

C69 $$\begin{aligned} \mathbb {P}\left( \bar{\Lambda }^j_{m,t} - N^j_{m,t} \le \sqrt{2( \bar{\Lambda }^j_{m,t} + e(x,t)) x} + \frac{x}{3} + e(x,t) \right) \ge 1 - 3e^{-x}. \end{aligned}$$

#### Proof

Let x>0, j∈J, t≥0. Let us prove (C68). Let us work conditionally to $\mathcal {F}_{m-1}$. Let us use inequality (3.3) of Hansen et al. (2015) with $N = (N^l_{m,t})_{l\in I\cup J}$, $H = (H^l)_{l\in I\cup J}$ with $H^j \equiv 1$ and $H^l \equiv 0$ if l≠j, and B=1. Let $\rho \in \mathbb {R}$ to choose later. Then we have

C70 $$\begin{aligned} \mathbb {P}\left( N^j_{m,t} - \Lambda ^j_{m,t} \ge \sqrt{2\rho x} + \frac{x}{3} \ \text {and} \ \Lambda ^j_{m,t} \le \rho \mid \mathcal {F}_{m-1}\right) \le e^{-x}. \end{aligned}$$

Let us choose $\rho :=\bar{\Lambda }^j_{m,t} + e(x,t)$ which is $\mathcal {F}_{m-1}$-measurable. Let $\Omega _1$ be the event

C71 $$\begin{aligned} \Omega _1 :=\left\{ N^j_{m,t} - \Lambda ^j_{m,t} \ge \sqrt{2\rho x} + \frac{x}{3} \right\} \end{aligned}$$

and $\Omega _2$ be the event

C72 $$\begin{aligned} \Omega _2 :=\left\{ \Lambda ^j_{m,t} \le \rho \right\} . \end{aligned}$$

Then $\mathbb {P}(\Omega _1 \cap \Omega _2^c)\le e^{-x}$ and according to Lemma C.16 inequality (C60), $\mathbb {P}(\Omega _2^c)\le e^{-x}$. Besides,

C73 $$\begin{aligned} \Omega _1^c \cap \Omega _2 \subset \left\{ N^j_{m,t} - \bar{\Lambda }^j_{m,t} \le \sqrt{2\rho x} + \frac{x}{3} + e(x,t) \right\} \end{aligned}$$

and by integrating we get

C74 $$\begin{aligned} \mathbb {P}(\Omega _1 \cup \Omega _2^c) = \mathbb {P}(\Omega _1 \cap \Omega _2) + \mathbb {P}(\Omega _2^c) \le 2e^{-x} \end{aligned}$$

therefore

C75 $$\begin{aligned} \mathbb {P}\left( N^j_{m,t} - \bar{\Lambda }^j_{m,t} \le \sqrt{2\rho x} + \frac{x}{3} + e(x,t) \right) \ge \mathbb {P}(\Omega _1^c \cap \Omega _2) \ge 1 - 2 e^{-x} \end{aligned}$$

By replacing ρ by its value we get (C68).

Now, let us prove (C69). Similarly, let us use inequality (3.3) of Hansen et al. (2015) with $H^j \equiv -1$ and $H^l \equiv 0$ if l≠j. Let $\rho \in \mathbb {R}$ to choose later. Then we get

C76 $$\begin{aligned} \mathbb {P}\left( \Lambda ^j_{m,t} - N^j_{m,t} \ge \sqrt{2\rho x} + \frac{x}{3} \ \text {and} \ \Lambda ^j_{m,t} \le \rho \mid \mathcal {F}_{m-1}\right) \le e^{-x}. \end{aligned}$$

Let us choose the same ρ as before: $\rho :=\bar{\Lambda }^j_{m,t} + e(x,t)$. Let $\Omega _3$ be the event

C77 $$\begin{aligned} \Omega _3 :=\left\{ \Lambda ^j_{m,t} - N^j_{m,t} \ge \sqrt{2\rho x} + \frac{x}{3} \right\} \end{aligned}$$

and $\Omega _4$ be the event

C78 $$\begin{aligned} \Omega _4 :=\left\{ \bar{\Lambda }^j_{m,t} -\Lambda ^j_{m,t} \ge e(x,t)\right\} . \end{aligned}$$

Then $\mathbb {P}(\Omega _3 \cap \Omega _2)\le e^{-x}$ and according to Lemma (C.16) inequality (C61), $\mathbb {P}(\Omega _4^c)\le e^{-x}$. Besides,

C79 $$\begin{aligned} \Omega _3^c\cap \Omega _2 \cap \Omega _4^c \subset \Omega _3^c\cap \Omega _4^c \subset \left\{ \bar{\Lambda }^j_{m,t} - N^j_{m,t} \le \sqrt{2\rho x} + \frac{x}{3} + e(x,t) \right\} \end{aligned}$$

and by integrating we have

C80 $$\begin{aligned} \mathbb {P}(\Omega _3\cup \Omega _2^c \cup \Omega _4) \le \mathbb {P}(\Omega _4) + \mathbb {P}(\Omega _3 \cap \Omega _2) + \mathbb {P}(\Omega _2^c) \le 3e^{-x} \end{aligned}$$

so we have

C81 $$\begin{aligned} \mathbb {P}\left( \bar{\Lambda }^j_{m,t} - N^j_{m,t} \le \sqrt{2\rho x} + \frac{x}{3} + e(x,t) \right) \ge 1 - 3e^{-x}. \end{aligned}$$

By replacing ρ by its value we get (C69). □

#### Lemma C.18

Let $m\in \{1,\cdots ,M+1\}$, t≥0, α>0. Then with probability more than 1-α, we have

$$\begin{aligned} \forall j\in J, \quad \left\lvert N^j_{m,t} - \bar{\Lambda }^j_{m,t} \right\rvert \le \Box \left( \sqrt{\bar{\Lambda }^j_{m,t}(\Vert g \Vert _1 \vee 1) \log \left( \frac{|J |}{\alpha }\right) } + (\Vert g \Vert _1 \vee 1) \log \left( \frac{|J |}{\alpha }\right) \right) . \end{aligned}$$

#### Proof

By combining inequalities (C68) and (C69) of Lemma C.17, we get

$$\begin{aligned} \mathbb {P}\left( \left\lvert N^j_{m,t} - \bar{\Lambda }^j_{m,t} \right\rvert \le \sqrt{2( \bar{\Lambda }^j_{m,t} + e(x,t))x} + \frac{x}{3} + e(x,t) \right) \ge 1 - 5 e^{-x}. \end{aligned}$$

Let us choose $x_\alpha = \log (5|J |\alpha ^{-1})$, such that by union bound

$$\begin{aligned} \mathbb {P}\left( \forall j\in J, \left\lvert N^j_{m,t} - \bar{\Lambda }^j_{m,t} \right\rvert \le \sqrt{2( \bar{\Lambda }^j_{m,t} + e(x_\alpha ,t))x_\alpha } + \frac{x_\alpha }{3} + e(x_\alpha ,t) \right) \ge 1 - \alpha \end{aligned}$$

which concludes the proof.

□

#### Lemma C.19

Let α>0, $t\in [T_{\text {min}},T]$ and $\eta = \frac{\eta _0}{\sqrt{M}}$ where $\eta _0>0$. Suppose Assumption 3.2 holds. Let $\bar{\Lambda }^j_{\infty ,t} :=\sum _{i\in I} w^{i\rightarrow j}_\infty \gamma ^i_o \mathbb {G}(t)$. Then there exists an absolute constant □>0 such that with probability more than 1-α, for every j∈J we have

C82 $$\begin{aligned} \left\lvert \bar{\Lambda }^j_{M+1,t} - \bar{\Lambda }^j_{\infty ,t} \right\rvert \le \Box \bar{B}(\alpha )\sqrt{|I |} \Vert \gamma \Vert _{\infty } \mathbb {G}(t) \end{aligned}$$

where

C83 $$\begin{aligned} \bar{B}(\alpha ):=|\mathcal {O} |\eta _0 \sqrt{\frac{|I |}{ T_{\text {min}}} \Vert \gamma \Vert _{\infty } \log \left( \frac{|I ||J |}{\alpha }\right) } + \frac{|I |^{3/2}}{|I |_{\text {min}}} e^{-\eta _0 \delta _{\text {min}}\sqrt{M}} \end{aligned}$$

with $$|I |_{\text {min}} :=\min _{j\in J} |I^j |$$ and $$\delta _{\text {min}} :=\min _{j\in J} \delta ^j$$.

#### Proof

Let α>0, $t\in [T_{\text {min}},T]$, j∈J. Then according to Proposition 3.3, there exists an event Ω with probability more than 1-α such that on Ω, for every j∈J we have

$$\begin{aligned} \left\Vert w^j_{M+1} - w^j_\infty \right\Vert _2&\le 2 |\mathcal {O} |\eta _0 \sqrt{\frac{8 |I |}{3 T_{\text {min}}} \Vert \gamma \Vert _{\infty } \log (2|I ||J |\alpha ^{-1})} \\&\hspace{1cm} + 1\!\!1_{\{I^j \subsetneq I\}} \frac{\sqrt{|I |}}{|I^j |}\max \left( 1, \frac{|I | - |I^j |}{|I^j |} \right) \exp (- \eta _0\delta ^j \sqrt{M}) \\&\le \Box \left( |\mathcal {O} |\eta _0 \sqrt{\frac{|I |}{ T_{\text {min}}} \Vert \gamma \Vert _{\infty } \log \left( \frac{|I ||J |}{\alpha }\right) } + \frac{|I |^{3/2}}{|I |_{\text {min}}} e^{-\eta _0 \delta _{\text {min}}\sqrt{M}} \right) \\&= \Box \bar{B}(\alpha ) \end{aligned}$$

Let us work on Ω. We have

$$\begin{aligned} \left\lvert \bar{\Lambda }^j_{M+1,t} - \bar{\Lambda }^j_{\infty ,t} \right\rvert&= \left\lvert \sum _{i\in I} (w^{i\rightarrow j}_{M+1} - w^{i\rightarrow j}_\infty ) \gamma ^i_o \mathbb {G}(t) \right\rvert \\&\le \Vert w^j_{M+1} - w^j_\infty \Vert _2 \sqrt{|I |} \Vert \gamma \Vert _{\infty } \mathbb {G}(t) \end{aligned}$$

which concludes the proof. □

#### Lemma C.20

Let α>0 and $\eta = \frac{\eta _0}{\sqrt{M}}$ where $\eta _0>0$. Then with probability more than 1-α, for every j∈J we have

$$\begin{aligned}&\left\lvert N^j_{M+1,t}- \bar{\Lambda }^j_{\infty ,t} \right\rvert \le \Theta ^j_t \end{aligned}$$

where

$$\begin{aligned} \Theta ^j_t :=D \left( \bar{B}\left( \frac{\alpha }{2}\right) \sqrt{|I |} \Vert \gamma \Vert _{\infty } \mathbb {G}(t) + \sqrt{\bar{\Lambda }^j_{m,t}(\Vert g \Vert _1 \vee 1) \log \left( \frac{2|J |}{\alpha }\right) } \right. \\ \left. + (\Vert g \Vert _1 \vee 1) \log \left( \frac{2|J |}{\alpha }\right) \right) \end{aligned}$$

where D>0 is an absolute constant.

#### Proof

Let α>0, $t\in [T_{\text {min}},T]$. By combining the results of Lemmas C.18 and C.19 for every j∈J with $\frac{\alpha }{2}$ instead of α we have the result. □

Now we can prove the theorem. Let $\alpha \in (0,1)$, $t_0\in [T_{\text {min}},T]$ to choose later. Let us work on the event defined in the proof of Lemma C.20 for $t=t_0$. Let

C84 $$\begin{aligned} I(t_0) :=\bar{\Lambda }^{j^*}_{\infty ,t_0} - \Theta ^{j^*}_{t_0}, \end{aligned}$$

and for j∈J,

C85 $$\begin{aligned} S^j(t_0) :=\bar{\Lambda }^j_{\infty ,t_0} + \Theta ^j_{t_0}. \end{aligned}$$

such that

C86 $$\begin{aligned} N^{j^*}_{M+1,t_0} > I(t_0) \end{aligned}$$

and for every j∈J,

C87 $$\begin{aligned} N^j_{M+1,t_0} < S^j(t_0). \end{aligned}$$

We would like to choose $t_0$ such that for every $j\ne j^*$, $S^j(t_0) \le I(t_0)$ and $I(t_0) = \theta$. It would imply that at time $t_0$, $N^{j^*}_{M+1}$ has already reached θ and for every $j\ne j^*$, $N^j_{M+1}$ has not reached θ yet. The condition $S^j(t_0) \le I(t_0)$ is implied by

C88 $$\begin{aligned} \bar{\Lambda }^{j^*}_{\infty ,t_0} - \bar{\Lambda }^j_{\infty ,t_0} \ge \Theta ^{j^*}_{t_0} + \Theta ^{j}_{t_0} \end{aligned}$$

which is implied by

$$\begin{aligned}&{{\boldsymbol{\Delta }}_\lambda }\mathbb {G}(t_0) \ge 2 \Theta ^{j^*}_{t_0}. \end{aligned}$$

Let $R :={{\boldsymbol{\Delta }}_\lambda }- 2D\bar{B}\left( \frac{\alpha }{2}\right) \sqrt{|I |} \Vert \gamma \Vert _{\infty }$. Suppose $R \ge \frac{{{\boldsymbol{\Delta }}_\lambda }}{2}$. Then a sufficient condition is

C89 $$\begin{aligned} R\mathbb {G}(t_0) \ge 2D \left( \sqrt{\bar{\lambda }^{j^*}_{M+1}\mathbb {G}(t_0)(\Vert g \Vert _1 \vee 1) \log \left( \frac{2|J |}{\alpha }\right) } + (\Vert g \Vert _1 \vee 1) \log \left( \frac{2|J |}{\alpha }\right) \right) . \end{aligned}$$

This inequality has the form $aX^2 - bX - c \ge 0$ with $X = \sqrt{\mathbb {G}(t_0)}$, and *a*, *b*, *c* positive constants. The polynomial has two real roots, the largest one being $\frac{b+\sqrt{b^2 + 4ac}}{2a}$, so a sufficient condition is $X \ge \frac{b}{a} + \sqrt{\frac{c}{a}}$, *i.e., *

C90 $$\begin{aligned} \mathbb {G}(t_0) \ge \Box (\Vert g \Vert _1 \vee 1) \log \left( \frac{2|J |}{\alpha }\right) \left( R^{-1/2} + \Vert \gamma \Vert _{\infty } R^{-1} \right) ^2. \end{aligned}$$

Let $t_-$ the minimal $t\ge T_{\text {min}}$ satisfying (C90), such that

C91 $$\begin{aligned} \mathbb {G}(t_-) = \left( \Box (\Vert g \Vert _1 \vee 1) \log \left( \frac{2|J |}{\alpha }\right) \left( R^{-1/2} + \Vert \gamma \Vert _{\infty } R^{-1}\right) ^2\right) \vee \mathbb {G}(T_{\text {min}}). \end{aligned}$$

Since $\mathbb {G}$ tends to infinity, it is possible to choose such $t_-$. Since $\mathbb {G}$ is increasing, a sufficient condition to have $t_-< T$ is $\mathbb {G}(t_-) < \mathbb {G}(T)$. Then if $\theta \in [I(t_-),I(T))$, by continuity of *I* there exists $t_0\in [t_-,T)$ such that $I(t_0)=\theta$, and we have the result. Let us now explicit the conditions we had to assume.

We have

$$\begin{aligned}&R \ge \frac{{{\boldsymbol{\Delta }}_\lambda }}{2} \\ \Leftrightarrow \quad&{{\boldsymbol{\Delta }}_\lambda }- 2D\bar{B}\left( \frac{\alpha }{2}\right) \sqrt{|I |} \Vert \gamma \Vert _{\infty }\ge \frac{{{\boldsymbol{\Delta }}_\lambda }}{2} \\ \Leftarrow \quad&\bar{B}\left( \frac{\alpha }{2}\right) \le \Box \frac{{{\boldsymbol{\Delta }}_\lambda }}{\sqrt{|I |}\Vert \gamma \Vert _\infty } \\ \Leftrightarrow \quad&|\mathcal {O} |\eta _0 \sqrt{\frac{|I |}{ T_{\text {min}}} \Vert \gamma \Vert _{\infty } \log \left( \frac{2|I ||J |}{\alpha }\right) } + \frac{|I |^{3/2}}{|I |_{\text {min}}} e^{-\eta _0 \delta _{\text {min}}\sqrt{M}} \le \Box \frac{{{\boldsymbol{\Delta }}_\lambda }}{\sqrt{|I |}\Vert \gamma \Vert _\infty } \end{aligned}$$

A sufficient condition is that both terms of the left side of the inequality are less than half the right term, which reads as

C92 $$\begin{aligned} T_{\text {min}} \ge \Box {{\boldsymbol{\Delta }}_\lambda }^{-2}|\mathcal {O} |^2 \eta _0^2 |I |^2\Vert \gamma \Vert _\infty ^3 \log \left( \frac{|I ||J |}{\alpha }\right) \end{aligned}$$

and

C93 $$\begin{aligned} M\ge \Box \frac{1}{\eta _0^2 \delta _{\text {min}}^2} \log \left( \frac{|I |}{|I |_{\text {min}}} \right) ^2 + \log \left( \frac{\Vert \gamma \Vert _\infty }{{{\boldsymbol{\Delta }}_\lambda }}\right) ^2. \end{aligned}$$

Besides,

$$\begin{aligned}&\theta \le I(T) \\ \Leftrightarrow \quad&\theta \le \left( \bar{\lambda }^j_{\infty } - D \bar{B}\left( \frac{\alpha }{2}\right) \sqrt{|I |} \Vert \gamma \Vert _{\infty }\right) \mathbb {G}(T) \\&\hspace{2cm} - D \sqrt{\bar{\lambda }^{j^*}_{\infty }\mathbb {G}(T)(\Vert g \Vert _1 \vee 1) \log \left( \frac{2|J |}{\alpha }\right) } - D (\Vert g \Vert _1 \vee 1) \log \left( \frac{2|J |}{\alpha }\right) \end{aligned}$$

where $\bar{\lambda }^{j^*}_{\infty } :=\sum _{i\in I}w^{i\rightarrow j^*}_\infty \gamma ^i_o$. Since $\bar{\lambda }^{j^*}_{\infty } \ge {{\boldsymbol{\Delta }}_\lambda }$ and $R\ge \frac{{{\boldsymbol{\Delta }}_\lambda }}{2}$, a sufficient condition is

$$\begin{aligned} \theta \le \Box {{\boldsymbol{\Delta }}_\lambda }\mathbb {G}(T) - \Box _{\Vert \gamma \Vert _\infty ,\Vert g \Vert _1} \left( \sqrt{ \mathbb {G}(T)\log (|J |\alpha ^{-1})} + \log (|J |\alpha ^{-1})\right) . \end{aligned}$$

The other condition on θ is

$$\begin{aligned}&\theta \ge I(t_-) \\ \Leftarrow \quad&\theta \ge \Vert \gamma \Vert _\infty \mathbb {G}(t_-) \\ \Leftrightarrow \quad&\theta \ge \left( \Box (\Vert g \Vert _1 \vee 1) \log \left( \frac{2|J |}{\alpha }\right) \left( R^{-1/2} + \Vert \gamma \Vert _{\infty } R^{-1}\right) ^2\right) \vee \Vert \gamma \Vert _\infty \mathbb {G}(T_{\text {min}}) \\ \Leftarrow \quad&\theta \ge \left( \Box _{\Vert g \Vert _1} \log (|J |\alpha ^{-1}) \left( \frac{\Vert \gamma \Vert _\infty }{\sqrt{{{\boldsymbol{\Delta }}_\lambda }}}\vee \frac{\Vert \gamma \Vert _{\infty }^2}{{{\boldsymbol{\Delta }}_\lambda }}\right) \right) \vee \Vert \gamma \Vert _\infty \mathbb {G}(T_{\text {min}}) \end{aligned}$$

and finally, since by definition $T> T_{\text {min}}$,

$$\begin{aligned}&\mathbb {G}(t_-)< \mathbb {G}(T) \\ \Leftarrow \quad&\Box _{\Vert g \Vert _1} \log (|J |\alpha ^{-1}) \left( \frac{1}{\sqrt{{{\boldsymbol{\Delta }}_\lambda }}}\vee \frac{\Vert \gamma \Vert _{\infty }}{{{\boldsymbol{\Delta }}_\lambda }}\right) < \mathbb {G}(T) \end{aligned}$$

which concludes the proof.

### C.7 Proof of Theorem 3.6

Let $G : x \mapsto \int _0^x g(u)du$ and let $\Lambda ^{j,n}_{m,t} :=\int _0^t \lambda ^{j,n}_{m,s}ds$ be the compensator of the process $(N^{j,n}_{m,t})_{t\ge 0}$. We still denote $\bar{\lambda }^j_m :=\sum _{i\in I} \gamma ^i_o w^{i\rightarrow j}_m$.

Let $x=(x_l)_{1\le l\le d},y = (y_l)_{1\le l \le d},z = (z_l)_{1\le l \le d}$ vectors in $\mathbb {R}^d$. We denote by $(x,y) :=\sum _{l=1}^d x_l y_l$ the usual scalar product between two vectors, which we extend to three vectors by $(x,y,z) :=\sum _{l=1}^d x_l y_l z_l$.

We will need the following lemmas.

#### Lemma C.21

Let x>0. With the constants of Lemma C.2, if *g* has compact support on [0, *S*], then there exists i.i.d. standard Brownian motions $(B^i_t)_{t\ge 0}$’s independent of everything else such that with probability larger than $1-|I| be^{-dx}$, for every j∈J and t≥0,

$$\begin{aligned}&|\Lambda ^{j,n}_{m,t}- \mathbb {E}[\Lambda ^{j,n}_{m,t}] - \sum _{i\in I}w^{i\rightarrow j}_m B^i_{n\gamma ^i_o t}| \\&\le S n \bar{\lambda }^j_m \Vert g\Vert _1 + \sum _{i\in I} w^{i\rightarrow j}_m |B^i_{n\gamma ^i_o(t-S)}-B^i_{n\gamma ^i_o t}| + \log (n\Vert \gamma \Vert _{\infty }t\vee 1+1) (a+x), \end{aligned}$$

with $$\Vert \gamma \Vert _{\infty }=\max _{i\in I,o\in \mathcal {O}} \gamma ^i_o$$.

#### Proof

For all t≥0,

C94 $$\begin{aligned} n \bar{\lambda }^j_m \Vert g\Vert _1 (t-S) \le \mathbb {E}[\Lambda ^{j,n}_{m,t}] \le n \bar{\lambda }^j_m\Vert g\Vert _1 t. \end{aligned}$$

Moreover

C95 $$\begin{aligned} \Vert g\Vert _1 \sum _{i\in I} w^{i\rightarrow j}_m \Pi ^{i,n}_{m,t-S} \le \Lambda ^{j,n}_{m,t} \le \Vert g\Vert _1 \sum _{i\in I} w^{i\rightarrow j}_m \Pi ^{i,n}_{m,t}. \end{aligned}$$

The first part is just due to the fact that if t-s≥S then $G(t-s)=\Vert g\Vert _1$ and that $G(t-s)\le \Vert g\Vert _1$ for all *t* and *s*. The second part is done with exactly the same arguments. So we get for all t≥0 that

$$\begin{aligned}&\sum _{i\in I} w^{i\rightarrow j}_m [\Pi ^{i,n}_{m,t-S}-n\gamma ^i_o (t-S)] - S n \bar{\lambda }^j_m \Vert g\Vert _1 \\&\quad \le \Lambda ^{j,n}_{m,t}- \mathbb {E}(\Lambda ^{j,n}_{m,t}) \\&\quad \le \sum _{i\in I} w^{i\rightarrow j}_m [\Pi ^{i,n}_{m,t}-n\gamma ^i_o t] + S n \bar{\lambda }^j_m \Vert g\Vert _1. \end{aligned}$$

Now we apply Lemma C.2 to all $\tilde{\Pi }^i$ that are dilation of $(\Pi ^{i,n}_{m,t})_{t\ge 0}$ so that they are Poisson processes of rate 1. It means we obtain i.i.d. standard Brownian motions $B^i$, only depending on $\tilde{\Pi }^i$ and auxiliary i.i.d. uniform random variables $V^i$ such that with probability larger $1-|I| b e^{-dx}$,

C96 $$\begin{aligned} \forall i \in I, \forall t \ge 0, \left\lvert \Pi ^{i,n}_{m,t}-n\gamma ^i_o t - B^i_{n\gamma ^i_o t} \right\rvert \le \log (n \Vert \gamma \Vert _{\infty }t\vee 1+1) (a+x). \end{aligned}$$

We apply this at time *t* and t-S in the above equation and this leads to the desired result.

□

Now, let us choose $S \le n^{-1/2}$.

#### Proposition C.22

Let x>0, m≥1, $$o\in \mathcal {O}$$ the nature of object *m*. There are absolute positive constants $b^{\prime },d^{\prime }$ and a positive $a^{\prime }$ depending on $$\Vert \gamma \Vert _{\infty }$$ and $$\Vert g \Vert _1$$ such that there exists independent standard Brownian motions $$(B^j_t)_{t\ge 0}$$ such that with probability larger than $$1-(|I|+|J|) b'e^{-d'x}$$

C97 $$\begin{aligned}&\forall j\in J, \forall t\ge 0, \quad |N^{j,n}_{m,t}-\Lambda ^{j,n}_{m,t}-B^j_{n(w^j_l, \gamma ^I_o) \Vert g \Vert _1 t}|\nonumber \\&\quad \le a'n^{1/4}(t\vee 1)^{1/4} (\log (nt+1)(x\vee 1))^{3/4}. \end{aligned}$$

#### Proof

For all j∈J, let us consider $\pi ^j$ independent Poisson processes of rate 1, independent of everything else. By Lemma C.2, we can construct i.i.d. standard Brownian motions $(B^j_t)_{t\ge 0}$ such that with probability larger than $1-|J| be^{-dx}$, we have that

C98 $$\begin{aligned} \forall j\in J, \forall t\ge 0, |\pi ^j_t-t-B_t^j| \le \log (t \vee 1+1) (a+x). \end{aligned}$$

Since the (random) function $\Lambda ^{j,n}_{m,\cdot }$’s are non decreasing, we can always define their inverse $(\Lambda ^{j,n}_{m,\cdot })^{-1}$, conditionally to the inputs $\Pi ^{i,n}_{m}$’s, so that one can construct $N^{j,n}_m$, the Hawkes processes as $\pi ^j_{\Lambda ^{j,n}_{m,t}}$. Hence we have by change of time, that on the same event of probability larger than $1-|J| be^{-dx},$

C99 $$\begin{aligned} \forall j\in J, \forall t\ge 0, \left\lvert N^{j,n}_{m,t}-\Lambda ^{j,n}_{m,t}-B_{\Lambda ^{j,n}_{m,t}}^j \right\rvert \le \log (\Lambda ^{j,n}_{m,t} \vee 1+1) (a+x). \end{aligned}$$

Now we can apply Lemma C.21, so that with probability larger than $1-(|I|+|J|) b e^{-dx}$, both the above equations and the result of Lemma C.21 holds.

Now let us intersect with the event where the results of Lemma C.9 hold for all i∈I and j∈J.

By a union bound, we get therefore that on an event of probability larger than $1- (|I|+|J|) (a+c') e^{- d\wedge c" x},$

- with $B^i$ at time $n\gamma ^i_o t$ and u=0,
  C100 $$\begin{aligned} |B^i_{n\gamma ^i_o t}| \le c \sqrt{(n\gamma ^i_ot+2) (\log (n\gamma ^i_ot+1)+2x)} = \square _{\Vert \gamma \Vert _\infty } \sqrt{nt(\log (nt+1)+x)} \end{aligned}$$
- still with $B^i$ at time $n\gamma ^i_o t$ and $n\gamma ^i_o (t-S)$,
  C101 $$\begin{aligned} \left\lvert B^i_{n\gamma ^i_o(t-S)}-B^i_{n\gamma ^i_ot} \right\rvert&\le c \sqrt{(n\gamma ^i_o S+2) (2\log (n\gamma ^i_ot+1)+2x)} \nonumber \\&= \square _{\Vert \gamma \Vert _\infty } \sqrt{n^{1/2}(\log (nt+1)+x)} . \end{aligned}$$

Hence on the same event,

$$\begin{aligned} \forall j\in J, \forall t \ge 0, |\Lambda ^{j,n}_{m,t}-\mathbb {E}[\Lambda ^{j,n}_{m,t}]|\le \square _{\Vert \gamma \Vert _\infty ,\Vert g \Vert _1} \sqrt{n(t\vee 1)\log (nt+1)(x\vee 1)}. \end{aligned}$$

Besides, by (C94) we also have

C102 $$\begin{aligned} \forall j\in J, \forall t \ge 0, \left\lvert \mathbb {E}[\Lambda ^{j,n}_{m,t}] - n \bar{\lambda }^j_m \Vert g \Vert _1 t \right\rvert \le \Box _{\Vert \gamma \Vert _\infty ,\Vert g \Vert _1} \sqrt{n} \end{aligned}$$

so we have

C103 $$\begin{aligned} \forall j\in J, \forall t \ge 0, |\Lambda ^{j,n}_{m,t}- n \bar{\lambda }^j_m \Vert g \Vert _1 t|\le \square _{\Vert \gamma \Vert _\infty ,\Vert g \Vert _1} \sqrt{n(t\vee 1)\log (nt+1)(x\vee 1)}. \end{aligned}$$

Going back to (C99) and now using Lemma C.9 with j∈J at time $\Lambda ^{j,n}_{m,t}$ and $n \bar{\lambda }^j_m \Vert g \Vert _1 t$, we therefore obtain that

$$\begin{aligned}&\left\lvert B_{\Lambda ^{j,n}_{m,t}}^j-B_{n\bar{\lambda }^j_m \Vert g \Vert _1 t}^j \right\rvert \\&\le c\sqrt{\left( |n \bar{\lambda }^j_m \Vert g \Vert _1 t-\Lambda ^{j,n}_{m,t}|+2\right) \left( \log (n \bar{\lambda }^j_m \Vert g \Vert _1 t+1)+\log (\Lambda ^{j,n}_{m,t}+1)+2x\right) } \end{aligned}$$

and because (C103) on the same event, we have $\Lambda ^{j,n}_{m,t} \le \square _{\gamma ,\Vert g \Vert _1} nt$. Moreover using (C103) again, we get that on the same event,

C104 $$\begin{aligned} |B_{\Lambda ^{j,n}_{m,t}}^j-B_{n \bar{\lambda }^j_m \Vert g \Vert _1 t}^j| \le \square _{\Vert \gamma \Vert _\infty ,\Vert g \Vert _1} (n (t\vee 1))^{1/4}(\log (nt+1)(x\vee 1))^{3/4}. \end{aligned}$$

Injecting this in (C99), we obtain the result. □

Now we can prove the theorem. Let us work on the same event as the one of the proof of Proposition C.22, for which the conclusions of Lemma C.21 also apply, which has probability more than $1-(|I |+ |J |)b'e^{-d'x}$. On this event, according to Lemma C.21 we have for every j∈J and t≥0

$$\begin{aligned}&\left\lvert \Lambda ^{j,n}_{m,t}- \mathbb {E}(\Lambda ^{j,n}_{m,t}) - \sum _{i\in I}w^{i\rightarrow j}_m B^i_{n \gamma ^i_o t} \right\rvert \\&\le \Vert g \Vert _1\Vert \gamma \Vert _\infty n^{-1/2} + \sum _{i\in I} w^{i\rightarrow j}_m |B^i_{n\gamma ^i_o(t-S)}\\&\quad -B^i_{n\gamma ^i_ot}| + \Box _{\Vert \gamma \Vert _\infty }\log ((nt)\vee 1+1)(x\vee 1). \end{aligned}$$

Besides, according to (C101) on the same event we have for every i∈I

$$ |B^i_{n\gamma ^i_o(t-S)}-B^i_{n\gamma ^i_ot}| \le \square _{\Vert \gamma \Vert _\infty } \sqrt{n^{1/2}(\log (nt+1)+x)}. $$

Since $\sum _{i\in I} w^{i\rightarrow j}_m = 1$ we have then for every j∈J

$$\begin{aligned}&\left\lvert \Lambda ^{j,n}_{m,t}- \mathbb {E}(\Lambda ^{j,n}_{m,t}) - \sum _{i\in I}w^{i\rightarrow j}_m B^i_{n \gamma ^i_o t} \right\rvert \le \Box _{\Vert g \Vert _1,\Vert \gamma \Vert _\infty } \sqrt{n^{1/2}\log (nt +1)(x\vee 1)} . \end{aligned}$$

Therefore, by combining this with the conclusions of Proposition C.22, we have that on the same event, for every j∈J and t≥0,

$$\begin{aligned}&\left\lvert N^{j,n}_{m,t} - \mathbb {E}[\Lambda ^{j,n}_{m,t}] - \sum _{i\in I}w^{i\rightarrow j}_m B^i_{n \gamma ^i_o t} - B^j_{ n \Vert g \Vert _1 t\bar{\lambda }^j_m} \right\rvert \\&\hspace{3cm}\le \Box _{\Vert g \Vert _1,\Vert \gamma \Vert _\infty } n^{1/4}(t\vee 1)^{1/4} \left( \log (nt+1)(x\vee 1)\right) ^{3/4}. \end{aligned}$$

By construction, the processes $((B^l_u)_{u\ge 0})_{l\in I\cup J}$ are mutually independent. We have then the following equality in distribution:

$$\begin{aligned} \sum _{i\in I}w^{i\rightarrow j}_m B^i_{n \gamma ^i_o t} + B^j_{n\bar{\lambda }^j_m \Vert g \Vert _1 t}&\overset{\mathcal {D}}{=} \sum _{i\in I}w^{i\rightarrow j}_m \sqrt{n \gamma ^i_o}B^i_{ t} + \sqrt{n \bar{\lambda }^j_m\Vert g \Vert _1}B^j_{t} \end{aligned}$$

Therefore, the process $W^{j,n}_{m,t} :=\mathbb {E}[\Lambda ^{j,n}_{m,t}] + \sum _{i\in I}w^{i\rightarrow j}_m B^i_{n \gamma ^i_o t} + B^j_{n \bar{\lambda }^j_m\Vert g \Vert _1 t}$ is a Gaussian process with mean

C105 $$\begin{aligned} \mathbb {E}[W^{j,n}_{m,t}] = \mathbb {E}[\Lambda ^{j,n}_{m,t}] \end{aligned}$$

and covariance

$$\begin{aligned} \mathop {\text {Cov}}(W^{j,n}_{m,t}, W^{j,n}_{m,s})&= \sum _{i\in I} (w^{i\rightarrow j}_m)^2 n\gamma ^i_o \mathop {\text {Cov}}(B^i_t, B^i_s) + n\Vert g \Vert _1 \sum _{i\in I} w^{i\rightarrow j}_m \gamma ^i_o \mathop {\text {Cov}}(B^j_t, B^j_s) \\&= n t\wedge s \left( (w^j_m, w^j_m, \gamma ^I_o) + \Vert g \Vert _1 \bar{\lambda }^j_m\right) . \end{aligned}$$

Furthermore, if $j^{\prime }\neq j$ then

$$\begin{aligned} \mathop {\text {Cov}}(W^{j,n}_{m,t}, W^{j',n}_{m,s})&= \sum _{i\in I} w^{i\rightarrow j}_m w^{i\rightarrow j'}_m n\gamma ^i_o \mathop {\text {Cov}}(B^i_t, B^i_s) \\&= n (t\wedge s)(w^j_m, w^{j'}_m, \gamma ^I_o). \end{aligned}$$

Let us choose *x* such that $(|I |+ |J |)b'e^{-d'x} = \alpha$, *i.e.,* $x = \frac{1}{d'}\log \left( \frac{(|I |+|J |)b'}{\alpha }\right)$. Then for $b'\ge \Box$, we have x≥1 and with probability more than 1-α, for every j∈J we have

C106 $$\begin{aligned} \sup _{t> 0}\frac{ \left\lvert N^{j,n}_{m,t} - W^{j,n}_{m,t} \right\rvert }{n^{1/4}(t\vee 1)^{1/4}\log (nt + 1)^{3/4}} \le \Box _{\Vert g \Vert _1,\Vert \gamma \Vert _\infty } \log \left( \frac{|I |+|J |}{\alpha }\right) ^{3/4}. \end{aligned}$$

Now let us study the hitting times. Let us work on the same event that we denote $\Omega _1$. Let $U:=\max _{j\in J} \frac{\theta }{\bar{\lambda }^j_m \Vert g \Vert _1} + 1$. Then for every j∈J and t≥0 we have

C107 $$\begin{aligned} \left\lvert N^{j,n}_{m,t} - \mathbb {E}[N^{j,n}_{m,t}] \right\rvert \le \left\lvert \tilde{B}^{j,n}_{m,t} \right\rvert + \Box _{\Vert g \Vert _1,\Vert \gamma \Vert _\infty } n^{1/4}(t\vee 1)^{1/4} \log (nt +1)^{3/4} \log \left( \frac{|I |+|J |}{\alpha }\right) ^{3/4} \end{aligned}$$

where $\tilde{B}^{j,n}_{m,t}$ = $\sum _{i\in I}w^{i\rightarrow j}_m B^i_{n \gamma ^i_o t} + B^j_{n\bar{\lambda }^j_m \Vert g \Vert _1 t}$. Then $(\tilde{B}^{j,n}_{m,t})_{t\ge 0}$ is a Brownian motion with 0 drift and scaling $\sigma _n :=\sqrt{ n \left( (w^j_m, w^j_m, \gamma ^I_o) + \Vert g \Vert _1 \bar{\lambda }^j_m\right) } \le \Box _{\Vert g \Vert _1,\Vert \gamma \Vert _\infty }\sqrt{n}$. According to Lemma C.1, by union bound there exists an event $\Omega _2$ with probability more than 1-α such that on $\Omega _2$,

C108 $$\begin{aligned} \forall j\in J, \quad \sup _{0\le t \le U} \left\lvert \tilde{B}^{j,n}_{m,t} \right\rvert < \Box _{\Vert \gamma \Vert _\infty ,\Vert g \Vert _1} \sqrt{nU\log (|J |\alpha ^{-1})}. \end{aligned}$$

Let us work on $\Omega _1 \cap \Omega _2$. We can now control $\left\lvert N^{j,n}_{m,t} - \mathbb {E}[N^{j,n}_{m,t}] \right\rvert$. Furthermore, $\mathbb {E}[N^{j,n}_{m,t}] = \mathbb {E}[\Lambda ^{j,n}_{m,t}]$ and by (C94) we have $\left\lvert \mathbb {E}[\Lambda ^{j,n}_{m,t}] - n\bar{\lambda }^j_m \Vert g \Vert _1 t \right\rvert \le \Box _{\Vert g \Vert _1,\Vert \gamma \Vert _\infty }\sqrt{n}$. By combining everything, we get that for every j∈J and t∈[0,U]

C109 $$\begin{aligned}&\left\lvert N^{j,n}_{m,t} - n\bar{\lambda }^j_m \Vert g \Vert _1 t \right\rvert \nonumber \\&\le \Box _{\Vert \gamma \Vert _\infty ,\Vert g \Vert _1}\left( \sqrt{nU\log (|J |\alpha ^{-1})} + n^{1/4} U^{1/4} \log (nU + 1)^{3/4}\log \left( \frac{|I |+|J |}{\alpha }\right) ^{3/4}\right) \nonumber \\&\le \Box _{\Vert \gamma \Vert _\infty ,\Vert g \Vert _1} \sqrt{nU} \log \left( \frac{|I |+|J |}{\alpha }\right) ^{3/4}. \end{aligned}$$

Since $W^{j,n}_{m,t} = \mathbb {E}[N^{j,n}_{m,t}] + \tilde{B}^{j,n}_{m,t}$ and $\mathbb {E}[N^{j,n}_{m,t}] = \mathbb {E}[W^{j,n}_{m,t}]$, similarly we have

C110 $$\begin{aligned} \forall j\in J, \forall t \in [0,U], \left\lvert W^{j,n}_{m,t} - n\bar{\lambda }^j_m \Vert g \Vert _1 t \right\rvert \le \Box _{\Vert \gamma \Vert _\infty ,\Vert g \Vert _1}\sqrt{nU\log (|J |\alpha ^{-1})}. \end{aligned}$$

∙ Study of the hitting times of $W^{j,n}_{m,t}$ and $N^{j,n}_{m,t}$.

Let $\tilde{\tau }_n(W^j):=\inf \{u\in [0,U], W^{j,n}_{m,u} \ge n\theta \}$ and $\tilde{\tau }_n(N^j)= \inf \{u\in [0,U], N^{j,n}_{m,u} \ge n\theta \}$. First, let us study under which conditions $\tilde{\tau }_n(N^j)<\infty$ and $\tilde{\tau }_n(W^j)<\infty$. According to (C109), we have that for all j∈J,

$$\begin{aligned} N^{j,n}_{m,U}&\ge n\bar{\lambda }^j_m\Vert g \Vert _1 U - \Box _{\Vert \gamma \Vert _\infty ,\Vert g \Vert _1} \sqrt{nU} \log \left( \frac{|I |+|J |}{\alpha }\right) ^{3/4}. \end{aligned}$$

Then a sufficient condition to have $\tilde{\tau }_n(N^j)<\infty$ is to have

C111 $$\begin{aligned} n \bar{\lambda }^j_m \Vert g \Vert _1 U - \Box _{\Vert \gamma \Vert _\infty ,\Vert g \Vert _1} \sqrt{nU} \log \left( \frac{|I |+|J |}{\alpha }\right) ^{3/4} > n\theta , \end{aligned}$$

which is implied by

C112 $$\begin{aligned} U > \frac{\theta }{\bar{\lambda }^j_m \Vert g \Vert _1} + \Box _{\Vert \gamma \Vert _\infty ,\Vert g \Vert _1, \bar{\lambda }_{\text {min}},\theta } n^{-1/2} \log \left( \frac{|I |+|J |}{\alpha }\right) ^{3/4} \end{aligned}$$

which holds for

C113 $$\begin{aligned} n\ge \Box _{\Vert \gamma \Vert _\infty ,\Vert g \Vert _1, \bar{\lambda }_{\text {min}},\theta } \log \left( \frac{|I |+|J |}{\alpha }\right) ^{3/2} \end{aligned}$$

by definition of *U*. By using (C110), we can apply the same reasoning to $W^{j,n}_{m}$ to find that (C112) is also a sufficient condition to have $\tilde{\tau }_n(W^j)<\infty$.

This means that on this event, both processes $W^{j,n}_m$ and $N^{j,n}_m$ have reached nθ before time *U*, so we have in fact $\tau _n(N^j)= \tilde{\tau }_n(N^j)$ and $\tau _n(W^j)= \tilde{\tau }_n(W^j)$. Therefore, for every j∈J and $Z\in \{N,W\}$, we have $\tau _n(Z^j) \le U$, and controlling the trajectory of $Z^{j,n}_m$ on [0, *U*] is sufficient to control it on $[0,\tau _n(Z^j)]$. Using this fact, let us now study the convergence of $\tau _n(N^j)$ and $\tau _n(W^j)$ to $\frac{\theta }{\bar{\lambda }^j_m \Vert g \Vert _1}$.

1. Convergence of $\tau _n(N^j)$. Since $N^{j,n}_{m,\tau _n(N^j)}$ is an integer and cannot make two jumps at the same time, we have $N^{j,n}_{m,\tau _n(N^j)} = \lceil n\theta \rceil$. Therefore, for every j∈J we have

$$\begin{aligned} \left\lvert n\theta - n \bar{\lambda }^j_m \Vert g \Vert _1 \tau _n(N^j) \right\rvert&\le \left\lvert n\theta - \lceil n\theta \rceil \right\rvert + \left\lvert N^{j,n}_{m,\tau _n(N^j)} - n \bar{\lambda }^j_m \Vert g \Vert _1 \tau _n(N^j) \right\rvert \\&\le 1 + \Box _{\Vert \gamma \Vert _\infty ,\Vert g \Vert _1} \sqrt{nU} \log \left( \frac{|I |+|J |}{\alpha }\right) ^{3/4} \end{aligned}$$

were the last inequality holds using (C109). So we have

C114 $$\begin{aligned} \left\lvert \tau _n(N^j)- \frac{\theta }{\bar{\lambda }^j_m \Vert g \Vert _1} \right\rvert \le \Box _{\Vert \gamma \Vert _\infty ,\Vert g \Vert _1, \bar{\lambda }_{\text {min}},\theta } n^{-1/2}\log \left( \frac{|I |+|J |}{\alpha }\right) ^{3/4}. \end{aligned}$$

2. Convergence of $\tau _n(W^j)$. According to (C110) we have

$$\begin{aligned} \left\lvert n\theta - n \bar{\lambda }^j_m \Vert g \Vert _1 \tau _n(W^j) \right\rvert&= \left\lvert W^{j,n}_{m,\tau _n(N^j)} - n \bar{\lambda }^j_m \Vert g \Vert _1 \tau _n(W^j) \right\rvert \\&\le \Box _{\Vert \gamma \Vert _\infty ,\Vert g \Vert _1}\sqrt{nU\log (|J |\alpha ^{-1})} \end{aligned}$$

so

C115 $$\begin{aligned} \left\lvert \tau _n(W^j)- \frac{\theta }{\bar{\lambda }^j_m \Vert g \Vert _1} \right\rvert \le \Box _{\Vert \gamma \Vert _\infty ,\Vert g \Vert _1, \bar{\lambda }_{\text {min}},\theta } \sqrt{\frac{1}{n}\log (|J |\alpha ^{-1})} \end{aligned}$$

3. Study of the difference $\left\lvert \tau _n(N^j)- \tau _n(W^j) \right\rvert$. Let us begin by bounding $\left\lvert W^{j,n}_{m,\tau _n(N^j)} - W^{j,n}_{m,\tau _n(W^j)} \right\rvert$. We have

$$\begin{aligned} \left\lvert W^{j,n}_{m,\tau _n(N^j)} - W^{j,n}_{m,\tau _n(W^j)} \right\rvert&= \left\lvert W^{j,n}_{m,\tau _n(N^j)} - n\theta \right\rvert \\&\le \left\lvert W^{j,n}_{m,\tau _n(N^j)} - \lceil n\theta \rceil \right\rvert + | \lceil n\theta \rceil - n\theta |\\&\le \left\lvert W^{j,n}_{m,\tau _n(N^j)} - \Pi ^{j,n}_{m,\tau _n(N^j)} \right\rvert + 1 \\ \end{aligned}$$

so according to (C106) we have

C116 $$\begin{aligned} \left\lvert W^{j,n}_{m,\tau _n(N^j)} - W^{j,n}_{m,\tau _n(W^j)} \right\rvert \le \Box _{\Vert \gamma \Vert _\infty ,\Vert g \Vert _1, \bar{\lambda }_{\text {min}},\theta } n^{1/4}\log (n)^{3/4} \log \left( \frac{|I |+|J |}{\alpha }\right) ^{3/4} \end{aligned}$$

Besides,

$$\begin{aligned} \left\lvert W^{j,n}_{m,\tau _n(N^j)} - W^{j,n}_{m,\tau _n(W^j)} \right\rvert&= \left\lvert n\bar{\lambda }^j_m\left( \mathbb {G}(\tau _n(N^j)) - \mathbb {G}(\tau _n(W^j))\right) + \left( \tilde{B}^{j,n}_{m,\tau _n(N^j)}-\tilde{B}^{j,n}_{m,\tau _n(W^j)}\right) \right\rvert \end{aligned}$$

Besides, if $t_1 \ge t_2 \ge n^{-1/2}$ then

$$\begin{aligned} \mathbb {G}(t_1) - \mathbb {G}(t_2)&= \int _{s = t_2}^{t_1} \int _{u=0}^s g(u) du ds \\&= \int _{s = t_2}^{t_1} \Vert g \Vert _1 ds \\&= \Vert g \Vert _1(t_1 - t_2). \end{aligned}$$

Since $\tau _n(N^j)$ and $\tau _n(W^j)$ both converge to $\frac{\theta }{\bar{\lambda }^j_m \Vert g \Vert _1} >0$, so for *n* verifying (C113) we have $\tau _n(N^j)\ge n^{-1/2}$ and $\tau _n(W^j)\ge n^{-1/2}$ so $|\mathbb {G}(\tau _n(N^j)) - \mathbb {G}(\tau _n(W^j)) | = |\tau _n(N^j)- \tau _n(W^j) |\Vert g \Vert _1$. Therefore

$$\begin{aligned}&\left\lvert W^{j,n}_{m,\tau _n(N^j)} - W^{j,n}_{m,\tau _n(W^j)} \right\rvert \ge n\bar{\lambda }^j_m \Vert g \Vert _1 |\tau _n(N^j)- \tau _n(W^j) | - \left\lvert \tilde{B}^{j,n}_{m,\tau _n(N^j)}- \tilde{B}^{j,n}_{m,\tau _n(W^j)} \right\rvert \end{aligned}$$

According to Lemma C.9 and since $\tau _n(W^j)$ and $\tau _n(N^j)$ are bounded on $\Omega _1\cap \Omega _2$, by union bound there exists an event $\Omega _3$ of probability more than 1-α such that on $\Omega _1\cap \Omega _2\cap \Omega _3$ we have for every j∈J

C117 $$\begin{aligned}&\left\lvert \tilde{B}^{j,n}_{m,\tau _n(N^j)} - \tilde{B}^{j,n}_{m,\tau _n(W^j)} \right\rvert \nonumber \\&\quad \le \Box _{\Vert \gamma \Vert _\infty ,\Vert g \Vert _1, \bar{\lambda }_{\text {min}},\theta } \sqrt{\left( \left( n\left\lvert \tau _n(N^j)- \tau _n(W^j) \right\rvert \right) \vee 1\right) \log (n|J |\alpha ^{-1})}. \end{aligned}$$

If $\left( n\left\lvert \tau _n(N^j)- \tau _n(W^j) \right\rvert \right) \vee 1 = 1$ then $\left\lvert \tau _n(N^j)- \tau _n(W^j) \right\rvert \le 1/n$ and we have the result. Otherwise we have

$$\begin{aligned}&\left\lvert W^{j,n}_{m,\tau _n(N^j)} - W^{j,n}_{m,\tau _n(W^j)} \right\rvert \\&\ge n \bar{\lambda }^j_m|\tau _n(N^j)- \tau _n(W^j) | - \Box _{\Vert \gamma \Vert _\infty ,\Vert g \Vert _1, \bar{\lambda }_{\text {min}},\theta }\\&\quad \sqrt{n\left\lvert \tau _n(N^j)- \tau _n(W^j) \right\rvert \log (n|J |\alpha ^{-1})} . \end{aligned}$$

Combining this with (C116) we have

$$\begin{aligned}&\Box _{\Vert \gamma \Vert _\infty ,\Vert g \Vert _1, \bar{\lambda }_{\text {min}},\theta } n^{1/4}\log (n)^{3/4} \log \left( \frac{|I |+|J |}{\alpha }\right) ^{3/4} \\&\ge n|\tau _n(N^j)- \tau _n(W^j) | - \Box _{\Vert \gamma \Vert _\infty ,\Vert g \Vert _1, \bar{\lambda }_{\text {min}},\theta }\sqrt{n\left\lvert \tau _n(N^j)- \tau _n(W^j) \right\rvert \log (n|J |\alpha ^{-1})} . \end{aligned}$$

By writing $X :=\sqrt{ n|\tau _n(N^j)- \tau _n(W^j) |}$, $a(n):=\Box _{\Vert \gamma \Vert _\infty ,\Vert g \Vert _1, \bar{\lambda }_{\text {min}},\theta }\sqrt{\log (n|J |\alpha ^{-1})}$ and $b(n) :=\Box _{\Vert \gamma \Vert _\infty ,\Vert g \Vert _1, \bar{\lambda }_{\text {min}},\theta } n^{1/4}\log (n)^{3/4} \log \left( \frac{|I |+|J |}{\alpha }\right) ^{3/4}$, we can rewrite it as

$$\begin{aligned} X^2 - a(n)X - b(n) \le 0. \end{aligned}$$

This polynomial has two real roots, the largest one being $x_0 = \frac{a(n)+\sqrt{a(n)^2 + 4b(n)}}{2}$, so necessarily we have $X\le x_0$, which implies that $X\le a(n) + \sqrt{b(n)}$, *i.e., *

$$\begin{aligned}&\sqrt{ n|\tau _n(N^j)- \tau _n(W^j) |} \\ &\quad \le \Box _{\Vert \gamma \Vert _\infty ,\Vert g \Vert _1, \bar{\lambda }_{\text {min}},\theta } \left( \sqrt{\log (n|J |\alpha ^{-1})} + n^{1/8}\log (n)^{3/8} \log \left( \frac{|I |+|J |}{\alpha }\right) ^{3/8} \right) \\&\quad \le \Box _{\Vert \gamma \Vert _\infty ,\Vert g \Vert _1, \bar{\lambda }_{\text {min}},\theta } n^{1/8}\log (n)^{3/8} \log \left( \frac{|I |+|J |}{\alpha }\right) ^{3/8} \end{aligned}$$

and finally we get

$$\begin{aligned} |\tau _n(N^j)- \tau _n(W^j) |&\le \Box _{\Vert \gamma \Vert _\infty ,\Vert g \Vert _1, \bar{\lambda }_{\text {min}},\theta } \left( \frac{\log (n)}{n}\right) ^{3/4} \log \left( \frac{|I |+|J |}{\alpha }\right) ^{3/4} . \end{aligned}$$

Since $\mathbb {P}(\Omega _1\cap \Omega _2 \cap \Omega _3) \ge 1-3\alpha$, we get the results of the theorem by choosing α/3 instead of α.

### C.8 Proof of Proposition 4.1

Let $j_1$ and $j_2$ the two categories, $i_1$ the feature characterizing category $j_1$ and $i_2$ the feature characterizing category $j_2$. Let us compute the feature discrepancies of the features. Since every $o\in j_1$ has feature $i_1$, for every $o\in j_1$ we have $\gamma ^{i_1}_o = \gamma$ and since there is no $o\in j_2$ having feature $i_1$, for every $o\in j_2$ we have $\gamma ^{i_1}_o = 0$. Therefore

C118 $$\begin{aligned} d^{i_1 \rightarrow j_1} = \gamma . \end{aligned}$$

Similarly, for every $i\in j_2$ we have $\gamma ^{i_2}_o = \gamma$ and for every $o\in j_1$ we have $\gamma ^{i_2}_o = 0$ so

C119 $$\begin{aligned} d^{i_2 \rightarrow j_1} = - \gamma < d^{i_1 \rightarrow j_1} \end{aligned}$$

Let $i\in I\setminus \{i_1,i_2\}$. Then there are 4 rockets among the 16 having simultaneously features $i_1$ and *i*. Since $j_1$ contains 5 objects, there is at least one object without feature *i* in $j_1$. Therefore $\langle \gamma ^i_o \rangle _{o\in j_1} < \gamma$ and

C120 $$\begin{aligned} d^{i\rightarrow j_1} < \gamma = d^{i_1 \rightarrow j_1}. \end{aligned}$$

Thus $I^{j_1} = \arg \max _{i\in I} d^{i\rightarrow j_1} = \{i_1\}$ and by definition of the family $w_\infty$, for i∈I we have $w^{i\rightarrow j_1} = 1\!\!1_{i=i_1}$. By symmetry, the same computations are true when exchanging index 1 with index 2 so for i∈I we also have $I^{j_1} = \{i_2\}$ and $w^{i\rightarrow j_2} = 1\!\!1_{i=i_2}$. Now, let us compute the $\bar{\lambda }^j_{\infty , o}$. For $o\in j_1$,

C121 $$\begin{aligned} \bar{\lambda }^{j_1}_{\infty , o} = \sum _{i\in I} w^{i\rightarrow j_1}_\infty \gamma ^i_o = \gamma ^{i_1}_o = \gamma \end{aligned}$$

and

C122 $$\begin{aligned} \bar{\lambda }^{j_2}_{\infty , o} = \sum _{i\in I} w^{i\rightarrow j_2}_\infty \gamma ^i_o = \gamma ^{i_2}_o = 0 \end{aligned}$$

so

C123 $$\begin{aligned} \bar{\lambda }^{j_1}_{\infty , o} > \bar{\lambda }^{j_2}_{\infty , o} + {{\boldsymbol{\Delta }}_\lambda }\end{aligned}$$

where ${{\boldsymbol{\Delta }}_\lambda }$ is any constant in (0,γ). By symmetry, for $o\in j_2$ we also have

C124 $$\begin{aligned} \bar{\lambda }^{j_2}_{\infty , o} > \bar{\lambda }^{j_1}_{\infty , o} + {{\boldsymbol{\Delta }}_\lambda }. \end{aligned}$$

## References

[CR1] Brunton BW, Botvinick MM, Brody CD (2013) Rats and humans can optimally accumulate evidence for decision-making. Sci 340(6128):95–98 10.1126/science.1233912 23559254

[CR2] Besançon E, Coutin L, Decreusefond L, Moyal P (2024) Diffusive limits of Lipschitz functionals of Poisson measures. Ann Appl Probab 34(1A):555–584. 10.1214/23-aap1972

[CR3] Bacry E, Delattre S, Hoffmann M, Muzy JF (2013) Some limit theorems for Hawkes processes and application to financial statistics. Stochastic Process Appl 123(7):2475–2499. 10.1016/j.spa.2013.04.007

[CR4] Bao Y, Kuang Z, Peissig P, Page D, Willett R (2017) Hawkes process modeling of adverse drug reactions with longitudinal observational data. In: Proceedings of the 2nd Machine Learning for Healthcare Conference, vol. 68, pp 177–190

[CR5] Boucheron S, Lugosi G, Massart P (2013) Concentration inequalities. Oxford University Press, Oxford, p 481. 10.1093/acprof:oso/9780199535255.001.0001 (**A nonasymptotic theory of independence, With a foreword by Michel Ledoux**)

[CR6] Bretagnolle J, Massart P (1989) Hungarian constructions from the nonasymptotic viewpoint. Ann Probab 17(1):239–256

[CR7] Bacry E, Mastromatteo I, Muzy J-F (2015) Hawkes Processes in Finance. Market Microstruct and Liquidity 1(01):1550005

[CR8] Brody CD, Romo R, Kepecs A (2003) Basic mechanisms for graded persistent activity: discrete attractors, continuous attractors, and dynamic representations. Curr Opin Neurobiol 13(2):204–211 12744975 10.1016/s0959-4388(03)00050-3

[CR9] Cesa-Bianchi N, Lugosi G (2006) Prediction, learning, and games. Cambridge University Press, Cambridge

[CR10] Carrillo JA, Cordier S, Mancini S (2011) A decision-making Fokker-Planck model in computational neuroscience. J Math Biol 63(5):801–830. 10.1007/s00285-010-0391-3 21184081 10.1007/s00285-010-0391-3

[CR11] Caporale N, Dan Y (2008) Spike timing-dependent plasticity: a Hebbian learning rule. Annu Rev Neurosci 31:25–46 18275283 10.1146/annurev.neuro.31.060407.125639

[CR12] Chevallier J, Melnykova A, Tubikanec I (2021) Diffusion approximation of multi-class Hawkes processes: theoretical and numerical analysis. Adv in Appl Probab 53(3):716–756. 10.1017/apr.2020.73

[CR13] Ditterich J (2006) Stochastic models of decisions about motion direction: behavior and physiology. Neural Netw 19(8):981–1012 16952441 10.1016/j.neunet.2006.05.042

[CR14] Deco G, Martí D (2007) Deterministic analysis of stochastic bifurcations in multi-stable neurodynamical systems. Biol Cybernet 96(5):487–496. 10.1007/s00422-007-0144-6 10.1007/s00422-007-0144-6 17387505

[CR15] Ethier SN, Kurtz TG (1986) Markov processes. John Wiley & Sons, Inc, New York. 10.1002/9780470316658 (**Characterization and convergence**)

[CR16] Erny X, Löcherbach E, Loukianova D (2023) Strong error bounds for the convergence to its mean field limit for systems of interacting neurons in a diffusive scaling. Ann Appl Probab 33(5):3563–3586

[CR17] Fudenberg D, Strack P, Strzalecki T (2018) Speed, accuracy, and the optimal timing of choices. Am Econ Rev 108(12):3651–84. 10.1257/aer.20150742

[CR18] Gerstein GL, Bedenbaugh P, Aertsen AM (1989) Neuronal assemblies. IEEE Trans Biomed Eng 36(1):4–14 2646211 10.1109/10.16444

[CR19] Gao B, Pavel L (2018) On the Properties of the Softmax Function with Application in Game Theory and Reinforcement Learning arXiv:1704.00805

[CR20] Gold JI, Shadlen MN (2001) Neural computations that underlie decisions about sensory stimuli. Trends Cogn Sci 5(1):10–16 11164731 10.1016/s1364-6613(00)01567-9

[CR21] Hawkes AG (1971) Spectra of some self-exciting and mutually exciting point processes. Biometrika 58(1):83–90

[CR22] Hutcherson CA, Bushong B, Rangel A (2015) A neurocomputational model of altruistic choice and its implications. Neuron 87(2):451–462 26182424 10.1016/j.neuron.2015.06.031 PMC4947370

[CR23] Hebb DO (2005) The organization of behavior: a neuropsychological theory. Psychology press, New York

[CR24] Hansen NR, Reynaud-Bouret P, Rivoirard V (2015) Lasso and probabilistic inequalities for multivariate point processes. Bernoulli 21(1):83–143. 10.3150/13-BEJ562

[CR25] Hubel DH, Wiesel TN (1962) Receptive fields, binocular interaction and functional architecture in the cat’s visual cortex. J Physiol 160(1):106–154 14449617 10.1113/jphysiol.1962.sp006837 PMC1359523

[CR26] Insabato A, Pannunzi M, Rolls ET, Deco G (2010) Confidence-related decision making. J Neurophysiol 104(1):539–547 20393062 10.1152/jn.01068.2009

[CR27] Jaffard S, Vaiter S, Muzy A, Reynaud-Bouret P (2024) Provable local learning rule by expert aggregation for a Hawkes network. In: International Conference on Artificial Intelligence and Statistics, pp 1837–1845. PMLR

[CR28] Jaffard S, Vaiter S, Reynaud-Bouret P (2026) CHANI: correlation-based Hawkes aggregation of neurons with bio-inspiration. J Mach Learn 27(32):1–62

[CR29] Komlós J, Major P, Tusnády G (1975) An approximation of partial sums of independent ’s and the sample . I. Z Wahrscheinlichkeitstheorie und Verw Gebiete 32:111–131. 10.1007/BF00533093

[CR30] Komlós J, Major P, Tusnády G (1976) An approximation of partial sums of independent RV’s, and the sample DF. II. Z Wahrscheinlichkeitstheorie und Verw Gebiete 34(1):33–58. 10.1007/BF00532688

[CR31] Karatzas I, Shreve SE (1998) Brownian motion and stochastic calculus, vol 113, 2nd edn. Springer, New York. 10.1007/978-1-4612-0949-2

[CR32] LaBerge D (1994) Quantitative models of attention and response processes in shape identification tasks. J Math Psychol 38(2):198–243

[CR33] Le Cun Y, Boser B, Denker J, Henderson D, Howard R, Hubbard W, Jackel L (1989) Handwritten digit recognition with a back-propagation network. In: NeurIPS

[CR34] Love B, Medin D, Gureckis T (2004) Sustain: a network model of category learning. Psychol Rev 111:309–332 15065912 10.1037/0033-295X.111.2.309

[CR35] Lovric M (2025) International Encyclopedia of statistical science. Springer, Berlin, Heidelberg

[CR36] Machens CK, Romo R, Brody CD (2005) Flexible control of mutual inhibition: a neural model of two-interval discrimination. Sci 307(5712):1121–1124 10.1126/science.1104171 15718474

[CR37] Nosofsky RM (1986) Attention, similarity, and the identification-categorization relationship. J Exp Psychol Gen 115:39–57 2937873 10.1037//0096-3445.115.1.39

[CR38] Ost G, Reynaud-Bouret P (2023) Neural coding as a statistical testing problem. Math Neurosci Appl 3:4–33

[CR39] Papamakarios G, Murray I (2016) Fast -free inference of simulation models with Bayesian conditional density estimation. In: Lee D, Sugiyama M, Luxburg U, Guyon I, Garnett R (eds) Advances in neural information processing systems, vol 29. Curran Associates Inc

[CR40] Philiastides MG, Ratcliff R (2013) Influence of branding on preference-based decision making. Psychol Sci 24(7):1208–1215 23696199 10.1177/0956797612470701

[CR41] Prodhomme A (2020) Strong Gaussian approximation of metastable density-dependent Markov chains on large time scales arXiv:2010.06861v3

[CR42] Prodhomme A (2023) Strong Gaussian approximation of metastable density-dependent Markov chains on large time scales. Stochastic Process Appl 160:218–264

[CR43] Ratcliff R (1978) A theory of memory retrieval. Psychol Rev 85(2):59–108

[CR44] Ratcliff R (1981) A theory of order relations in perceptual matching. Psychol Rev 88(6):552–572

[CR45] Ratcliff R (1988) Continuous versus discrete information processing: modeling accumulation of partial information. Psychol Rev 95(2):238–255 3375400 10.1037/0033-295x.95.2.238

[CR46] Reynaud-Bouret P (2003) Adaptive estimation of the intensity of inhomogeneous Poisson processes via concentration inequalities. Probab Theory Related Fields 126(1):103–153

[CR47] Ratcliff R, Hasegawa YT, Hasegawa RP, Smith PL, Segraves MA (2007) Dual diffusion model for single-cell recording data from the superior colliculus in a brightness-discrimination task. J Neurophysiol 97(2):1756–1774 17122324 10.1152/jn.00393.2006 PMC2394732

[CR48] Ratcliff R, McKoon G (2008) The diffusion decision model: theory and data for two-choice decision tasks. Neural Comput 20(4):873–922 18085991 10.1162/neco.2008.12-06-420 PMC2474742

[CR49] Rosenblatt F (1957) The perceptron, a perceiving and recognizing automaton project para. Cornell Aeronautical Laboratory, Ithaca, New York

[CR50] Roxin A (2019) Drift-diffusion models for multiple-alternative forced-choice decision making. J Math Neurosci 9:5–23. 10.1186/s13408-019-0073-4 31270706 10.1186/s13408-019-0073-4 PMC6609930

[CR51] Ratcliff R, Rouder JN (1998) Modeling response times for two-choice decisions. Psychol Sci 9(5):347–356

[CR52] Robbins H, Siegmund D (1970) Boundary crossing probabilities for the Wiener process and sample sums. Ann Math Statist 41:1410–1429. 10.1214/aoms/1177696787

[CR53] Ratcliff R, Smith PL (2004) A comparison of sequential sampling models for two-choice reaction time. Psychol Rev 111(2):333–367 15065913 10.1037/0033-295X.111.2.333 PMC1440925

[CR54] Revuz D, Yor M (1999) Continuous martingales and Brownian motion, vol 293, 3rd edn. Springer, Berlin, p 602. 10.1007/978-3-662-06400-9 (**Grundlehren der mathematischen Wissenschaften [Fundamental Principles of Mathematical Sciences]**)

[CR55] Singer W, Engel AK, Kreiter AK, Munk MH, Neuenschwander S, Roelfsema PR (1997) Neuronal assemblies: necessity, signature and detectability. Trends Cogn Sci 1(7):252–261 21223920 10.1016/S1364-6613(97)01079-6

[CR56] Shadlen MN, Kiani R (2013) Decision making as a window on cognition. Neuron 80(3):791–806 24183028 10.1016/j.neuron.2013.10.047 PMC3852636

[CR57] Shadlen MN, Newsome WT (1996) Motion perception: seeing and deciding. Proc Natl Acad Sci USA 93(2):628–633 8570606 10.1073/pnas.93.2.628 PMC40102

[CR58] Stone JV (2018) Principles of neural information theory. Comput Neurosci and Metabolic Efficiency

[CR59] Smith PL, Van Zandt T (2000) Time-dependent Poisson counter models of response latency in simple judgment. Br J Math Stat Psychol 53(2):293–315 11109709 10.1348/000711000159349

[CR60] Tavanaei A, Ghodrati M, Kheradpisheh SR, Masquelier T, Maida A (2019) Deep learning in spiking neural networks. Neural Netw 111:47–63 30682710 10.1016/j.neunet.2018.12.002

[CR61] Türkyilmaz K, Lieshout MNM, Stein A (2013) Comparing the Hawkes and trigger process models for Aftershock sequences following the 2005 Kashmir earthquake. Math Geosci 45:149–164

[CR62] Wagenmakers E-J, Maas HLJ, Grasman RPPP (2007) An EZ-diffusion model for response time and accuracy. Psychonomic Bulletin & Rev 14(1):3–22 10.3758/bf03194023 17546727

[CR63] Zhou K, Zha H, Song L (2013) Learning social infectivity in sparse low-rank networks using multi-dimensional Hawkes processes. In: Carvalho CM, Ravikumar P (eds) Proceedings of the sixteenth international conference on artificial intelligence and statistics, proceedings of machine learning research, vol 31. PMLR, Scottsdale, Arizona, USA, pp 641–649

